# Repeated truncation of a modular antimicrobial peptide gene for neural context

**DOI:** 10.1371/journal.pgen.1010259

**Published:** 2022-06-17

**Authors:** Mark A. Hanson, Bruno Lemaitre

**Affiliations:** Global Health Institute, School of Life Science, École Polytechnique Fédérale de Lausanne (EPFL), Lausanne, Switzerland; Friday Harbor Laboratories: University of Washington, UNITED STATES

## Abstract

Antimicrobial peptides (AMPs) are host-encoded antibiotics that combat invading pathogens. These genes commonly encode multiple products as post-translationally cleaved polypeptides. Recent studies have highlighted roles for AMPs in neurological contexts suggesting functions for these defence molecules beyond infection. During our immune study characterizing the antimicrobial peptide gene *Baramicin*, we recovered multiple *Baramicin* paralogs in *Drosophila melanogaster* and other species, united by their N-terminal IM24 domain. Not all paralogs were immune-induced. Here, through careful dissection of the *Baramicin* family’s evolutionary history, we find that paralogs lacking immune induction result from repeated events of duplication and subsequent truncation of the coding sequence from an immune-inducible ancestor. These truncations leave only the IM24 domain as the prominent gene product. Surprisingly, using mutation and targeted gene silencing we demonstrate that two such genes are adapted for function in neural contexts in *D*. *melanogaster*. We also show enrichment in the head for independent *Baramicin* genes in other species. The *Baramicin* evolutionary history reveals that the IM24 *Baramicin* domain is not strictly useful in an immune context. We thus provide a case study for how an AMP-encoding gene might play dual roles in both immune and non-immune processes via its multiple peptide products. As many AMP genes encode polypeptides, a full understanding of how immune effectors interact with the nervous system will require consideration of all their peptide products.

## Author summary

Antimicrobial peptides are immune proteins that directly combat infection, found across all animals. Antimicrobial peptides have long been implicated in neurological roles, though the ways these genes accomplish either immune or neurological function is poorly understood. One aspect of antimicrobial peptide genes that has received less attention is the fact that many genes encode multiple gene products on a precursor protein (including fruit fly *Defensin*, *Attacin*, *Diptericin*, *Drosocin*, or *Baramicin*). Here we show how the fruit fly *Baramicin* gene family has evolved for either immune-specific or neurological roles.

One sub-peptide type (IM10-like) is repeatedly lost in genes lacking immune induction that are enriched in nerve tissue. In these nervous system-specific genes, a different sub-peptide is uniquely retained (IM24). This pattern has happened repeatedly across different species and gene lineages, suggesting the ancestral gene was equipped with specific sub-peptides adapted for either role. These findings suggest some antimicrobial peptide genes might accomplish alternative roles in immunity or neurology by different actions of their sub-peptides. It will be interesting to reflect on these findings in the light of inflammatory diseases, as many human neuropeptides and antimicrobial peptides have multiple mature products.

## Introduction

Antimicrobial peptides (AMPs) are immune effectors best known for their role in defence against infection. These antimicrobials are commonly encoded as a polypeptide including both pro- and mature peptide domains [1,2]. AMP genes frequently experience events of duplication and loss [3–6] and undergo rapid evolution at the sequence level [7–12]. The selective pressures that drive these evolutionary outcomes are likely the consequence of host-pathogen interactions [13]. However AMPs and AMP-like genes in many species have recently been implicated in non-immune roles in flies, nematodes, and humans, suggesting non-immune functions might help explain AMP evolutionary patterns.

For instance, Diptericins are membrane-disrupting antimicrobial peptides of flies (Diptera) that are required for defence against infection by *Providencia* bacteria [13,14]. It was therefore surprising that the *D*. *melanogaster* gene *Diptericin B (DptB)* affects memory processes [15]. In this study, *DptB* derived from the fly fat body (analogous to the mammalian liver) regulated the ability of the fly to form long-term memory associations [15]. Another AMP-like gene, *nemuri*, regulates fly sleep and promotes survival upon infection [16]. Studies in nematodes have also shown that an immune-induced polypeptide (NLP-29) binds to a G-protein coupled receptor (NPR-12) triggering neurodegeneration through activation of the NPR-12-dependent autophagy pathway [17], and injury triggers epidermal AMPs including NLP-29 to promote sleep [18]. *Drosophila* AMPs have also recently been shown to regulate behaviours after seeing parasitoid wasps [19], during feeding with different bacteria [20], or following infection [21]. In humans, the *Cathelicidin* gene encodes the AMP LL-37, which is implicated in glia-mediated neuroinflammation and Alzheimer’s disease [22,23]. Indeed recent evidence suggests Alzheimer’s disease is an infectious syndrome [24], though the importance of this process is debated [25]. Notably, AMPs share a number of properties with classic neuropeptides [26], further muddying the distinction between peptides of the immune and nervous systems.

We recently described a novel antifungal peptide gene of *Drosophila melanogaster* that we named *Baramicin A* (*BaraA*) [21]. A unique aspect of *BaraA* is its precursor protein structure, which encodes a polypeptide cleaved into multiple mature products by interspersed furin cleavage sites. The use of furin cleavage sites to produce more than one mature peptide from a single polypeptide precursor is widespread in animal AMP genes [2,27], including multiple peptide repeats in bees and other flies [12,28]. However, *BaraA* represents an exceptional case as many tandem repeat peptides are cleaved by furin from a single precursor protein, effectively resembling a “protein-based operon”. The immature precursor protein of *D*. *melanogaster BaraA* encodes three types of domains: an IM24 domain, three tandem repeats of IM10-like domains, and an IM22 domain. *BaraA* mutants are susceptible to infection by fungi, and *in vitro* experiments suggest the *BaraA* IM10-like peptides have antifungal activity [21]. The other *Baramicin* domains encoding IM22 and IM24 remain uncharacterized. Curiously, *BaraA* deficient flies also display an erect wing behavioural phenotype upon immune stimulation even in the absence of infection, suggesting that *BaraA* products could have non-microbial targets [21].

In this study, we describe the evolution of the Drosophilid *Baramicin* gene family. Three unique *Baramicin* genes (*BaraA*, *B*, *and C*) are present in the genome of *D*. *melanogaster*. Surprisingly, only *BaraA* is immune-induced, while *BaraB* and *BaraC* are enriched in the nervous system. Both *BaraB* and *BaraC* have truncations compared to the ancestral *Baramicin* gene, and these two genes effectively encode just the *Baramicin* IM24 domain. We found similar truncations in other species, and confirmed loss of immune expression for IM24-specific *Baramicins* of other species. We also confirmed enrichment in the head or nervous system for IM24-specific genes in *D*. *melanogaster* and other species. We resolved the genomic ancestry of the *Baramicins*, which confirmed that these repeated truncations creating IM24-specific genes came from independent events (convergent evolution). The complex ‘protein operon’ polypeptide nature of *Baramicin* draws attention to how different sub-peptides can be adapted to context-specific roles, like in immunity or neurology. Attention to the multiple peptide products of AMP genes could explain how these immune effectors affect both immune and neurological processes.

## Results

### Baramicin is an ancestral immune effector

The *Baramicin A* gene was only recently described as encoding antifungal effectors by our group [21], and another recent study also confirmed *Baramicin*’s important contribution to Toll immune defence [29]. These initial characterizations were done only in *D*. *melanogaster*, and focused on one *Baramicin* gene (*BaraA*). We will therefore first provide a basic description of the immune *Baramicins* of other species and also the larger *Baramicin* gene family of *D*. *melanogaster* to establish that this is a classical immune gene family. This is relevant to paralogous genes to be discussed later.

**Fig 1:**
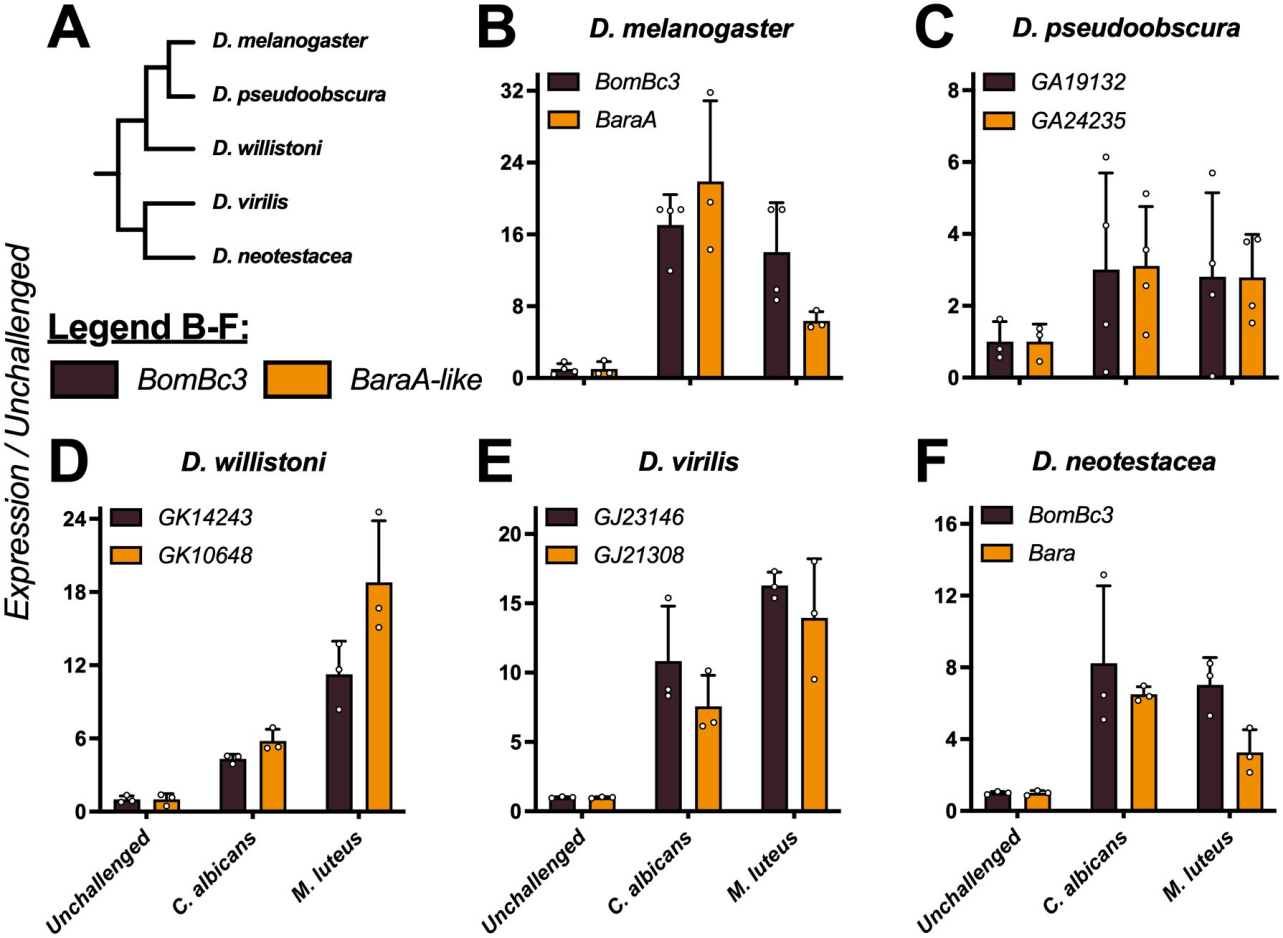
The ancestral *BaraA* gene was immune-induced. A) Cladogram of species used in B-F. B-F) Expression of the Toll-responsive gene *BomBc3* (brown) or *BaraA-like* genes (orange) in diverse *Drosophila* species upon infection. In all cases, both *BomBc3* and *BaraA-like* genes are induced upon infection by either *C*. *albicans* yeast or *M*. *luteus* bacteria.

In *D*. *melanogaster*, *BaraA* is regulated by the Toll immune signalling pathway [21,29]. Using BLAST, we recovered *BaraA*-like genes encoding each Baramicin peptide (IM24, IM10-like, and IM22) across the genus *Drosophila* and in the outgroup *Scaptodrosophila lebanonensis*. In many species, this was the only *Baramicin* gene present, suggesting *Dmel\BaraA* resembles the ancestral *Baramicin* structure. We performed infection experiments to confirm that *BaraA*-like genes were immune-inducible in the diverse species *D*. *melanogaster*, *D*. *pseudoobscura*, *D*. *willistoni*, *D*. *virilis*, and *D*. *neotesteacea* (last common ancestor ~47mya [30]) with *Micrococcus luteus* and *Candida albicans*, two microbes that stimulate the Toll pathway (Fig 1A). In all five species, *BaraA*-like genes were immune-induced (Fig 1B–1F). We therefore confirm the ancestral *Baramicin* was an immune-induced gene. Deviations from immune function are therefore derived.

### The D. melanogaster genome encodes up to four Baramicins: BaraA1, BaraA2, BaraB and BaraC

**Fig 2:**
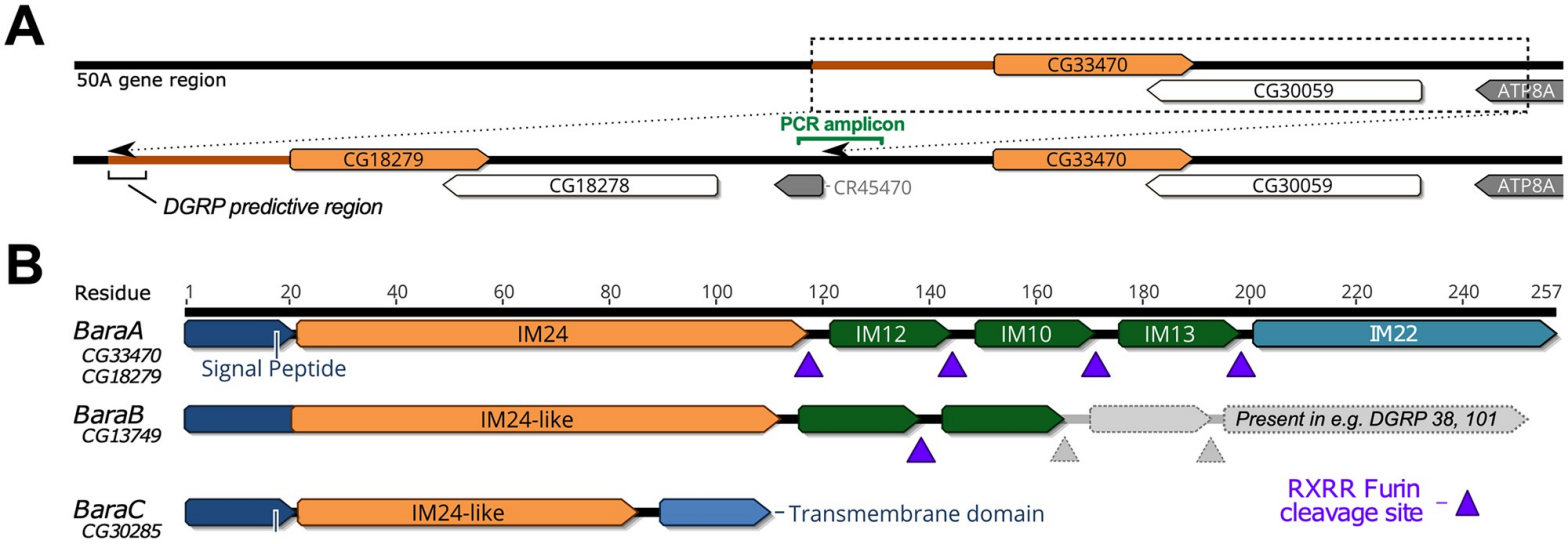
The *D*. *melanogaster Baramicin* genes. A) Schematic of the *BaraA* duplication. Using a PCR assay spanning the duplication-specific locus (PCR amplicon), we confirmed *BaraA* copy number is variable in various lab strains [21] and wild-caught flies (S1 Data). B) *D*. *melanogaster* encodes two other *Baramicin* genes that we name *BaraB* and *BaraC*. These paralogs differ markedly in their precursor protein structure through truncation of the C-terminus relative to *BaraA*. The *BaraB* truncation is segregating in the DGRP (greyed out region, and see S2 Data).

In *D*. *melanogaster*, we recovered four *Baramicin* genes. First, we realized that a duplication of *BaraA* is actively segregating in wild flies (Fig 2A). The *D*. *melanogaster* R6 genome assembly encodes two 100% identical *BaraA* genes (*CG33470* and *CG18279*, *BaraA1* and *BaraA2* respectively). We screened 132 DGRP lines for the *BaraA* duplication event, finding only ~14% (18/132) of strains were PCR-positive for two *BaraA* copies (S1 Data). Perhaps as a consequence of the identical sequences of these two genes, this genome region is poorly resolved in RNA sequencing studies and the Drosophila Genetic Reference Panel (DGRP, see S1 Fig) [31,32]. Because this region is poorly resolved, it is unclear if our PCR assay might be sensitive to cryptic sequence variation. However our PCR screen nevertheless confirms that this region is variable in the wild, and we additionally note that common fly strains seem to differ in their *BaraA* copy number, where extra gene copies correlates with increased expression after infection (see S10 Fig in [21]).

We also recovered two paralogous *Baramicin* genes in *D*. *melanogaster* through reciprocal BLAST searches: *CG13749* and *CG30285*, which we name *BaraB* and *BaraC* respectively (Fig 2B). The three *Baramicin* gene loci are scattered on the right arm of chromosome II at cytological positions 44F9 (*BaraB*), 50A5 (*BaraA*), and 57F8 (*BaraC*). These paralogous *Baramicins* are united by the presence of the IM24 domain. In the case of *BaraB*, we additionally recovered a frameshift mutation (2R_4821599_INS) causing a premature stop segregating in the DGRP leading to the loss of IM13 and IM22 relative to the *BaraA* gene structure (Fig 2B); this truncation is present in the Dmel_R6 genome assembly, but many DGRP strains encode a CDS with either a standard (e.g. DGRP38) or extended (e.g. DGRP101) IM22 domain (a DGRP *BaraB* alignment is provided in S2 Data). Moreover, in contrast to BaraA, the initial IM10-like peptide of BaraB no longer follows a furin cleavage site, and encodes a serine (RSXR) in its IM10-like motif instead of the universal proline (RPXR) of *BaraA*-like IM10 peptides across the genus. Each of these mutations prevents the secretion of classical IM10-like and IM22 peptides by *BaraB*. Finally, *BaraC* encodes only IM24 tailed by a transmembrane domain at the C terminus (TMHMM v2.0 [33]), and thus lacks both the IM10-like peptides and IM22 (Fig 2B).

### BaraB and BaraC are not immune-inducible

**Fig 3:**
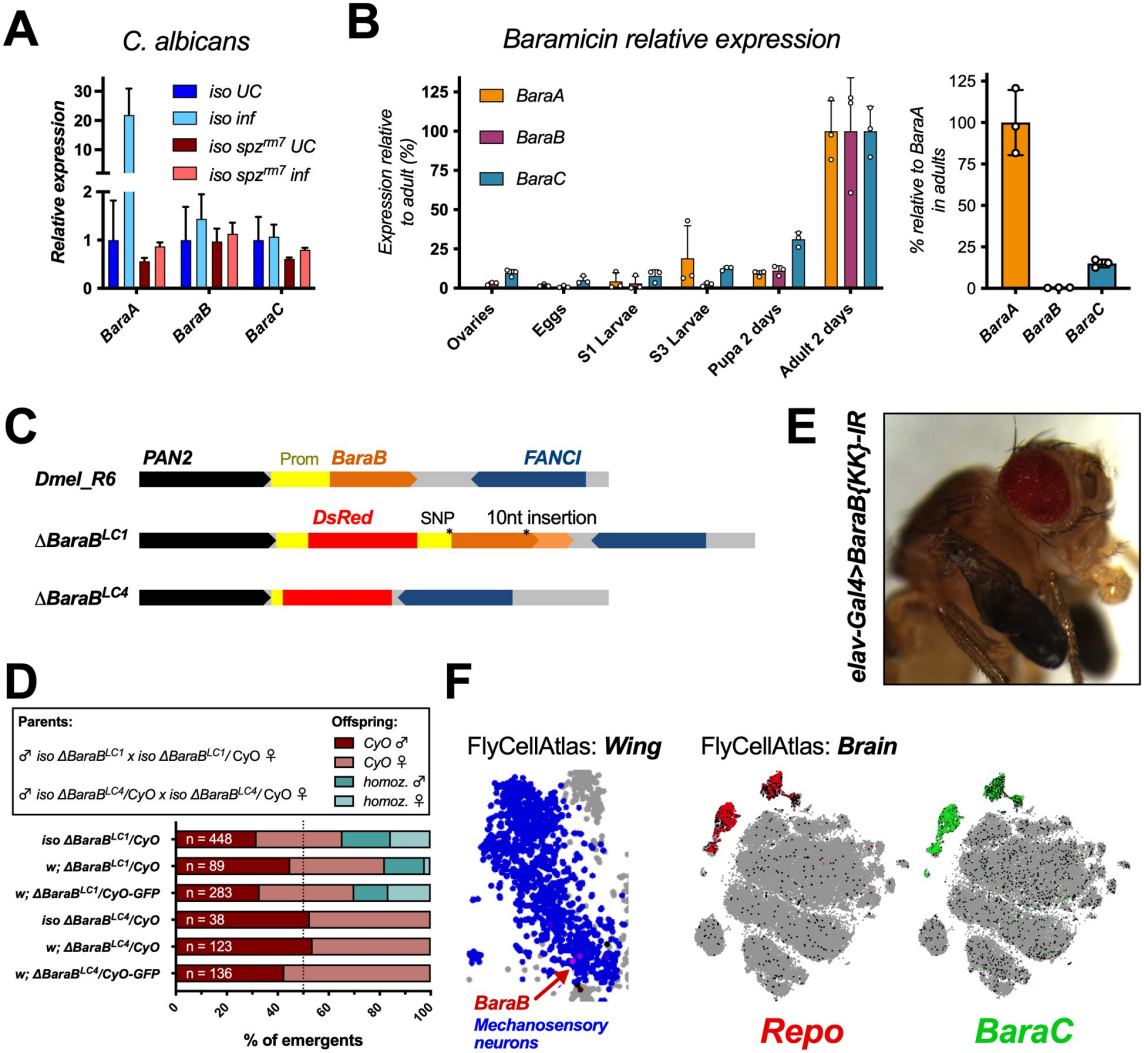
*D*. *melanogaster BaraB and BaraC* have neural functions. A) Only *BaraA* is immune-induced. *BaraB* and *BaraC* do not respond to infection, though basal *BaraC* expression relies on Toll signalling (S3C Fig). B) Time series of whole animal *Baramicin* expression over the course of development. Expression values are normalized within each gene, with expression in the adult set to 100% (left panel). For context, normalizing each gene to *BaraA* in adults shows that *BaraC* and especially *BaraB* expression is much lower at a whole fly level (right panel). C) *ΔBaraB* mutations generated in this study. *ΔBaraB*^*LC1*^ is an incidental hypomorph with reduced *BaraB* expression (S3A Fig), while *ΔBaraB*^*LC4*^ has the intended genetic replacement of *BaraB* with a DsRed cassette. Both mutations cause expression of DsRed in the eyes, ocelli, and abdomen. D) Partial lethality of *ΔBaraB*^*LC1*^ hypomorphs or complete lethality of *ΔBaraB*^*LC4*^ null flies in varied genetic backgrounds. E) The partial expansion wing phenotype is copied by *BaraB* gene silencing using the neural driver *elav-Gal4*. F) Specific wing mechanosensory neurons seem to express *BaraB* in adults. Meanwhile the *BaraC* gene almost perfectly matches the expression pattern of glia specific *Repo*-expressing cells in single cell RNA sequencing of the adult fly brain [38].

*BaraA* is strongly induced following microbial challenge (Fig 1), being predominantly regulated by the Toll pathway with a minor input from the Immune Deficiency (Imd) pathway [21,29]. We therefore assayed the expression of *BaraB* and *BaraC* in wild-type flies, and also flies with defective Toll (*spz*^*rm7*^) or Imd (*Rel*^*E20*^) signalling to see if their basal expression relied on these pathways. Surprisingly, neither gene was induced upon infection regardless of microbial challenge (Figs 3A and S2A and S2B). Of note: *BaraC* levels were consistently reduced in *spz*^*rm7*^ mutants regardless of treatment (cumulative data in S2C Fig, p = .005), suggesting *BaraC* basal expression is affected by Toll signalling. We next measured *Baramicin* expression over development from egg to adult. We found that expression of all genes increased over development and reached their highest level in young adults (Fig 3B). Of note, *BaraB* expression approached the lower limit of our assay’s detection sensitivity at early life stages. However *BaraB* was robustly detected beginning at the pupal stage, indicating it is expressed during metamorphosis. *BaraC* expression also increased markedly between the L3 larval stage and pupal stage.

Collectively we reveal that *BaraA* is part of a larger gene family. While the *BaraA* gene was first described as an immune effector, the two *Baramicin* paralogs *BaraB* and *BaraC* are not induced by infection in *D*. *melanogaster*. Both *BaraB* and *BaraC* first see increased expression during pupation, and are ultimately expressed at their highest levels in adults.

### Dmel\BaraB is required in the nervous system over the course of development

A simple interpretation of the truncated gene structure and low levels of *BaraB* expression is that this gene is undergoing pseudogenization. Indeed, AMP gene pseudogenization is common in insects including *Drosophila* [3,34,35]. To explore *BaraB* function, we used two mutations for *BaraB* (*ΔBaraB*^*LC1*^ and *ΔBaraB*^*LC4*^, generously gifted by S.A. Wasserman). These mutations were made using a CRISPR double gRNA approach to replace the *BaraB* locus with sequence from the pHD-DsRed vector. The *ΔBaraB*^*LC1*^ and *ΔBaraB*^*LC4*^ mutations differ in their ultimate effect, as *ΔBaraB*^*LC1*^ is an incidental insertion of the DsRed cassette in the promoter of the gene. This disruption reduces gene expression, resulting in a hypomorph state (S3A Fig). The *ΔBaraB*^*LC4*^ mutation however deletes the locus as intended, leading to *BaraB* null flies (Fig 3C).

We further introgressed both *ΔBaraB* mutations into the DrosDel isogenic background (referred to as *iso*) for seven generations according to Ferreira et al. [36]. At the same time, we combined the original *ΔBaraB* chromosomes with a CyO-GFP balancer chromosome in a mixed genetic background to distinguish homozygous/heterozygous larvae. In all cases, *ΔBaraB*^*LC4*^ homozygous individuals failed to develop to the adult stage, whereas homozygous *ΔBaraB*^*LC1*^ adults emerged (Fig 3D). We next assessed *BaraB* hypomorph viability by crossing *ΔBaraB*^*LC1*^ homozygous males to *ΔBaraB*^*LC1*^*/CyO* heterozygous females. The resulting offspring ratio departed from Mendelian inheritance, and was exacerbated by rearing at 29°C (S3B Fig). Using our *CyO-GFP* reporter to track hetero- vs. homozygous larvae revealed that the major lethal phase occurs primarily in the late larval and pupal stages (S3C–S3F Fig), consistent with a role for *BaraB* in the larva/pupa stage as suggested by an increase in expression at this stage (Fig 3B). Some *ΔBaraB*^*LC1*^ homozygous flies also exhibited locomotor defects, and/or a partial expansion wing phenotype (e.g. in Fig 3E) where the wings were stuck in a shrivelled state for the remainder of the fly’s lifespan. However, a proportion of *ΔBaraB*^*LC1*^ homozygotes successfully emerged, and unlike their siblings, had no immediate morphological or locomotory defects. The lifespan of morphologically normal *iso ΔBaraB*^*LC1*^ adults is nevertheless significantly shorter compared to wild-type flies and *iso ΔBaraB*^*LC1*^*/CyO* siblings (S4G Fig). We confirmed these developmental defects using ubiquitous gene silencing with *Actin5C-Gal4 (Act-Gal4)* to drive two *BaraB* RNAi/interfering RNA (IR) constructs (*TRiP-IR* and *KK-IR*). Both constructs resulted in significant lethality and occurrence of partial expansion wings (S1 Table). Genomic deficiency crosses also confirmed significantly reduced numbers of eclosing *BaraB*-deficient flies at 25°C (n = 114, χ^2^ p < .001) and 29°C (n = 63, χ^2^ p < .001) (S3H Fig). We therefore conclude that full gene deletion causes lethality at the larva-pupa transition stage, and *BaraB* hypomorphic flies suffer significant costs to fitness during development, and have reduced lifespan even following successful eclosion.

These data indicate *BaraB* is unlikely to be pseudogenized. While whole-fly *BaraB* expression is low, *BaraB* appears to be important for development. The fact that there is a bimodal outcome in hypomorph-like *ΔBaraB*^*LC1*^ adults (either severe defects or generally healthy) suggests *BaraB* could be involved in passing some checkpoint during larval/pupal development. Flies deficient for *BaraB* may be more likely to fail at this developmental checkpoint, resulting in either lethality or developmental defects.

### Baramicin B suppression in the nervous system mimics mutant phenotypes

We next sought to determine in which tissue(s) *BaraB* is required. A previous screen using neural *elav-Gal4* driven RNA interference highlighted *BaraB* silencing for lethality effects (n = 15) [37]. Given *BaraB* mutant locomotory defects, we started by silencing *BaraB* in the nervous system using the pan-neural *elav-Gal4* driver with both the *TRiP-IR* and *KK-IR BaraB-IR* lines *(IR = interfering RNA)*. We also used a combination of *UAS-Dicer2 (Dcr2)* and/or 29°C for greater silencing efficiency. In all cases, *BaraB-IR* driven by *elav-Gal4* caused a significant (p < .02) departure from Mendelian inheritance in lethality and partial expansion wing presentation (S1 Table). Moreover the frequency of both lethality and the partial expansion wing phenotype was increased with increasing strength of gene silencing, and *elav-Gal4>BaraB-IR* flies also displayed locomotion difficulties with increasing strength of gene silencing, often getting stuck in the food and moving haphazardly.

This analysis suggests that *BaraB* plays an important role in the nervous system, explaining both the lethality and partial expansion wing phenotypes. Interestingly, *BaraB* is expressed in a specific subset of mechanosensory neuron cells in the wing in FlyCellAtlas [38] (Fig 3F), despite very low levels of *BaraB* expression in other FlyCellAtlas tissue datasets. We additionally investigated the effect of *BaraB* silencing in non-neural tissues including the fat body (*c564-Gal4*), hemocytes (*hml-Gal4*), the gut (esg-Gal4, Myo1A-Gal4), the wing disc (*nubbin-Gal4*), and in myocytes (*mef2-gal4*), all of which did not present with increased lethality or partial expansion wings. We also screened neural drivers specific for glia (*Repo-Gal4*), motor neurons (*D42-*, *VGMN-*, and *OK6-Gal4*), and a *BaraA-Gal4* driver [21] that could overlap *BaraB*-expressing cells. However all these *Gal4>BaraB-IR* flies were viable and never exhibited overt morphological defects.

### Baramicin C is expressed in glia

Tissue-specific transcriptomic data indicate that *BaraC* is expressed in various neural tissues including the eye, brain, and the thoracic abdominal ganglion (S4A Fig), but also the hindgut and rectal pads pointing to a complex expression pattern [32,39]. We next searched FlyCellAtlas [38] to narrow down which neural subtypes *BaraB and BaraC* were expressed in. *BaraB* expressing cells were few, and mostly showed only low expression in this dataset. However *BaraC* was robustly expressed in all glial cell types, fully overlapping the glia marker *Repo* (Fig 3F). To confirm the observation that *BaraC* was expressed in glia, we compared the effects of *BaraC* RNA silencing (*BaraC-IR*) using *Act-Gal4* (ubiquitous), *elav-Gal4* (neural) and *Repo-Gal4* (glia) drivers on *BaraC* expression. *Act-Gal4*, *elav-Gal4*, and *Repo-Gal4* reduced *BaraC* expression to ~14%, ~63% and ~57% that of control flies (S4B Fig, overall controls vs. neural/glia-IR, p = .002). We also screened for overt lethality, and locomotor or developmental defects upon *BaraC* silencing using ubiquitous *Act-Gal4* and neural *elav-Gal4>Dcr2* or *Repo-Gal4*. However *BaraC* silencing never produced overt phenotypes in morphology or locomotor activity.

Collectively, our results support the notion that *BaraC* is expressed in the nervous system, and are consistent with *BaraC* expression being most localized to glial cells.

### Repeated genomic turnover of the Baramicin gene family

Our results thus far show that *BaraA*-like genes are consistently immune-induced in all *Drosophila* species (Fig 1), however the two paralogs *Dmel\BaraB* and *Dmel\BaraC* are not immune-induced, and are truncated in a fashion that deletes some or all of the antifungal IM10-like peptides (Fig 2B). These two *Baramicins* are now enriched in the nervous system (Fig 3E and 3F). In the case of *BaraB*, a role in the nervous system is evidenced by severe defects recapitulated using pan-neural RNA silencing. In the case of *BaraC*, nervous system expression is evidenced by a clear overlap with *Repo*-expressing cells.

**Fig 4:**
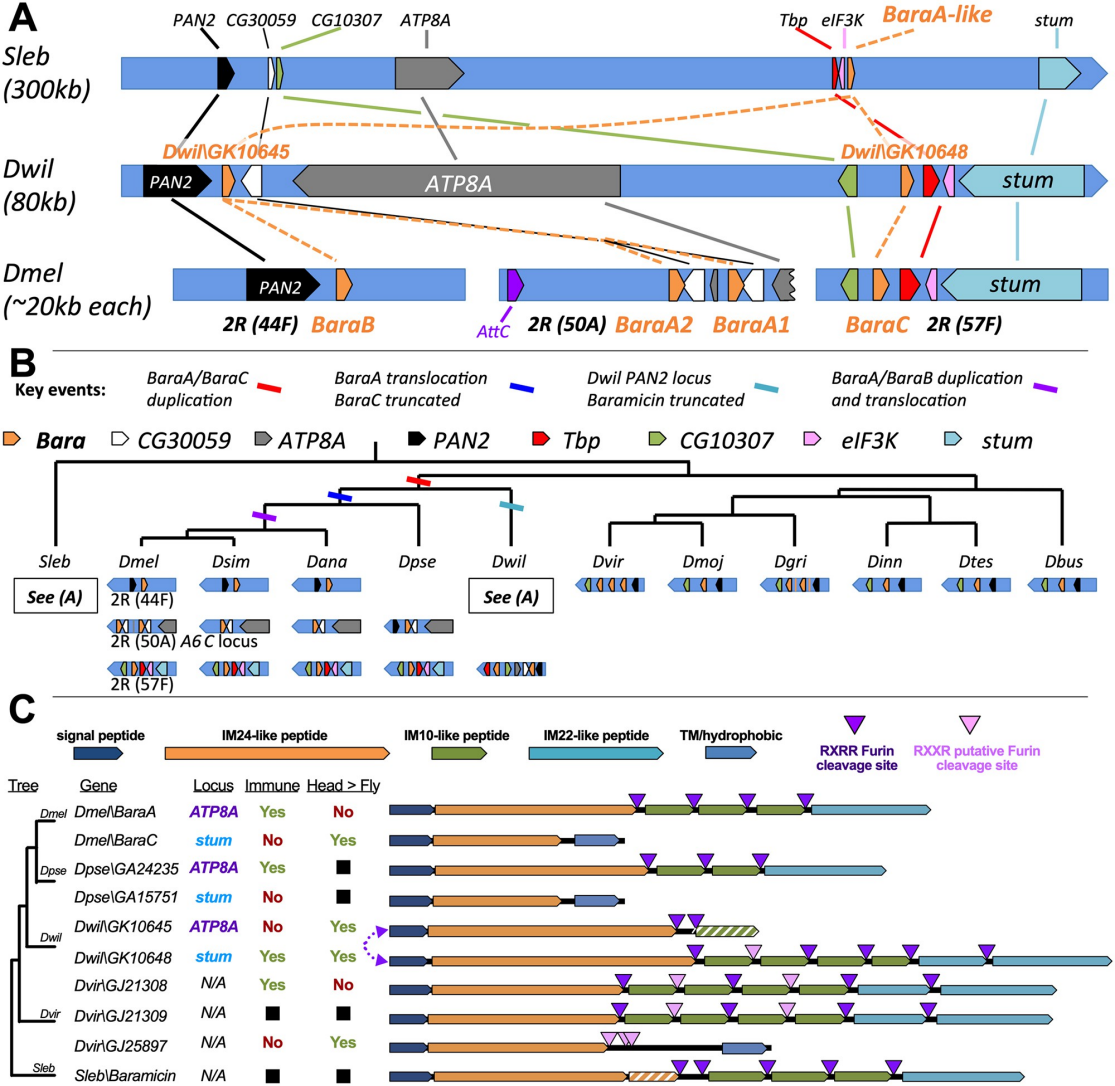
*Baramicin* evolutionary history. A) Detailed map of genomic neighbourhoods in the drosophilids *S*. *lebanonensis*, *D*. *willistoni*, and *D*. *melanogaster*, detailing inferred duplication, inversion, and translocation events. Gene names are given as found in *D*. *melanogaster*. B) Cladogram and genomic loci further detailing the series of events leading to the extant *Baramicin* loci of *Drosophila* species. Loci in *S*. *lebanonensis* and flies in the subgenus *Drosophila* encode only one *Baramicin* gene, indicating the ancestral drosophilid likely encoded only one *Baramicin*. C) IM24-specific *Baramicins* arose from convergent evolution both in gene structure and expression profile. Genomic loci are described here as *ATP8A* or *stum* reflecting prominent genes neighbouring the *Baramicins* (see Fig 4A). Expression dynamics relating to immune-induction or enrichment in the head (yes/no) are shown in Figs 2 and S5. The *Baramicin* loci in *D*. *willistoni* are syntenic with *D*. *melanogaster*, but evolved in a vice versa fashion (purple arrow). The *D*. *virilis Baramicins GJ21309* and *GJ25897* are direct sister genes (100% identity at N-terminus).

While *BaraA*-like genes are conserved throughout the genus *Drosophila*, *BaraB* is conserved only in Melanogaster group flies, and *BaraC* is found only in Melanogaster and Obscura group flies, indicating that both paralogs stem from duplication events of a *BaraA*-like ancestor (Fig 4). To determine the ancestry of each *D*. *melanogaster Baramicin* gene, we traced their evolutionary history by analyzing genomic synteny through hierarchical orthologous groups [40]. Ancestry tracing revealed that these three loci ultimately stem from a single-locus ancestor encoding only one *Baramicin* gene that resembled *Dmel\BaraA* (Fig 4A). This is evidenced by the presence of only a single *BaraA*-like gene in the outgroup *S*. *lebanonensis*, and also in multiple lineages of the subgenus Drosophila (Fig 4B). Indeed, the general *BaraA* gene structure encoding IM24, tandem repeats of IM10-like peptides, and IM22 is conserved in *S*. *lebanonensis* and all *Drosophila* species (Fig 4C). On the other hand, the *Dmel\BaraC* gene comes from an ancient duplication restricted to the subgenus Sophophora, and *Dmel\BaraB* resulted from a more recent duplication found only in the Melanogaster group (Fig 4B).

We originally found outgroup *Baramicins* by reciprocal BLAST searches, and screened *BaraA-like* genes encoding the full suite of Baramicin peptides for immune induction (i.e. encoding IM24, IM10-likes, and IM22: expression in Fig 1). However following genomic synteny analysis, we realized that the *D*. *willistoni BaraA*-like gene *Dwil\GK10648* is syntenic with the *Dmel\BaraC* locus (Fig 4A), yet this gene is immune-induced (Fig 1D) and retains a *BaraA*-like gene structure (Fig 4C). On the other hand, *Dwil\GK10645* is found at the locus syntenic with *BaraA*, but has undergone an independent truncation to encode just an IM24 peptide (similar to *Dmel\BaraC*). Thus these two *D*. *willistoni* genes have evolved similar to *D*. *melanogaster BaraA/BaraC*, but in a vice versa fashion. This suggests a pattern of convergent evolution with two key points: **i)** the duplication event producing *Dmel\BaraA* and *Dmel\BaraC* originally copied a full-length *BaraA*-like gene to the *BaraC* locus, and **ii)** the derivation of an IM24-specific gene structure has occurred more than once (*Dmel\BaraC* and *Dwil\GK10645*). Indeed, another independent IM24-specific *Baramicin* gene is present in *D*. *virilis* (*Dvir\GJ25897*), which is a direct sister of the *BaraA*-like gene *Dvir\GJ21309* (the signal peptides of these genes is identical at the nucleotide level, and see Fig 4C). Thus *Baramicins* in both *D*. *willistoni* and *D*. *virilis* have convergently evolved towards an IM24-specific protein structure resembling *Dmel\BaraC*. We checked the expression of these truncated *Baramicins* in each species upon infection. As was the case for *Dmel\BaraC*, neither gene is immune-induced (S5A–S5C Fig). Given the glial expression of *Dmel\BaraC*, we reasoned that the heads of adult flies (rich in nerve tissue) should be enriched in *BaraC* compared to whole animals. Indeed we saw a significant enrichment of *BaraC* in the heads of *D*. *melanogaster* males compared to whole flies, which was not the case for *BaraA* (S5D Fig). When we checked the heads of *D*. *willistoni* and *D*. *virilis*, we indeed saw a consistent and significant enrichment in the head for the IM24-specific genes *Dwil\GK10645* and *Dvir\GJ25897*, while *BaraA*-like genes were more variable in expression (S5E and S5F Fig).

Genomic synteny shows the gene structure and immune expression of *BaraA* are the ancestral state, and *Dmel\BaraB* and *Dmel\BaraC* are paralogs derived from independent duplication events. Strikingly, we observe a parallel evolution of expression pattern and gene structure in *Baramicins* of *D*. *willistoni* and *D*. *virilis*. Moreover these independent IM24-specific *Baramicins* across species are not immune induced, and are enriched in the head. Expression data across genes and species are shown in S5 Fig and summarized in Fig 4C.

### Residue 29 in the IM24 domain evolves in lineage-specific fashions

Thus far we have shown that IM24-specific genes are expressed in the nervous system, yet IM24 is the only peptide domain conserved across all *Baramicin* genes. We therefore wanted to better understand the properties of IM24 to know if any evolutionary patterns might distinguish the IM24 domains of nervous system-expressed genes from IM24 domains of immune-induced genes. We were unable to model the protein satisfactorily with various protein prediction techniques, preventing a 3D comprehension of the IM24 peptide. Therefore we asked if we could highlight any residues in this traditionally immune peptide that might correlate with nervous system or immune-induced gene lineages to better understand what aspect of IM24 contributes to it being retained in neural contexts.

**Fig 5:**
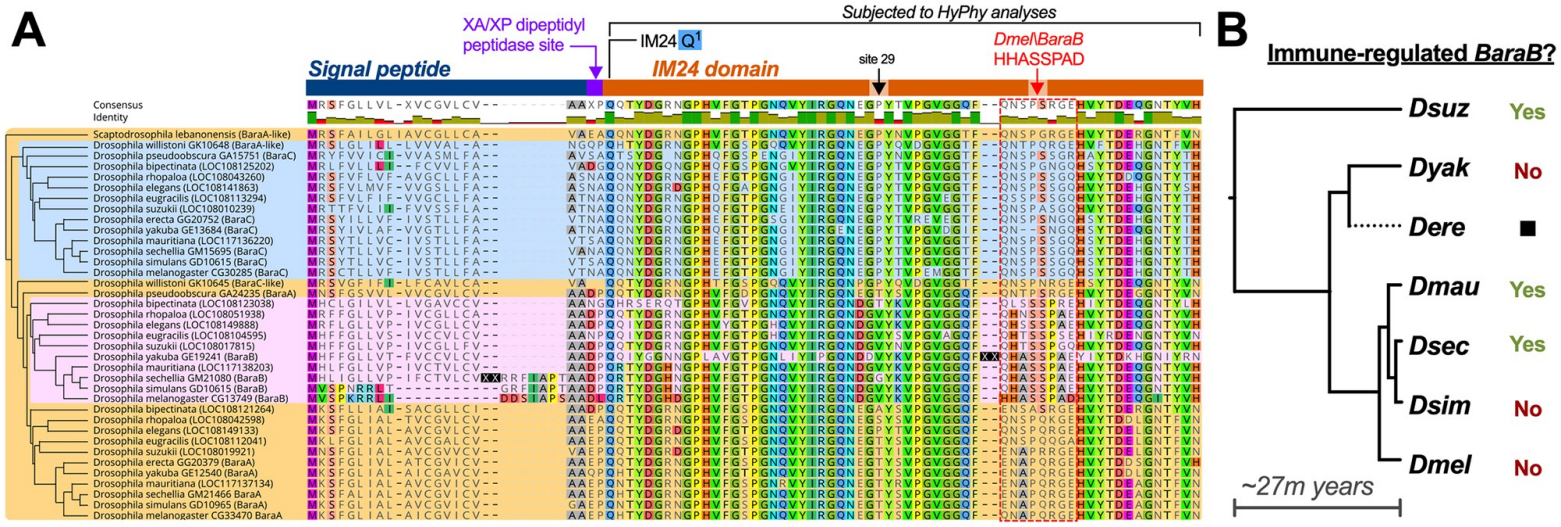
*BaraB* and IM24 rapid evolution. A) Evolution of the signal peptide and core IM24 domain. Residue highlighting indicates agreement with *Dmel\BaraB*. Arrows indicate site 29 and the *D*. *melanogaster* ^40^HHASSPAD^48^ domain. Insertion events in *D*. *yakuba* and *D*. *sechellia BaraB* are denoted as XX to save space. While longer than other Baramicin proteins, the *D*. *sechellia* signal peptide is predicted to remain functional (SignalP 5.0). The cladogram on the left shows genomic relatedness according to speciation and gene duplication patterns independent of sequence similarity. Background colouring is included to show birth of novel *Baramicin* loci/lineages. B) *BaraB* immune inducibility across the Melanogaster group phylogeny. The *D*. *erecta BaraB* gene is pseudogenized by multiple premature stop codons, and the *D*. *yakuba* gene is not immune-induced and encodes a 9-residue insertion in the IM24 peptide bordering the HHASSPAD domain (see XX site in A). However the *BaraB* genes of *D*. *suzukii*, and both *D*. *mauritiana* and *D*. *sechellia* remain inducible by infection, while the *BaraB* genes of *D*. *simulans* and *D*. *melanogaster* are not and are expressed at very low levels (S6 Fig). This pattern suggests that *BaraB* of *D*. *melanogaster* (and *D*. *simulans*) lost immune induction only recently, and is correlated with the loss of the *BaraB* signal peptide.

To do this, we screened for positive selection (elevated non-synonymous mutation rate) in the IM24 domain using the HyPhy package implemented in Datamonkey.org [41] using separate codon alignments of *Baramicin* IM24 domains beginning at their conserved Q^1^ starting residue. As is recommended with the HyPhy package [41], we employed multiple statistical approaches including Likelihood (FEL), Bayesian (FUBAR), and Count-based (SLAC) analyses to ensure patterns in selection analyses were robust to different methods of investigation. Specifically, we used locus-specific alignments (e.g. genes at the *stum* locus in Fig 4B were all analyzed together) to ensure IM24 evolution reflected locus-specific evolution. FEL, FUBAR, and SLAC site-specific analyses each suggest strong purifying selection in many residues of the IM24 domain (p-adj < .05, data in S3 Data), agreeing with the general protein structure of IM24 being broadly conserved (Fig 5A). However one residue (site 29) was consistently highlighted as evolving under positive selection using each type of statistical approach for genes located at the Sophophora *ATP8A* locus (*BaraA* genes and *Dwil\GK10645*: p-adj < .05; Fig 5A). This site is universally Proline in *Baramicin* genes located at the stum locus (*BaraC*-like), in both *D*. *willistoni Baramicins*, and in the outgroup *S*. *lebanonensis*, suggesting Proline is the ancestral state. However this residue diverges in both the *BaraA* (commonly Threonine) and *BaraB* (commonly Valine) lineages. We also note that two sites on either side of site 29 (site 27 and site 31) similarly diverge by lineage in an otherwise highly conserved region of the IM24 domain. FUBAR analysis (but not FEL or SLAC) similarly found evidence of positive selection at site 31 in the *BaraA* locus genes (p-adj = .026). Thus this neighbouring site could also be evolving in a non-random fashion. Similar analyses of the *BaraB* and *stum* loci *Baramicins* did not find evidence of site-specific positive selection.

We highlight site 29 as a key residue in IM24 that diverged in *Baramicin* in lineage-specific fashions. This ancestrally Proline residue has settled on a Threonine in most *BaraA*-like genes of Obscura and Melanogaster group flies, and a Valine in most *BaraB* genes, which are unique to the Melanogaster group. The ancestral Proline residue is found in both *D*. *willistoni Baramicins*, alongside significant enrichment of both genes in the head, despite only one gene being immune-induced (Figs 4C and S5). Thus it is unclear how this site contributes to tissue-specific *Baramicin* functions, but Threonine and Valine residues evolved in the *BaraA* and *BaraB* lineages.

### Another IM24 motif in Baramicin lineages varies through relaxed selection

Visual inspection of aligned IM24 proteins shows the overall IM24 domain is broadly conserved, except in sites 40–48 (Fig 5A). This motif aligns to residues ^40^HHASSPAD^48^ of *Dmel\BaraB*, and departs in lineage-specific fashions; the three C-terminal residues of this motif are diagnostic of each gene lineage (*BaraA*, *BaraB*, and *BaraC* have RGE, PXE, or (S/N)GQ respectively; Fig 5A). However even with additional branch-site selection analyses (aBSREL and BUSTED [42]), we found no evidence of positive selection at this motif, and in fact many residues also failed to show evidence of purifying selection. For instance, six of nine sites of this motif in the *BaraA* locus analysis failed to reach significance (p > .05) for purifying selection in SLAC analysis (S3 Data).

Given an absence of positive selection, and many residues failing to reach significance for purifying selection at the residue 40–48 motif, we suspect this motif is diversifying due to drift effects through relaxed selection. The high conservation of IM24 residues up- and downstream of this motif is nevertheless striking. One possible explanation may be that these residues act as a linker between the two functional parts of IM24 up- and downstream of residues 40–48. Perhaps supporting this interpretation, we found that *D*. *yakuba BaraB* independently lost immune induction alongside an insertion at site 40 (Fig 5). Such speculation awaits validation by robust protein modelling efforts.

### Overt structural change best explains Baramicin loss of immune induction

We found that site 29 evolves rapidly in *Baramicin* lineages, but this site is common to both immune-induced and non-induced *Baramicin* genes (e.g. in *D*. *willistoni*). Thus IM24 sequence variation does not explain why IM24-specific *Baramicins* lose immune inducibility. However within the *BaraA/BaraB* lineage, we observed that *BaraB* genes commonly encode Valine at IM24 site 29, compared to Threonine in *BaraA*. As the *BaraB* locus is derived from an ancestral immune-induced (Fig 4A), it is unclear if other *BaraB* genes are immune-inducible, and thus what IM24 evolutionary patterns (like Valine at site 29) might predict *BaraB* functional divergence.

We therefore performed infection experiments in diverse species across the Melanogaster group to see if their *BaraB* genes had similarly lost immune induction (see S6 Fig for qPCR data). Surprisingly, we found that the lack of immune inducibility of *Dmel\BaraB* is extremely recent, as Melanogaster sister species like *D*. *sechellia* and *D*. *mauritiana* encode immune inducible *BaraB* loci (summary in Fig 5B). However, we found that *D*. *simulans BaraB* lacked immune induction, despite *D*. *simulans* being most closely related to *D*. *sechellia* [43]. Thus IM24 sequence evolution does not predict immune induction.

This drew our attention to the overall protein structure of the various extant *BaraB* genes. A striking feature of the Dmel\BaraB protein is the absence of a functional signal peptide (Fig 2B). This signal peptide sequence is conserved in all *Baramicin* lineages, except in *Dmel/BaraB* and also *Dsim/BaraB*. Indeed despite *D*. *simulans* being more closely related to *D*. *sechellia* and *D*. *mauritiana*, both *Dmel\BaraB* and *Dsim\BaraB* encode a homologous N-terminus of parallel length (Fig 5A). Loss of the BaraB signal peptide is therefore more specifically associated with loss of immune expression in the Melanogaster species complex (*D*. *simulans*, *D*. *sechellia*, *D*. *mauritiana*, and *D*. *melanogaster*). The last common ancestor of *D*. *simulans*, *D*. *sechellia*, and *D*. *mauritiana* is estimated to be just ~250,000 years ago, and these species diverged from *D*. *melanogaster* ~3 million years ago [43]. The fact that *D*. *simulans* uniquely encodes this *Dmel\BaraB*-like sequence suggests it was either introgressed from one species to the other prior to the complete development of hybrid inviability, or reflects incomplete lineage sorting of this locus in the Melanogaster species complex.

Overall, we find no evidence to suggest IM24 domain sequence has evolved drastically to allow for function in the nervous system. Rather than small sequence changes, overt structural changes like truncation to focus on the IM24 domain and loss of a signal peptide in *Dmel\BaraB* are associated with *Baramicins* expressed in the nervous system. After duplication, *Baramicin* daughter lineages have repeatedly derived neural-specific expression through subfunctionalization of the IM24 domain from the overall precursor protein. Importantly, this finding suggests that the ancestral *Baramicin* encoded peptides with distinct roles in either the immune response or the nervous system.

## Discussion

Recent studies have suggested AMP genes regulate behavioural responses, and may be involved in disease progression through interactions with the nervous system. Many of these genes encode polypeptides with multiple mature products. To date, little attention has been paid to AMP genes in these neural contexts at the level of the sub-peptides they encode. Here we demonstrate that the *Baramicin* antimicrobial peptide gene of *Drosophila* ancestrally encodes distinct peptides that may interact with either the nervous system (IM24) or invading pathogens (IM10-like, IM22). These peptides are maturated from a longer precursor protein, accomplished via furin cleavage. Importantly, this suggests that AMP genes can mediate these distinct neural and immune roles via specialized sub-peptides, and not necessarily due to dual action of a single peptide. Moreover the ‘protein operon’ structure of immune-induced *Baramicins* can act as a mechanism to allow peptides with distinct roles to be produced simultaneously from a single mRNA transcript.

There is building evidence that immune-induced AMPs and AMP-like genes affect the nervous system. Loss of Metchnikowin protects flies from neurodegeneration after traumatic brain injury [44], *Induced by infection (IBIN)* regulates behavioural changes in flies after seeing parasitoid wasps [19], epidermal nematode AMPs trigger motor neuron autophagy [17] and sleep [18] after infection, and loss of *Diptericin B* produced by the fat body leads to memory deficits in *Drosophila* [15]. This last example is intriguing, as *Diptericin B* also encodes a polypeptide maturated by furin cleavage, and its effect on memory was derived from peptide secreted into the hemolymph by the fat body and not from neural expression. Similarly, we recently found that *BaraA* deletion causes infected flies to display an erect wing behavioural phenotype, which was independent of active infection, and could be rescued by priming the hemolymph with *BaraA* expressed by the fat body [21]. Thus some component of *BaraA* likely interacts with some host target(s) to prevent this behaviour during the immune response. The present study suggests this could be due to the action of BaraA IM24, given IM24 is retained in genes more specifically expressed in the nervous system. We also found no natural selection patterns in IM24 that were unique to immune or non-immune genes across the phylogeny, suggesting the core IM24 peptide does not need to drastically change its structure to suit expression in the nervous system. However AMPs and neuropeptides have many similar features, including cationic charge and amphipathicity [26]. Thus while our results suggest that IM24 of different *Baramicin* genes might underlie *Baramicin* interactions with the nervous system, we cannot exclude the possibility that IM24 is also antimicrobial, or even that antimicrobial activity is IM24’s ancestral purpose. Future studies could use tagged IM24 transgenes or synthetic peptides to determine the host binding partner(s) of secreted IM24 from the immune-induced *Dmel\BaraA*, and/or to see if IM24 binds to microbial membranes.

One human AMP recently implicated in chronic neuroinflammatory disease is the Cathelicidin LL-37 [22,23,45]. Like *Baramicin*, the *Cathelicidin* gene family is unified by its N-terminal domain: the “Cathelin” domain. However to date no one has described antimicrobial activity of the Cathelin domain in vitro [1]. Instead, *Cathelicidin* research has focused almost exclusively on the mature peptide LL-37 at the C-terminus of mammalian *Cathelicidin* genes. Reflecting on *Baramicin* evolution and the implication of *Cathelicidin* in neurodegenerative diseases, what does the Cathelin domain do? While this study was conducted in fruit flies, we hope we have emphasized the importance of considering each peptide of AMP genes for in vivo function. This is relevant to neural processes even if the gene is typically thought of for its role in innate immunity. Indeed, recent studies of *Drosophila* AMPs have emphasized that in vitro activity does not always predict the interactions that arise from endogenous loss of function study [14,46]. Care should be taken not to conflate in vitro activity with realized in vivo function. Most studies focus on AMPs specifically in an immune role, but this is akin to ‘looking for your keys under the streetlight.’ To understand AMP functions in vivo, genetic approaches will be necessary that allow a more global view of gene function.

In summary, we reflect on the structural characteristics of AMP genes through the lens of the *Baramicins*. We found that one sub-peptide of the immune-induced *Baramicin* ancestor is readily adapted for functions relating to the nervous system. Meanwhile, other sub-peptides known to suppress fungi are repeatedly lost in daughter genes that lack immune inducibility, suggesting they are irrelevant to neural functions. As AMP genes commonly encode polypeptides maturated by furin cleavage (including *Baramicin*), it will be interesting to consider the functions of AMP genes in neural processes not simply at the level of the gene, but at the level of the mature peptides produced by that gene. This consideration may explain how some polypeptide immune effectors play dual roles in disparate contexts.

## Materials and methods

### DGRP population screening and bioinformatics analyses

Genomic sequence data were downloaded from GenBank default reference assemblies and Kim et al. [47], and DGRP sequence data from http://dgrp2.gnets.ncsu.edu/ [31]. Sequence comparisons and alignment figures were prepared using Geneious R10 [48], Prism 7, and Inkscape. Alignments were performed using MUSCLE or MAFFT followed by manual curation, and phylogenetic analyses were performed to validate sequence patterns using the Neighbour Joining, PhyML, RaxML, and MrBayes plugins in Geneious. *BaraA* copy number screening was performed using primers specific to the duplication and *CG30059* control primers for DNA extraction (S1 Data). We found a significant correlation between *BaraA* PCR status and variant sites starting at 2R_9293471_SNP and extending to 2R_9293576_SNP (Pearson’s correlation matrix: 0.0001 < p-value < 0.005 at all nine sites), however the status of genetic variants at this site is poorly resolved and so we cannot be confident that our ~14% estimate for the *BaraA* duplication in the DGRP would hold true if long-read sequencing was employed. DGRP annotation of the *BaraA* locus in S1 Fig was generated using the UCSC *D*. *melanogaster* DGRP2 genome browser. Selection analyses were performed using the HyPhy package implemented in datamonkey.org [41]. Codon alignments of the IM24 domain used in Fig 5A are included as a .fasta file in S3 Data alongside outputs from FEL, FUBAR, SLAC, and aBSREL selection analyses.

### Fly genetics

The *BaraB*^*LC1*^ and *BaraB*^*LC4*^ mutations were generated using CRISPR with two gRNAs and an HDR vector by cloning 5’ and 3’ region-homologous arms into the pHD-DsRed vector, and consequently *ΔBaraB* flies express DsRed in their eyes, ocelli, and abdomen. The following PAM sites were used for CRISPR bordering the *BaraB* region. Slashes indicate the cut site: 5’: GCGGGCAACAGATGTGTTCA/GGG 3’: GTCCATTGCTTATTCAAAAA/TGG. These mutants were generated in the laboratory of Steve Wasserman by Lianne Cohen, who graciously allowed their use in this study. All fly stocks including Gal4 and RNAi lines are listed in S4 Data. Experiments were performed at 25°C unless otherwise indicated. When possible, genetic crosses of 6–8 males and 6–8 females were performed in both directions to test for an effect of the X or Y chromosomes on *BaraB*-mediated lethality; crosses in both directions yielded similar results in all cases and reported data are pooled results. Fly diet consisted of a nutrient-rich lab standard food: 3.72g agar, 35.28g cornmeal, 35.28g yeast, 36mL grape juice, 2.9mL propionic acid, 15.9mL moldex, and H_2_O to 600mL.

### Infection experiments

Bacteria and yeast were grown to mid-log phase shaking at 200rpm in their respective growth media (LB, BHI, or YPG) and temperature conditions, and then pelleted by centrifugation to concentrate microbes. Resulting cultures were diluted to OD = 200 at 600nm before infections to measure gene expression. The following microbes were grown at 37°C: *Escherichia coli strain 1106* (LB) and *Candida albicans* (YPG). *Micrococcus luteus* was grown at 29°C in LB. For Figs 1 and S2, pooled fly samples were collected either 6 hours post-infection (*E*. *coli*) or 24 hours post-infection (*C*. *albicans*, *M*. *luteus*) prior to RNA extraction on pools of 5 adult males. These timepoints correspond to the maximal expression inputs of the Imd (6hpi) or Toll (24hpi) NF-κB signalling pathways, which are most specifically induced by Gram-negative bacteria (Imd) or Gram-positive bacteria or fungi (Toll) [49]. Flies were pricked in the thorax as described in [14].

RNA extractions were performed using TRIzol, Ambion DNAse treatment, and PrimeScript RT according to manufacturer’s protocols. RT-qPCR was performed using PowerUP SYBR Green master mix with primers listed in S5 Data. Gene expression differences were analyzed using the PFAFFL method [50]. For gene expression experiments requiring dissection of heads, pools of 20 males were used for either whole flies or heads dissected in ice-cold PBS and transferred immediately to a tube kept on dry ice.

### Selection analysis using the HyPhy package

Codon aligned nexus tree files were generated using either the Neighbour-joining (1000 bootstraps) or PhyML (100 bootstraps) methods including proteins beyond those shown in Fig 5. These tree files were analyzed using the HyPhy package with only 174nt pertaining to just the IM24 domain codons included. The cladogram in Fig 5A is manually drawn from known species divergences [47]. Use of either tree building method was chosen for convenience to best reflect known lineage sorting, as use of just 174nt was too information-poor to resolve exact phylogenetic relatedness reliably. Tree files were qualitatively screened to ensure topologies broadly matched known species sortings, and thus ensure only relevant comparisons were made given the genomic synteny analysis in Fig 4 is principally informative of true gene lineages. HyPhy analyses were run separately for each *Baramicin* lineage within their clade, defined by genomic synteny; i.e. based on locus (e.g. ATP8A locus), and not considering convergent gene structures. We used three site-specific analyses (FEL, FUBAR, and SLAC) that use three independent statistical approaches (Likelihood, Bayesian, and Count-based methods respectively). We also employed both BUSTED and aBSREL branch-site analyses, which are likelihood methods that differ in their approach of testing whole-phylogeny selection or branch-specific comparisons respectively; an anology might be performing analysis of variance (ANOVA) at the level of the entire ANOVA, or comparing multiple groups against each other and subsequently using multiple test correction. Each tree was rooted using the *Scaptodrosophila lebanonensis Baramicin* as an outgroup with ancestral characteristics; we did not include *Baramicins* of the subgenus Drosophila as including these resulted in long-branch attraction of the Willistoni group *Baramicins* to subgenus Drosophila lineages, which would confound relevant phylogenetic comparisons. When applicable, all internal branches were assessed for potential selection. For *Baramicins* of the *ATP8A* locus, one site (site 29) was highlighted as experiencing positive selection using FEL, FUBAR, and SLAC analyses (p-adj = .011, .013, and .039 respectively). Additionally, site 31 was also highlighted by FUBAR (p-adj = .026), but not FEL or SLAC analyses (p-adj > .05). BUSTED analysis also supported diversifying selection in the *BaraA* lineage (ATP8A locus, LRT p-adj = .008), indicating at least one site on at least one test branch has experienced diversifying selection within the ATP8A lineage. The aBSREL branch-site analysis specifically highlights the branch distinguishing the Willistoni group *Baramicins* from the other Sophophora species (p-adj = .0045), suggesting variation between these branches drives the signals of diversifying selection in the BUSTED analysis. This result is intuitive, as we find a parallel but opposite evolution of Baramicin protein structure in *Baramicins* of the *ATP8A* locus in *D*. *willistoni* compared with *Baramicins* of other Sophophora species. Furthermore, in whole-gene phylogenies, both *D*. *willistoni Baramicins* cluster together, supporting the notion that these two daughter genes have evolved independent from the selection that shaped the orthologues of *Dmel\BaraA* and *Dmel\BaraC*, also seen in qPCR data that showed both genes were significantly enriched in the head (S6 Fig). This phylogenetic clustering of the two *D*. *willistoni Baramicins* holds true when additional *Baramicins* from recently sequenced genomes of the Willistoni group are included (from [47] in S3 Data), indicating this is characteristic of the Willistoni group lineage and not specific to *D*. *willistoni*.

## Supporting information

S1 Fig The *BaraA* locus is poorly resolved in DGRP genome assemblies. The *BaraA1* and *BaraA2* gene regions are totally devoid of mapped variants (dashed boxes). We speculate this is due to an artefact during genomic assembly, where reads mapping equally to the two identical *BaraA* genes were discarded as non-specific. This would explain why *BaraA* is typically discarded in RNAseq datasets using such measures in their pipeline, but not in microarray data from De Gregorio et al. [51] where it is called “IM10”.
(EPS)

S2 Fig Additional assays of Baramicin expression upon infection. **A)** Neither *BaraB* nor *BaraC* are regulated by the Imd pathway, which is specifically stimulated by *E*. *coli* infection. **B)** Neither *BaraB* nor *BaraC* are induced after infection by *M*. *luteus*. **C)** *BaraC* levels were consistently depressed in *spz*^*rm7*^ flies in the unchallenged condition (UC) or upon infection with *C*. *albicans* (Fig 3B) or *M*. *luteus* (S4B Fig). Data here are pooled for *iso* wild type or *iso spz*^*rm7*^ flies without regard for infection treatment (student’s t, p = .005).
(EPS)

S3 Fig *BaraB* mutation is highly deleterious, even in *ΔBaraB*^*LC1*^ hypomorphs. **A)** Diagram of *BaraB* mutant loci and qPCR showing that *ΔBaraB*^*LC1*^ is a hypomorph mutation. Under our normal qPCR assay conditions, *BaraB* expression is not detected in *ΔBaraB*^*LC1*^ homozygotes. However using highly concentrated cDNA beyond our assay’s valid range (100ng/10μL reaction), we could detect *BaraB* transcript in *ΔBaraB*^*LC1*^ flies. Quantification shown here is intended only to show that *BaraB* transcript can be recovered from *ΔBaraB*^*LC1*^ homozygotes, and to give a sense of relative whole-fly expression levels. **B)** Emergent frequencies of *ΔBaraB*^*LC1*^ flies at different temperatures. **C)** Aborted pupae (yellow arrows) are a common occurrence in *ΔBaraB* vials, and sometimes contain fully-developed adults that simply never eclosed. In **D-F**: ns = not significant, * = p < .05, *** p = < .001. **D)** The ratio of *ΔBaraB*^*LC1*^/CyO-GFP to *ΔBaraB*^*LC1*^ homozygous larvae drops between the S2 and S3 larval stages (χ^2^, p = .515 and p = .012 respectively). **E)** Frequency of successfully eclosing adults using *ΔBaraB*^*LC4*^/CyO-GFP flies. **F)** Frequency of successfully eclosing adults using *ΔBaraB*^*LC1*^/CyO-GFP flies. **G)** *BaraB* mutation negatively affects lifespan. *iso ΔBaraB*^*LC1*^ homozygotes suffer reduced lifespan even relative to their *iso ΔBaraB*^*LC1*^*/CyO* siblings. By comparison, *iso ΔBaraA* flies that used the same vector for mutant generation live as wild-type. *ATM^8^* flies suffer precocious neurodegeneration and are included as short-lived controls [52]. **H)** *ΔBaraB*^*LC1*^ crossed to the genomic deficiency line (*Df(9063)*) supports a partial-lethal effect of *BaraB* mutation.
(EPS)

S4 Fig *BaraC* is expressed in the nervous system, but also the hindgut and rectal pads. **A)** FlyAtlas2 expression data for *BaraC*. **B)** RT-qPCR of *BaraC* in whole flies using different Gal4 drivers to express *BaraC* RNAi. *BaraC* is knocked down by both the *elav-Gal4* and *Repo-Gal4* nervous system drivers. Cumulatively, nervous system drivers significantly depress *BaraC* expression compared to *BaraC-IR* controls (student’s t, p < .01). Ubiquitous knockdown using *Act>BaraC-IR* provides a comparative knockdown to better understand the strength of nervous system-specific knockdowns at the whole fly level.
(EPS)

S5 Fig RT-qPCR of *Baramicin* genes in diverse species. **A-C)** Independent IM24-specific genes in *D*. *melanogaster* (A), *D*. *willistoni* (B), and *D*. *virilis* (C) are not induced by infection. *BomBc3* is included as an immune-induced control. **D-F)** The independent IM24-specific genes (blue) of *D*. *melanogaster* (D), *D*. *willistoni* (E), and *D*. *virilis* (F) are each enriched in the head relative to whole flies. *BaraA-like* genes (orange) were expressed more stochastically in the head, but also generally showed an enrichment pattern relative to whole flies (not always significant). Each data point represents an independent pooled sample from 20 male flies. Data were analyzed using one-way ANOVA with Holm’s-Sidak multiple test correction.
(EPS)

S6 Fig The *D*. *melanogaster BaraB* gene acquired its non-immune role only recently. **A)** Cladogram of the Melanogaster species group. The presence of a functional signal peptide (Fig 5A), and the disruption of the *D*. *yakuba* IM24 peptide by an in-frame insertion is noted. A summary of whether *BaraB* is an immune-induced orthologue (B-G) is annotated. **B-G)** *Baramicin* expression data from Melanogaster group flies either unchallenged or infected with *M*. *luteus*. *BaraB* is immune-induced in *D*. *suzukii*, *D*. *sechellia*, and *D*. *mauritiana*, but not in *D*. *simulans* and *D*. *melanogaster*, which both lack signal peptide structures. *Drosophila yakuba BaraB* is not immune-induced (C), has an insertion event in its IM24 peptide (Fig 5A), and its sister species *D*. *erecta* has pseudogenized its *BaraB* orthologue (Fig 5B), suggesting pseudogenization may explain the lack of immune induction in *D*. *yakuba BaraB*.
(EPS)

S1 Table *BaraB* RNAi summary statistics. Crosses used either the TRiP or KK *BaraB-IR* lines, driven by either *Actin5C-Gal4* or *elav-Gal4*, sometimes including *UAS-Dcr2*. Rearing at 29°C and inclusion of *UAS-Dcr2* increases the strength of RNA silencing. In the event there was no lethality, it was expected that emerging *elav>TRiP-IR* flies would follow simple mendelian inheritance. However both *elav>TRiP-IR* and *elav>Dcr2*, *TRiP-IR* resulted in partial lethality and occasional partial expansion wings (χ^2^ p < .02). Crosses using *KK-IR* used homozygous flies, and so we did not assess lethality using mendelian inheritance. However using this construct, no adults emerged when *elav>Dcr2*, *KK-IR* flies were reared at 29°C. Rare emergents (N = 11 after three experiments) occurred at 25°C, all of which bore partial expansion wings. Using *elav-Gal4* at 29°C without *Dcr2*, we observed greater numbers of emerging adults, but 100% of flies had partial expansion wings. Finally, *elav>KK-IR* flies at 25°C suffered both partial lethality and partial expansion wings, but normal-winged flies began emerging (χ^2^ p < .001).
(PDF)

S1 Data *BaraA* duplication status within the DGRP, with the caveat that our PCR assay may be sensitive to cryptic variation in the unresolved DGRP loci. (XLSX)

S2 Data Alignment of *BaraB* gene sequences from the DGRP showing the two variants either truncating or extending the *BaraB* coding sequence. (RTF)

S3 Data Ouputs from HyPhy analyses, with nexus tree files used in analyses and text file summaries of cumulative analyses per locus. (ZIP)

S4 Data Fly stocks used in this study. (XLSX)

S5 Data PCR primers used in this study. (XLSX)

## References

[1] Zanetti M. The role of cathelicidins in the innate host defenses of mammals. Curr Issues Mol Biol. 2005;7: 179–196. doi: 10.21775/cimb.007.179 16053249

[2] Hanson MA, Lemaitre B. New insights on Drosophila antimicrobial peptide function in host defense and beyond. Curr Opin Immunol. 2020;62: 22–30. doi: 10.1016/j.coi.2019.11.008 31835066

[3] Hanson MA, Lemaitre B, Unckless RL. Dynamic Evolution of Antimicrobial Peptides Underscores Trade-Offs Between Immunity and Ecological Fitness. Frontiers in Immunology. 2019;10: 2620. doi: 10.3389/fimmu.2019.02620 31781114 PMC6857651

[4] Sackton TB, Lazzaro BP, Clark AG, Wittkopp P. Rapid expansion of immune-related gene families in the house fly, musca domestica. Molecular Biology and Evolution. 2017. doi: 10.1093/molbev/msw285 28087775 PMC5400391

[5] Vilcinskas A, Mukherjee K, Vogel H. Expansion of the antimicrobial peptide repertoire in the invasive ladybird Harmonia axyridis. Proceedings of the Royal Society B: Biological Sciences. 2013. doi: 10.1098/rspb.2012.2113 23173204 PMC3574431

[6] Wang Y, Zhu S. The defensin gene family expansion in the tick Ixodes scapularis. Developmental and Comparative Immunology. 2011. doi: 10.1016/j.dci.2011.03.030 21540051

[7] Halldórsdóttir K, Árnason E. Trans-species polymorphism at antimicrobial innate immunity cathelicidin genes of Atlantic cod and related species. PeerJ. 2015;3: e976. doi: 10.7717/peerj.976 26038731 PMC4451034

[8] Hellgren O, Sheldon BC, Buckling A. In vitro tests of natural allelic variation of innate immune genes (avian beta-defensins) reveal functional differences in microbial inhibition. Journal of Evolutionary Biology. 2010;23: 2726–2730. doi: 10.1111/j.1420-9101.2010.02115.x 21121085

[9] Chapman JR, Hill T, Unckless RL. Balancing selection drives maintenance of genetic variation in Drosophila antimicrobial peptides. Genome Biology and Evolution. 2019;11: 2691–2701. doi: 10.1093/gbe/evz191 31504505 PMC6764478

[10] Jiggins FM, Kim KW. A screen for immunity genes evolving under positive selection in Drosophila. Journal of Evolutionary Biology. 2007;20: 965–970. doi: 10.1111/j.1420-9101.2007.01305.x 17465907

[11] Tennessen JA. Molecular evolution of animal antimicrobial peptides: Widespread moderate positive selection. 2005. doi: 10.1111/j.1420-9101.2005.00925.x 16313451

[12] Hanson MA, Hamilton PT, Perlman SJ. Immune genes and divergent antimicrobial peptides in flies of the subgenus Drosophila. BMC evolutionary biology. 2016;16: 228. doi: 10.1186/s12862-016-0805-y 27776480 PMC5078906

[13] Unckless RL, Howick VM, Lazzaro BP. Convergent Balancing Selection on an Antimicrobial Peptide in Drosophila. Current Biology. 2016;26: 257–262. doi: 10.1016/j.cub.2015.11.063 26776733 PMC4729654

[14] Hanson MA, Dostálová A, Ceroni C, Poidevin M, Kondo S, Lemaitre B. Synergy and remarkable specificity of antimicrobial peptides in vivo using a systematic knockout approach. eLife. 2019;8. doi: 10.7554/elife.44341 30803481 PMC6398976

[15] Barajas-azpeleta R, Wu J, Gill J, Welte R. Antimicrobial peptides modulate long-term memory. PLoS Genetics. 2018; 1–26. doi: 10.1371/journal.pgen.1007440 30312294 PMC6224176

[16] Toda H, Williams JA, Gulledge M. A sleep-inducing gene, nemuri, links sleep and immune function in Drosophila. Science. 2019;515: 509–515. doi: 10.1126/science.aat1650 30705188 PMC6505470

[17] Lezi E, Zhou T, Koh S, Chuang M, Sharma R, Pujol N, et al. An Antimicrobial Peptide and Its Neuronal Receptor Regulate Dendrite Degeneration in Aging and Infection. Neuron. 2018;97: 125–138.e5. doi: 10.1016/j.neuron.2017.12.001 29301098 PMC5757245

[18] Singanayagam A, Glanville N, Cuthbertson L, Bartlett NW, Finney LJ, Turek E, et al. Inhaled corticosteroid suppression of cathelicidin drives dysbiosis and bacterial infection in chronic obstructive pulmonary disease. Science Translational Medicine. 2019. doi: 10.1126/scitranslmed.aav3879 31462509 PMC7237237

[19] Ebrahim SAM, Talross GJS, Carlson JR. Sight of parasitoid wasps accelerates sexual behavior and upregulates a micropeptide gene in Drosophila. Nat Commun. 2021;12: 2453. doi: 10.1038/s41467-021-22712-0 33907186 PMC8079388

[20] Kobler JM, Rodriguez Jimenez FJ, Petcu I, Grunwald Kadow IC. Immune Receptor Signaling and the Mushroom Body Mediate Post-ingestion Pathogen Avoidance. Curr Biol. 2020;30: 4693–4709.e3. doi: 10.1016/j.cub.2020.09.022 33007248

[21] Hanson MA, Cohen LB, Marra A, Iatsenko I, Wasserman SA, Lemaitre B. The Drosophila Baramicin polypeptide gene protects against fungal infection. PLoS Pathog. 2021;17: e1009846. doi: 10.1371/journal.ppat.1009846 34432851 PMC8423362

[22] De Lorenzi E, Chiari M, Colombo R, Cretich M, Sola L, Vanna R, et al. Evidence that the human innate immune peptide LL-37 may be a binding partner of amyloid-β and inhibitor of fibril assembly. Journal of Alzheimer’s Disease. 2017;59: 1213–1226. doi: 10.3233/JAD-170223 28731438 PMC5611894

[23] Lee M, Shi X, Barron AE, McGeer E, McGeer PL. Human antimicrobial peptide LL-37 induces glial-mediated neuroinflammation. Biochemical Pharmacology. 2015;94: 130–141. doi: 10.1016/j.bcp.2015.02.003 25686659

[24] Dominy SS, Lynch C, Ermini F, Benedyk M, Marczyk A, Konradi A, et al. Porphyromonas gingivalis in Alzheimer’s disease brains: Evidence for disease causation and treatment with small-molecule inhibitors. Science Advances. 2019;5. doi: 10.1126/sciadv.aau3333 30746447 PMC6357742

[25] Abbott A. Are infections seeding some cases of Alzheimer’s disease? Nature. 2020;587: 22–25. doi: 10.1038/d41586-020-03084-9 33149296

[26] Brogden KA, Guthmiller JM, Salzet M, Zasloff M. The nervous system and innate immunity: The neuropeptide connection. Nat Immunol. 2005;6. doi: 10.1038/ni1209 15908937

[27] Gerdol M, Schmitt P, Venier P, Rocha G, Rosa RD, Destoumieux-Garzón D. Functional Insights From the Evolutionary Diversification of Big Defensins. Front Immunol. 2020;11: 758. doi: 10.3389/fimmu.2020.00758 32425943 PMC7203481

[28] Casteels-Josson K, Capaci T, Casteels P, Tempst P. Apidaecin multipeptide precursor structure: a putative mechanism for amplification of the insect antibacterial response. The EMBO journal. 1993;12: 1569–78. doi: 10.1002/j.1460-2075.1993.tb05801.x 8467807 PMC413370

[29] Huang J, Lou Y, Liu J, Bulet P, Jiao R, Hoffmann JA, et al. The BaramicinA gene is required at several steps of the host defense against Enterococcus faecalis and Metarhizium robertsii in a septic wound infection model in Drosophila melanogaster. bioRxiv; 2020 Nov. doi: 10.1101/2020.11.23.394809

[30] Suvorov A, Kim BY, Wang J, Armstrong EE, Peede D, D’Agostino ERR, et al. Widespread introgression across a phylogeny of 155 Drosophila genomes. Current Biology. 2021; S0960982221014962. doi: 10.1016/j.cub.2021.10.052 34788634 PMC8752469

[31] Mackay TFC, Richards S, Stone EA, Barbadilla A, Ayroles JF, Zhu D, et al. The Drosophila melanogaster Genetic Reference Panel. Nature. 2012;482: 173–8. doi: 10.1038/nature10811 22318601 PMC3683990

[32] Leader DP, Krause SA, Pandit A, Davies SA, Dow JAT. FlyAtlas 2: A new version of the Drosophila melanogaster expression atlas with RNA-Seq, miRNA-Seq and sex-specific data. Nucleic Acids Research. 2018;46: D809–D815. doi: 10.1093/nar/gkx976 29069479 PMC5753349

[33] Krogh A, Larsson B, von Heijne G, Sonnhammer EL. Predicting transmembrane protein topology with a hidden Markov model: application to complete genomes. J Mol Biol. 2001;305: 567–580. doi: 10.1006/jmbi.2000.4315 11152613

[34] Quesada H, Ramos-Onsins SE, Aguade M. Birth-and-death evolution of the Cecropin multigene family in Drosophila. Journal of Molecular Evolution. 2005;60: 1–11. doi: 10.1007/s00239-004-0053-4 15696364

[35] Rolff J, Schmid-Hempel P. Perspectives on the evolutionary ecology of arthropod antimicrobial peptides. Philosophical Transactions of the Royal Society B: Biological Sciences. 2016;371. doi: 10.1098/rstb.2015.0297 27160599 PMC4874394

[36] Ferreira ÁG, Naylor H, Esteves SS, Pais IS, Martins NE, Teixeira L. The Toll-dorsal pathway is required for resistance to viral oral infection in Drosophila. PLoS Pathog. 2014;10: e1004507. doi: 10.1371/journal.ppat.1004507 25473839 PMC4256459

[37] Neely GG, Hess A, Costigan M, Keene AC, Goulas S, Langeslag M, et al. A Genome-wide Drosophila screen for heat nociception identifies α2δ3 as an evolutionarily conserved pain gene. Cell. 2010;143: 628–638. doi: 10.1016/j.cell.2010.09.047 21074052 PMC3040441

[38] Li H, Janssens J, De Waegeneer M, Kolluru SS, Davie K, Gardeux V, et al. Fly Cell Atlas: a single-cell transcriptomic atlas of the adult fruit fly. Genomics; 2021 Jul. doi: 10.1101/2021.07.04.451050 PMC8944923 35239393

[39] Hammonds AS, Bristow CA, Fisher WW, Weiszmann R, Wu S, Hartenstein V, et al. Spatial expression of transcription factors in Drosophila embryonic organ development. Genome Biol. 2013;14: R140. doi: 10.1186/gb-2013-14-12-r140 24359758 PMC4053779

[40] Train C-M, Pignatelli M, Altenhoff A, Dessimoz C. iHam and pyHam: visualizing and processing hierarchical orthologous groups. Bioinformatics (Oxford, England). 2019;35: 2504–2506. doi: 10.1093/bioinformatics/bty994 30508066 PMC6612847

[41] Delport W, Poon AFY, Frost SDW, Kosakovsky Pond SL. Datamonkey 2010: A suite of phylogenetic analysis tools for evolutionary biology. Bioinformatics. 2010;26: 2455–2457. doi: 10.1093/bioinformatics/btq429 20671151 PMC2944195

[42] Murrell B, Weaver S, Smith MD, Wertheim JO, Murrell S, Aylward A, et al. Gene-wide identification of episodic selection. Mol Biol Evol. 2015;32: 1365–1371. doi: 10.1093/molbev/msv035 25701167 PMC4408417

[43] Chakraborty M, Chang C-H, Khost DE, Vedanayagam J, Adrion JR, Liao Y, et al. Evolution of genome structure in the Drosophila simulans species complex. Genome Res. 2021;31: 380–396. doi: 10.1101/gr.263442.120 33563718 PMC7919458

[44] Swanson LC, Rimkus SA, Ganetzky B, Wassarman DA. Loss of the Antimicrobial Peptide Metchnikowin Protects Against Traumatic Brain Injury Outcomes in Drosophila melanogaster. G3 (Bethesda). 2020;10: 3109–3119. doi: 10.1534/g3.120.401377 32631949 PMC7466987

[45] Moir RD, Lathe R, Tanzi RE. The antimicrobial protection hypothesis of Alzheimer’s disease. Alzheimer’s & Dementia. 2018;14: 1602–1614. doi: 10.1016/j.jalz.2018.06.3040 30314800

[46] Clemmons AW, Lindsay SA, Wasserman SA. An Effector Peptide Family Required for Drosophila Toll-Mediated Immunity. PLoS Pathogens. 2015;11. doi: 10.1371/journal.ppat.1004876 25915418 PMC4411088

[47] Kim BY, Wang J, Miller DE, Barmina O, Delaney EK, Thompson A, et al. Highly contiguous assemblies of 101 drosophilid genomes. eLife. 2021;10: e66405. doi: 10.7554/eLife.66405 34279216 PMC8337076

[48] Kearse M, Moir R, Wilson A, Stones-Havas S, Cheung M, Sturrock S, et al. Geneious Basic: An integrated and extendable desktop software platform for the organization and analysis of sequence data. Bioinformatics. 2012. doi: 10.1093/bioinformatics/bts199 22543367 PMC3371832

[49] Lemaitre B, Reichhart JM, Hoffmann JA. Drosophila host defense: differential induction of antimicrobial peptide genes after infection by various classes of microorganisms. Proceedings of the National Academy of Sciences of the United States of America. 1997;94: 14614–9. doi: 10.1073/pnas.94.26.14614 9405661 PMC25070

[50] Pfaffl MW. A new mathematical model for relative quantification in real-time RT-PCR. Nucleic Acids Res. 2001;29: e45. doi: 10.1093/nar/29.9.e45 11328886 PMC55695

[51] De Gregorio E, Spellman PT, Tzou P, Rubin GM, Lemaitre B. The Toll and Imd pathways are the major regulators of the immune response in Drosophila. EMBO Journal. 2002;21: 2568–2579. doi: 10.1093/emboj/21.11.2568 12032070 PMC126042

[52] Petersen AJ, Katzenberger RJ, Wassarman DA. The innate immune response transcription factor relish is necessary for neurodegeneration in a Drosophila model of ataxia-telangiectasia. Genetics. 2013;194: 133–142. doi: 10.1534/genetics.113.150854 23502677 PMC3632461

