# Supplementary material for: Repeated truncation of a modular antimicrobial peptide gene for neural context
Source: PLoS Genet. 2022 Jun 17;18(6):e1010259. doi: 10.1371/journal.pgen.1010259 (PMC9246212; doi:10.1371/journal.pgen.1010259)
Supplement: S1 Table — Crosses used either the TRiP or KK BaraB-IR lines, driven by either Actin5C-Gal4 or elav-Gal4, sometimes including UAS-Dcr2. Rearing at 29°C and inclusion of UAS-Dcr2 increases the strength of RNA silencing. In the event there was no lethality, it was expected that emerging elav>TRiP-IR flies would follow simple mendelian inheritance. However both elav>TRiP-IR and elav>Dcr2, TRiP-IR resulted in partial lethality and occasional partial expansion wings (χ2 p < .02). Crosses using KK-IR used homozygous flies, and so we did not assess lethality using mendelian inheritance. However using this construct, no adults emerged when elav>Dcr2, KK-IR flies were reared at 29°C. Rare emergents (N = 11 after three experiments) occurred at 25°C, all of which bore partial expansion wings. Using elav-Gal4 at 29°C without Dcr2, we observed greater numbers of emerging adults, but 100% of flies had partial expansion wings. Finally, elav>KK-IR flies at 25°C suffered both partial lethality and partial expansion wings, but normal-winged flies began emerging (χ2 p < .001). (PDF) [file pgen.1010259.s007.pdf]

Parents and temperature	Offspring	Sex	# eclosed	χ^2	p-value
TRiP					
y1 v1; P{TRiP.HMJ23624}attP40/CyO	Act-Gal4 / CyO	m	26	25.687	**p < .001**
Act-Gal4/CyO-GFP ; +		f	30		
25°C	TRiP / CyO	m	26		
		f	29		
	Act-Gal4 > BaraB-IR{TRiP}	m	2		
		f	18		
y1 v1; P{TRiP.HMJ23624}attP40/CyO male	elav>Dcr2 ; +/CyO	m	67	10.037	**p < .02**
elav>Dcr2 ;; female		f	62		
25°C	elav>Dcr2 ; +/BaraB-IR	m	38		
		f	47		
y1 v1; P{TRiP.HMJ23624}attP40/CyO male	elav-Gal4; +/CyO	m	29	13.593	**p < .01**
elav-Gal4> ;; female		f	46		
25°C	elav-Gal4 ; +/BaraB-IR	m	21		
		f	22		
KK					
P{KK112854}VIE-260B	Act-Gal4 ; +/CyO	m	27	45.187	**p < .001**
Act-Gal4/CyO-GFP ; +		f	55		
25°C	Act-Gal4 > BaraB-IR{KK}	m	14		
		f	11		
P{KK112854}VIE-260B male	elav>Dcr2 ; +/BaraB-IR	m	0	11.000	**p < .001**
elav>Dcr2 ;; female		f	11		
25°C					
P{KK112854}VIE-260B male	normal wing	m	0	87.766	**p < .001**
elav-Gal4> ;; female		f	0		
29°C	partial expansion wing	m	28		
		f	57		
P{KK112854}VIE-260B male	normal wing	m	37	32.133	**p < .001**
elav-Gal4> ;; female		f	47		
25°C	partial expansion wing	m	31		
		f	5		
