# Supplementary material for: Repeated truncation of a modular antimicrobial peptide gene for neural context
Source: PLoS Genet. 2022 Jun 17;18(6):e1010259. doi: 10.1371/journal.pgen.1010259 (PMC9246212; doi:10.1371/journal.pgen.1010259)
Supplement: S2 Data — (RTF) [file pgen.1010259.s009.rtf]

>Dmel_R6ATGGTCTCGCCCAAACGGAGACTAATCGACGATTCCATTGCTCCCTCAGCTGCCGATCTCCAGCGGACATACGACGGACATGATGGGCCTCACGTTTTCGGAACACCCGGCAATCAAGTCTACATTCGGGGGCAGAACGATGGCGTATACAAGGTGCCAGGAGTAGGTGGTCAGTTCCATCACGCATCGTCGCCAGCAGATCACGTCTATACGGATGAGCAGGGGATCACGTATGTGCACAAAAAAGACGCTGGAGGACCAGGAACACATACGCTTAGAGGACCAGGTCTAGCTGCTGTACAGCCCGCCTACTCCACTGTGCAGCCTGCGGGACTTCGCAATCCCCAGTTTCATGTGGAGCGAAGTGGTCGCACTGTGGATGTTGGTTCTGGAGGATTTGTCGTCCAAAGGAGCAGACGTAGTCCTCAGTTCCATGTGGAGCGTCCTGGTCGCACTGTGGATGTGGGTTCCGGAGGATTTTTTGTCCAAAGGGGCAGGCGTAGTCCCCAGTTTCCTGTGGAGCGACCTTGGCAAAG----------GGCTTTGAAGGAC>line_21ATGGTCTCGCCCAAACGGAGACTAATCGACGATTCCATTGCTCCCTCAGCTGCCGATCTCCAGCGGACATACGACGGACATGATGGGCCTCACGTTTTCGGAACACCCGGCAATCAAGTCTACATTCGGGGGCAGAACGATGGCGTATACAAGGTGCCAGGAGTAGGTGGTCAGTTCCATCACGCATCGTCGCCAGCAGATCACGTCTATACGGATGAGCAGGGGATCACGTATGTGCACAAAAAAGACGCTGGAGGACCAGGAACACATACGCTTAGAGGACCAGGTCTAGCTGCTGTACAGCCCGCCTACTCCACTGTGCAGCCTGCGGGACTTCGCAATCCCCAGTTTCATGTGGAGCGAAGTGGTCGCACTGTGGATGTTGGTTCTGGAGGATTTGTCGTCCAAAGGAGCAGACGTAGTCCTCAGTTCCATGTGGAGCGTCCTGGTCGCACTGTGGATGTGGGTTCCGGAGGATTTTTTGTCCAAAGGGGCAGGCGTAGTCCCCAGTTTCCTGTGGAGCGACCTTGGCAAAG----------GGCTTTGAAGGAC>line_32ATGGTCTCGCCCAAACGGAGACTAATCGACGATTCCATTGCTCCCTCAGCTGCCGATCTCCAGCGGACATACGACGGACATGATGGGCCTCACGTTTTCGGAACACCCGGCAATCAAGTCTACATTCGGGGGCAGAACGATGGCGTATACAAGGTGCCAGGAGTAGGTGGTCAGTTCCATCACGCATCGTCGCCAGCAGATCACGTCTATACGGATGAGCAGGGGATCACGTATGTGCACAAAAAAGACGCTGGAGGACCAGGAACACATACGCTTAGAGGACCAGGTCTAGCTGCTGTACAGCCCGCCTACTCCACTGTGCAGCCTGCGGGACTTCGCAATCCCCAGTTTCATGTGGAGCGAAGTGGTCGCACTGTGGATGTTGGTTCTGGAGGATTTGTCGTCCAAAGGAGCAGACGTAGTCCTCAGTTCCATGTGGAGCGTCCTGGTCGCACTGTGGATGTGGGTTCCGGAGGATTTTTTGTCCAAAGGGGCAGGCGTAGTCCCCAGTTTCCTGTGGAGCGACCTTGGCAAAG----------GGCTTTGAAGGAC>line_40ATGGTCTCGCCCAAACGGAGACTAATCGACGATTCCATTGCTCCCTCAGCTGCCGATCTCCAGCGGACATACGACGGACATGATGGGCCTCACGTTTTCGGAACACCCGGCAATCAAGTCTACATTCGGGGGCAGAACGATGGCGTATACAAGGTGCCAGGAGTAGGTGGTCAGTTCCATCACGCATCGTCGCCAGCAGATCACGTCTATACGGATGAGCAGGGGATCACGTATGTGCACAAAAAAGACGCTGGAGGACCAGGAACACATACGCTTAGAGGACCAGGTCTAGCTGCTGTACAGCCCGCCTACTCCACTGTGCAGCCTGCGGGACTTCGCAATCCCCAGTTTCATGTGGAGCGAAGTGGTCGCACTGTGGATGTTGGTTCTGGAGGATTTGTCGTCCAAAGGAGCAGACGTAGTCCTCAGTTCCATGTGGAGCGTCCTGGTCGCACTGTGGATGTGGGTTCCGGAGGATTTTTTGTCCAAAGGGGCAGGCGTAGTCCCCAGTTTCCTGTGGAGCGACCTTGGCAAAG----------GGCTTTGAAGGAC>line_138ATGGTCTCGCCCAAACGGAGACTAATCGACGATTCCATTGCTCCCTCAGCTGCCGATCTCCAGCGGACATACGACGGACATGATGGGCCTCACGTTTTCGGAACACCCGGCAATCAAGTCTACATTCGGGGGCAGAACGATGGCGTATACAAGGTGCCAGGAGTAGGTGGTCAGTTCCATCACGCATCGTCGCCAGCAGATCACGTCTATACGGATGAGCAGGGGATCACGTATGTGCACAAAAAAGACGCTGGAGGACCAGGAACACATACGCTTAGAGGACCAGGTCTAGCTGCTGTACAGCCCGCCTACTCCACTGTGCAGCCTGCGGGACTTCGCAATCCCCAGTTTCATGTGGAGCGAAGTGGTCGCACTGTGGATGTTGGTTCTGGAGGATTTGTCGTCCAAAGGAGCAGACGTAGTCCTCAGTTCCATGTGGAGCGTCCTGGTCGCACTGTGGATGTGGGTTCCGGAGGATTTTTTGTCCAAAGGGGCAGGCGTAGTCCCCAGTTTCCTGTGGAGCGACCTTGGCAAAG----------GGCTTTGAAGGAC>line_195ATGGTCTCGCCCAAACGGAGACTAATCGACGATTCCATTGCTCCCTCAGCTGCCGATCTCCAGCGGACATACGACGGACATGATGGGCCTCACGTTTTCGGAACACCCGGCAATCAAGTCTACATTCGGGGGCAGAACGATGGCGTATACAAGGTGCCAGGAGTAGGTGGTCAGTTCCATCACGCATCGTCGCCAGCAGATCACGTCTATACGGATGAGCAGGGGATCACGTATGTGCACAAAAAAGACGCTGGAGGACCAGGAACACATACGCTTAGAGGACCAGGTCTAGCTGCTGTACAGCCCGCCTACTCCACTGTGCAGCCTGCGGGACTTCGCAATCCCCAGTTTCATGTGGAGCGAAGTGGTCGCACTGTGGATGTTGGTTCTGGAGGATTTGTCGTCCAAAGGAGCAGACGTAGTCCTCAGTTCCATGTGGAGCGTCCTGGTCGCACTGTGGATGTGGGTTCCGGAGGATTTTTTGTCCAAAGGGGCAGGCGTAGTCCCCAGTTTCCTGTGGAGCGACCTTGGCAAAG----------GGCTTTGAAGGAC>line_217ATGGTCTCGCCCAAACGGAGACTAATCGACGATTCCATTGCTCCCTCAGCTGCCGATCTCCAGCGGACATACGACGGACATGATGGGCCTCACGTTTTCGGAACACCCGGCAATCAAGTCTACATTCGGGGGCAGAACGATGGCGTATACAAGGTGCCAGGAGTAGGTGGTCAGTTCCATCACGCATCGTCGCCAGCAGATCACGTCTATACGGATGAGCAGGGGATCACGTATGTGCACAAAAAAGACGCTGGAGGACCAGGAACACATACGCTTAGAGGACCAGGTCTAGCTGCTGTACAGCCCGCCTACTCCACTGTGCAGCCTGCGGGACTTCGCAATCCCCAGTTTCATGTGGAGCGAAGTGGTCGCACTGTGGATGTTGGTTCTGGAGGATTTGTCGTCCAAAGGAGCAGACGTAGTCCTCAGTTCCATGTGGAGCGTCCTGGTCGCACTGTGGATGTGGGTTCCGGAGGATTTTTTGTCCAAAGGGGCAGGCGTAGTCCCCAGTTTCCTGTGGAGCGACCTTGGCAAAG----------GGCTTTGAAGGAC>line_239ATGGTCTCGCCCAAACGGAGACTAATCGACGATTCCATTGCTCCCTCAGCTGCCGATCTCCAGCGGACATACGACGGACATGATGGGCCTCACGTTTTCGGAACACCCGGCAATCAAGTCTACATTCGGGGGCAGAACGATGGCGTATACAAGGTGCCAGGAGTAGGTGGTCAGTTCCATCACGCATCGTCGCCAGCAGATCACGTCTATACGGATGAGCAGGGGATCACGTATGTGCACAAAAAAGACGCTGGAGGACCAGGAACACATACGCTTAGAGGACCAGGTCTAGCTGCTGTACAGCCCGCCTACTCCACTGTGCAGCCTGCGGGACTTCGCAATCCCCAGTTTCATGTGGAGCGAAGTGGTCGCACTGTGGATGTTGGTTCTGGAGGATTTGTCGTCCAAAGGAGCAGACGTAGTCCTCAGTTCCATGTGGAGCGTCCTGGTCGCACTGTGGATGTGGGTTCCGGAGGATTTTTTGTCCAAAGGGGCAGGCGTAGTCCCCAGTTTCCTGTGGAGCGACCTTGGCAAAG----------GGCTTTGAAGGAC>line_306ATGGTCTCGCCCAAACGGAGACTAATCGACGATTCCATTGCTCCCTCAGCTGCCGATCTCCAGCGGACATACGACGGACATGATGGGCCTCACGTTTTCGGAACACCCGGCAATCAAGTCTACATTCGGGGGCAGAACGATGGCGTATACAAGGTGCCAGGAGTAGGTGGTCAGTTCCATCACGCATCGTCGCCAGCAGATCACGTCTATACGGATGAGCAGGGGATCACGTATGTGCACAAAAAAGACGCTGGAGGACCAGGAACACATACGCTTAGAGGACCAGGTCTAGCTGCTGTACAGCCCGCCTACTCCACTGTGCAGCCTGCGGGACTTCGCAATCCCCAGTTTCATGTGGAGCGAAGTGGTCGCACTGTGGATGTTGGTTCTGGAGGATTTGTCGTCCAAAGGAGCAGACGTAGTCCTCAGTTCCATGTGGAGCGTCCTGGTCGCACTGTGGATGTGGGTTCCGGAGGATTTTTTGTCCAAAGGGGCAGGCGTAGTCCCCAGTTTCCTGTGGAGCGACCTTGGCAAAG----------GGCTTTGAAGGAC>line_320ATGGTCTCGCCCAAACGGAGACTAATCGACGATTCCATTGCTCCCTCAGCTGCCGATCTCCAGCGGACATACGACGGACATGATGGGCCTCACGTTTTCGGAACACCCGGCAATCAAGTCTACATTCGGGGGCAGAACGATGGCGTATACAAGGTGCCAGGAGTAGGTGGTCAGTTCCATCACGCATCGTCGCCAGCAGATCACGTCTATACGGATGAGCAGGGGATCACGTATGTGCACAAAAAAGACGCTGGAGGACCAGGAACACATACGCTTAGAGGACCAGGTCTAGCTGCTGTACAGCCCGCCTACTCCACTGTGCAGCCTGCGGGACTTCGCAATCCCCAGTTTCATGTGGAGCGAAGTGGTCGCACTGTGGATGTTGGTTCTGGAGGATTTGTCGTCCAAAGGAGCAGACGTAGTCCTCAGTTCCATGTGGAGCGTCCTGGTCGCACTGTGGATGTGGGTTCCGGAGGATTTTTTGTCCAAAGGGGCAGGCGTAGTCCCCAGTTTCCTGTGGAGCGACCTTGGCAAAG----------GGCTTTGAAGGAC>line_321ATGGTCTCGCCCAAACGGAGACTAATCGACGATTCCATTGCTCCCTCAGCTGCCGATCTCCAGCGGACATACGACGGACATGATGGGCCTCACGTTTTCGGAACACCCGGCAATCAAGTCTACATTCGGGGGCAGAACGATGGCGTATACAAGGTGCCAGGAGTAGGTGGTCAGTTCCATCACGCATCGTCGCCAGCAGATCACGTCTATACGGATGAGCAGGGGATCACGTATGTGCACAAAAAAGACGCTGGAGGACCAGGAACACATACGCTTAGAGGACCAGGTCTAGCTGCTGTACAGCCCGCCTACTCCACTGTGCAGCCTGCGGGACTTCGCAATCCCCAGTTTCATGTGGAGCGAAGTGGTCGCACTGTGGATGTTGGTTCTGGAGGATTTGTCGTCCAAAGGAGCAGACGTAGTCCTCAGTTCCATGTGGAGCGTCCTGGTCGCACTGTGGATGTGGGTTCCGGAGGATTTTTTGTCCAAAGGGGCAGGCGTAGTCCCCAGTTTCCTGTGGAGCGACCTTGGCAAAG----------GGCTTTGAAGGAC>line_357ATGGTCTCGCCCAAACGGAGACTAATCGACGATTCCATTGCTCCCTCAGCTGCCGATCTCCAGCGGACATACGACGGACATGATGGGCCTCACGTTTTCGGAACACCCGGCAATCAAGTCTACATTCGGGGGCAGAACGATGGCGTATACAAGGTGCCAGGAGTAGGTGGTCAGTTCCATCACGCATCGTCGCCAGCAGATCACGTCTATACGGATGAGCAGGGGATCACGTATGTGCACAAAAAAGACGCTGGAGGACCAGGAACACATACGCTTAGAGGACCAGGTCTAGCTGCTGTACAGCCCGCCTACTCCACTGTGCAGCCTGCGGGACTTCGCAATCCCCAGTTTCATGTGGAGCGAAGTGGTCGCACTGTGGATGTTGGTTCTGGAGGATTTGTCGTCCAAAGGAGCAGACGTAGTCCTCAGTTCCATGTGGAGCGTCCTGGTCGCACTGTGGATGTGGGTTCCGGAGGATTTTTTGTCCAAAGGGGCAGGCGTAGTCCCCAGTTTCCTGTGGAGCGACCTTGGCAAAG----------GGCTTTGAAGGAC>line_371ATGGTCTCGCCCAAACGGAGACTAATCGACGATTCCATTGCTCCCTCAGCTGCCGATCTCCAGCGGACATACGACGGACATGATGGGCCTCACGTTTTCGGAACACCCGGCAATCAAGTCTACATTCGGGGGCAGAACGATGGCGTATACAAGGTGCCAGGAGTAGGTGGTCAGTTCCATCACGCATCGTCGCCAGCAGATCACGTCTATACGGATGAGCAGGGGATCACGTATGTGCACAAAAAAGACGCTGGAGGACCAGGAACACATACGCTTAGAGGACCAGGTCTAGCTGCTGTACAGCCCGCCTACTCCACTGTGCAGCCTGCGGGACTTCGCAATCCCCAGTTTCATGTGGAGCGAAGTGGTCGCACTGTGGATGTTGGTTCTGGAGGATTTGTCGTCCAAAGGAGCAGACGTAGTCCTCAGTTCCATGTGGAGCGTCCTGGTCGCACTGTGGATGTGGGTTCCGGAGGATTTTTTGTCCAAAGGGGCAGGCGTAGTCCCCAGTTTCCTGTGGAGCGACCTTGGCAAAG----------GGCTTTGAAGGAC>line_375ATGGTCTCGCCCAAACGGAGACTAATCGACGATTCCATTGCTCCCTCAGCTGCCGATCTCCAGCGGACATACGACGGACATGATGGGCCTCACGTTTTCGGAACACCCGGCAATCAAGTCTACATTCGGGGGCAGAACGATGGCGTATACAAGGTGCCAGGAGTAGGTGGTCAGTTCCATCACGCATCGTCGCCAGCAGATCACGTCTATACGGATGAGCAGGGGATCACGTATGTGCACAAAAAAGACGCTGGAGGACCAGGAACACATACGCTTAGAGGACCAGGTCTAGCTGCTGTACAGCCCGCCTACTCCACTGTGCAGCCTGCGGGACTTCGCAATCCCCAGTTTCATGTGGAGCGAAGTGGTCGCACTGTGGATGTTGGTTCTGGAGGATTTGTCGTCCAAAGGAGCAGACGTAGTCCTCAGTTCCATGTGGAGCGTCCTGGTCGCACTGTGGATGTGGGTTCCGGAGGATTTTTTGTCCAAAGGGGCAGGCGTAGTCCCCAGTTTCCTGTGGAGCGACCTTGGCAAAG----------GGCTTTGAAGGAC>line_383ATGGTCTCGCCCAAACGGAGACTAATCGACGATTCCATTGCTCCCTCAGCTGCCGATCTCCAGCGGACATACGACGGACATGATGGGCCTCACGTTTTCGGAACACCCGGCAATCAAGTCTACATTCGGGGGCAGAACGATGGCGTATACAAGGTGCCAGGAGTAGGTGGTCAGTTCCATCACGCATCGTCGCCAGCAGATCACGTCTATACGGATGAGCAGGGGATCACGTATGTGCACAAAAAAGACGCTGGAGGACCAGGAACACATACGCTTAGAGGACCAGGTCTAGCTGCTGTACAGCCCGCCTACTCCACTGTGCAGCCTGCGGGACTTCGCAATCCCCAGTTTCATGTGGAGCGAAGTGGTCGCACTGTGGATGTTGGTTCTGGAGGATTTGTCGTCCAAAGGAGCAGACGTAGTCCTCAGTTCCATGTGGAGCGTCCTGGTCGCACTGTGGATGTGGGTTCCGGAGGATTTTTTGTCCAAAGGGGCAGGCGTAGTCCCCAGTTTCCTGTGGAGCGACCTTGGCAAAG----------GGCTTTGAAGGAC>line_392ATGGTCTCGCCCAAACGGAGACTAATCGACGATTCCATTGCTCCCTCAGCTGCCGATCTCCAGCGGACATACGACGGACATGATGGGCCTCACGTTTTCGGAACACCCGGCAATCAAGTCTACATTCGGGGGCAGAACGATGGCGTATACAAGGTGCCAGGAGTAGGTGGTCAGTTCCATCACGCATCGTCGCCAGCAGATCACGTCTATACGGATGAGCAGGGGATCACGTATGTGCACAAAAAAGACGCTGGAGGACCAGGAACACATACGCTTAGAGGACCAGGTCTAGCTGCTGTACAGCCCGCCTACTCCACTGTGCAGCCTGCGGGACTTCGCAATCCCCAGTTTCATGTGGAGCGAAGTGGTCGCACTGTGGATGTTGGTTCTGGAGGATTTGTCGTCCAAAGGAGCAGACGTAGTCCTCAGTTCCATGTGGAGCGTCCTGGTCGCACTGTGGATGTGGGTTCCGGAGGATTTTTTGTCCAAAGGGGCAGGCGTAGTCCCCAGTTTCCTGTGGAGCGACCTTGGCAAAG----------GGCTTTGAAGGAC>line_395ATGGTCTCGCCCAAACGGAGACTAATCGACGATTCCATTGCTCCCTCAGCTGCCGATCTCCAGCGGACATACGACGGACATGATGGGCCTCACGTTTTCGGAACACCCGGCAATCAAGTCTACATTCGGGGGCAGAACGATGGCGTATACAAGGTGCCAGGAGTAGGTGGTCAGTTCCATCACGCATCGTCGCCAGCAGATCACGTCTATACGGATGAGCAGGGGATCACGTATGTGCACAAAAAAGACGCTGGAGGACCAGGAACACATACGCTTAGAGGACCAGGTCTAGCTGCTGTACAGCCCGCCTACTCCACTGTGCAGCCTGCGGGACTTCGCAATCCCCAGTTTCATGTGGAGCGAAGTGGTCGCACTGTGGATGTTGGTTCTGGAGGATTTGTCGTCCAAAGGAGCAGACGTAGTCCTCAGTTCCATGTGGAGCGTCCTGGTCGCACTGTGGATGTGGGTTCCGGAGGATTTTTTGTCCAAAGGGGCAGGCGTAGTCCCCAGTTTCCTGTGGAGCGACCTTGGCAAAG----------GGCTTTGAAGGAC>line_397ATGGTCTCGCCCAAACGGAGACTAATCGACGATTCCATTGCTCCCTCAGCTGCCGATCTCCAGCGGACATACGACGGACATGATGGGCCTCACGTTTTCGGAACACCCGGCAATCAAGTCTACATTCGGGGGCAGAACGATGGCGTATACAAGGTGCCAGGAGTAGGTGGTCAGTTCCATCACGCATCGTCGCCAGCAGATCACGTCTATACGGATGAGCAGGGGATCACGTATGTGCACAAAAAAGACGCTGGAGGACCAGGAACACATACGCTTAGAGGACCAGGTCTAGCTGCTGTACAGCCCGCCTACTCCACTGTGCAGCCTGCGGGACTTCGCAATCCCCAGTTTCATGTGGAGCGAAGTGGTCGCACTGTGGATGTTGGTTCTGGAGGATTTGTCGTCCAAAGGAGCAGACGTAGTCCTCAGTTCCATGTGGAGCGTCCTGGTCGCACTGTGGATGTGGGTTCCGGAGGATTTTTTGTCCAAAGGGGCAGGCGTAGTCCCCAGTTTCCTGTGGAGCGACCTTGGCAAAG----------GGCTTTGAAGGAC>line_399ATGGTCTCGCCCAAACGGAGACTAATCGACGATTCCATTGCTCCCTCAGCTGCCGATCTCCAGCGGACATACGACGGACATGATGGGCCTCACGTTTTCGGAACACCCGGCAATCAAGTCTACATTCGGGGGCAGAACGATGGCGTATACAAGGTGCCAGGAGTAGGTGGTCAGTTCCATCACGCATCGTCGCCAGCAGATCACGTCTATACGGATGAGCAGGGGATCACGTATGTGCACAAAAAAGACGCTGGAGGACCAGGAACACATACGCTTAGAGGACCAGGTCTAGCTGCTGTACAGCCCGCCTACTCCACTGTGCAGCCTGCGGGACTTCGCAATCCCCAGTTTCATGTGGAGCGAAGTGGTCGCACTGTGGATGTTGGTTCTGGAGGATTTGTCGTCCAAAGGAGCAGACGTAGTCCTCAGTTCCATGTGGAGCGTCCTGGTCGCACTGTGGATGTGGGTTCCGGAGGATTTTTTGTCCAAAGGGGCAGGCGTAGTCCCCAGTTTCCTGTGGAGCGACCTTGGCAAAG----------GGCTTTGAAGGAC>line_405ATGGTCTCGCCCAAACGGAGACTAATCGACGATTCCATTGCTCCCTCAGCTGCCGATCTCCAGCGGACATACGACGGACATGATGGGCCTCACGTTTTCGGAACACCCGGCAATCAAGTCTACATTCGGGGGCAGAACGATGGCGTATACAAGGTGCCAGGAGTAGGTGGTCAGTTCCATCACGCATCGTCGCCAGCAGATCACGTCTATACGGATGAGCAGGGGATCACGTATGTGCACAAAAAAGACGCTGGAGGACCAGGAACACATACGCTTAGAGGACCAGGTCTAGCTGCTGTACAGCCCGCCTACTCCACTGTGCAGCCTGCGGGACTTCGCAATCCCCAGTTTCATGTGGAGCGAAGTGGTCGCACTGTGGATGTTGGTTCTGGAGGATTTGTCGTCCAAAGGAGCAGACGTAGTCCTCAGTTCCATGTGGAGCGTCCTGGTCGCACTGTGGATGTGGGTTCCGGAGGATTTTTTGTCCAAAGGGGCAGGCGTAGTCCCCAGTTTCCTGTGGAGCGACCTTGGCAAAG----------GGCTTTGAAGGAC>line_437ATGGTCTCGCCCAAACGGAGACTAATCGACGATTCCATTGCTCCCTCAGCTGCCGATCTCCAGCGGACATACGACGGACATGATGGGCCTCACGTTTTCGGAACACCCGGCAATCAAGTCTACATTCGGGGGCAGAACGATGGCGTATACAAGGTGCCAGGAGTAGGTGGTCAGTTCCATCACGCATCGTCGCCAGCAGATCACGTCTATACGGATGAGCAGGGGATCACGTATGTGCACAAAAAAGACGCTGGAGGACCAGGAACACATACGCTTAGAGGACCAGGTCTAGCTGCTGTACAGCCCGCCTACTCCACTGTGCAGCCTGCGGGACTTCGCAATCCCCAGTTTCATGTGGAGCGAAGTGGTCGCACTGTGGATGTTGGTTCTGGAGGATTTGTCGTCCAAAGGAGCAGACGTAGTCCTCAGTTCCATGTGGAGCGTCCTGGTCGCACTGTGGATGTGGGTTCCGGAGGATTTTTTGTCCAAAGGGGCAGGCGTAGTCCCCAGTTTCCTGTGGAGCGACCTTGGCAAAG----------GGCTTTGAAGGAC>line_486ATGGTCTCGCCCAAACGGAGACTAATCGACGATTCCATTGCTCCCTCAGCTGCCGATCTCCAGCGGACATACGACGGACATGATGGGCCTCACGTTTTCGGAACACCCGGCAATCAAGTCTACATTCGGGGGCAGAACGATGGCGTATACAAGGTGCCAGGAGTAGGTGGTCAGTTCCATCACGCATCGTCGCCAGCAGATCACGTCTATACGGATGAGCAGGGGATCACGTATGTGCACAAAAAAGACGCTGGAGGACCAGGAACACATACGCTTAGAGGACCAGGTCTAGCTGCTGTACAGCCCGCCTACTCCACTGTGCAGCCTGCGGGACTTCGCAATCCCCAGTTTCATGTGGAGCGAAGTGGTCGCACTGTGGATGTTGGTTCTGGAGGATTTGTCGTCCAAAGGAGCAGACGTAGTCCTCAGTTCCATGTGGAGCGTCCTGGTCGCACTGTGGATGTGGGTTCCGGAGGATTTTTTGTCCAAAGGGGCAGGCGTAGTCCCCAGTTTCCTGTGGAGCGACCTTGGCAAAG----------GGCTTTGAAGGAC>line_509ATGGTCTCGCCCAAACGGAGACTAATCGACGATTCCATTGCTCCCTCAGCTGCCGATCTCCAGCGGACATACGACGGACATGATGGGCCTCACGTTTTCGGAACACCCGGCAATCAAGTCTACATTCGGGGGCAGAACGATGGCGTATACAAGGTGCCAGGAGTAGGTGGTCAGTTCCATCACGCATCGTCGCCAGCAGATCACGTCTATACGGATGAGCAGGGGATCACGTATGTGCACAAAAAAGACGCTGGAGGACCAGGAACACATACGCTTAGAGGACCAGGTCTAGCTGCTGTACAGCCCGCCTACTCCACTGTGCAGCCTGCGGGACTTCGCAATCCCCAGTTTCATGTGGAGCGAAGTGGTCGCACTGTGGATGTTGGTTCTGGAGGATTTGTCGTCCAAAGGAGCAGACGTAGTCCTCAGTTCCATGTGGAGCGTCCTGGTCGCACTGTGGATGTGGGTTCCGGAGGATTTTTTGTCCAAAGGGGCAGGCGTAGTCCCCAGTTTCCTGTGGAGCGACCTTGGCAAAG----------GGCTTTGAAGGAC>line_596ATGGTCTCGCCCAAACGGAGACTAATCGACGATTCCATTGCTCCCTCAGCTGCCGATCTCCAGCGGACATACGACGGACATGATGGGCCTCACGTTTTCGGAACACCCGGCAATCAAGTCTACATTCGGGGGCAGAACGATGGCGTATACAAGGTGCCAGGAGTAGGTGGTCAGTTCCATCACGCATCGTCGCCAGCAGATCACGTCTATACGGATGAGCAGGGGATCACGTATGTGCACAAAAAAGACGCTGGAGGACCAGGAACACATACGCTTAGAGGACCAGGTCTAGCTGCTGTACAGCCCGCCTACTCCACTGTGCAGCCTGCGGGACTTCGCAATCCCCAGTTTCATGTGGAGCGAAGTGGTCGCACTGTGGATGTTGGTTCTGGAGGATTTGTCGTCCAAAGGAGCAGACGTAGTCCTCAGTTCCATGTGGAGCGTCCTGGTCGCACTGTGGATGTGGGTTCCGGAGGATTTTTTGTCCAAAGGGGCAGGCGTAGTCCCCAGTTTCCTGTGGAGCGACCTTGGCAAAG----------GGCTTTGAAGGAC>line_727ATGGTCTCGCCCAAACGGAGACTAATCGACGATTCCATTGCTCCCTCAGCTGCCGATCTCCAGCGGACATACGACGGACATGATGGGCCTCACGTTTTCGGAACACCCGGCAATCAAGTCTACATTCGGGGGCAGAACGATGGCGTATACAAGGTGCCAGGAGTAGGTGGTCAGTTCCATCACGCATCGTCGCCAGCAGATCACGTCTATACGGATGAGCAGGGGATCACGTATGTGCACAAAAAAGACGCTGGAGGACCAGGAACACATACGCTTAGAGGACCAGGTCTAGCTGCTGTACAGCCCGCCTACTCCACTGTGCAGCCTGCGGGACTTCGCAATCCCCAGTTTCATGTGGAGCGAAGTGGTCGCACTGTGGATGTTGGTTCTGGAGGATTTGTCGTCCAAAGGAGCAGACGTAGTCCTCAGTTCCATGTGGAGCGTCCTGGTCGCACTGTGGATGTGGGTTCCGGAGGATTTTTTGTCCAAAGGGGCAGGCGTAGTCCCCAGTTTCCTGTGGAGCGACCTTGGCAAAG----------GGCTTTGAAGGAC>line_761ATGGTCTCGCCCAAACGGAGACTAATCGACGATTCCATTGCTCCCTCAGCTGCCGATCTCCAGCGGACATACGACGGACATGATGGGCCTCACGTTTTCGGAACACCCGGCAATCAAGTCTACATTCGGGGGCAGAACGATGGCGTATACAAGGTGCCAGGAGTAGGTGGTCAGTTCCATCACGCATCGTCGCCAGCAGATCACGTCTATACGGATGAGCAGGGGATCACGTATGTGCACAAAAAAGACGCTGGAGGACCAGGAACACATACGCTTAGAGGACCAGGTCTAGCTGCTGTACAGCCCGCCTACTCCACTGTGCAGCCTGCGGGACTTCGCAATCCCCAGTTTCATGTGGAGCGAAGTGGTCGCACTGTGGATGTTGGTTCTGGAGGATTTGTCGTCCAAAGGAGCAGACGTAGTCCTCAGTTCCATGTGGAGCGTCCTGGTCGCACTGTGGATGTGGGTTCCGGAGGATTTTTTGTCCAAAGGGGCAGGCGTAGTCCCCAGTTTCCTGTGGAGCGACCTTGGCAAAG----------GGCTTTGAAGGAC>line_776ATGGTCTCGCCCAAACGGAGACTAATCGACGATTCCATTGCTCCCTCAGCTGCCGATCTCCAGCGGACATACGACGGACATGATGGGCCTCACGTTTTCGGAACACCCGGCAATCAAGTCTACATTCGGGGGCAGAACGATGGCGTATACAAGGTGCCAGGAGTAGGTGGTCAGTTCCATCACGCATCGTCGCCAGCAGATCACGTCTATACGGATGAGCAGGGGATCACGTATGTGCACAAAAAAGACGCTGGAGGACCAGGAACACATACGCTTAGAGGACCAGGTCTAGCTGCTGTACAGCCCGCCTACTCCACTGTGCAGCCTGCGGGACTTCGCAATCCCCAGTTTCATGTGGAGCGAAGTGGTCGCACTGTGGATGTTGGTTCTGGAGGATTTGTCGTCCAAAGGAGCAGACGTAGTCCTCAGTTCCATGTGGAGCGTCCTGGTCGCACTGTGGATGTGGGTTCCGGAGGATTTTTTGTCCAAAGGGGCAGGCGTAGTCCCCAGTTTCCTGTGGAGCGACCTTGGCAAAG----------GGCTTTGAAGGAC>line_786ATGGTCTCGCCCAAACGGAGACTAATCGACGATTCCATTGCTCCCTCAGCTGCCGATCTCCAGCGGACATACGACGGACATGATGGGCCTCACGTTTTCGGAACACCCGGCAATCAAGTCTACATTCGGGGGCAGAACGATGGCGTATACAAGGTGCCAGGAGTAGGTGGTCAGTTCCATCACGCATCGTCGCCAGCAGATCACGTCTATACGGATGAGCAGGGGATCACGTATGTGCACAAAAAAGACGCTGGAGGACCAGGAACACATACGCTTAGAGGACCAGGTCTAGCTGCTGTACAGCCCGCCTACTCCACTGTGCAGCCTGCGGGACTTCGCAATCCCCAGTTTCATGTGGAGCGAAGTGGTCGCACTGTGGATGTTGGTTCTGGAGGATTTGTCGTCCAAAGGAGCAGACGTAGTCCTCAGTTCCATGTGGAGCGTCCTGGTCGCACTGTGGATGTGGGTTCCGGAGGATTTTTTGTCCAAAGGGGCAGGCGTAGTCCCCAGTTTCCTGTGGAGCGACCTTGGCAAAG----------GGCTTTGAAGGAC>line_787ATGGTCTCGCCCAAACGGAGACTAATCGACGATTCCATTGCTCCCTCAGCTGCCGATCTCCAGCGGACATACGACGGACATGATGGGCCTCACGTTTTCGGAACACCCGGCAATCAAGTCTACATTCGGGGGCAGAACGATGGCGTATACAAGGTGCCAGGAGTAGGTGGTCAGTTCCATCACGCATCGTCGCCAGCAGATCACGTCTATACGGATGAGCAGGGGATCACGTATGTGCACAAAAAAGACGCTGGAGGACCAGGAACACATACGCTTAGAGGACCAGGTCTAGCTGCTGTACAGCCCGCCTACTCCACTGTGCAGCCTGCGGGACTTCGCAATCCCCAGTTTCATGTGGAGCGAAGTGGTCGCACTGTGGATGTTGGTTCTGGAGGATTTGTCGTCCAAAGGAGCAGACGTAGTCCTCAGTTCCATGTGGAGCGTCCTGGTCGCACTGTGGATGTGGGTTCCGGAGGATTTTTTGTCCAAAGGGGCAGGCGTAGTCCCCAGTTTCCTGTGGAGCGACCTTGGCAAAG----------GGCTTTGAAGGAC>line_822ATGGTCTCGCCCAAACGGAGACTAATCGACGATTCCATTGCTCCCTCAGCTGCCGATCTCCAGCGGACATACGACGGACATGATGGGCCTCACGTTTTCGGAACACCCGGCAATCAAGTCTACATTCGGGGGCAGAACGATGGCGTATACAAGGTGCCAGGAGTAGGTGGTCAGTTCCATCACGCATCGTCGCCAGCAGATCACGTCTATACGGATGAGCAGGGGATCACGTATGTGCACAAAAAAGACGCTGGAGGACCAGGAACACATACGCTTAGAGGACCAGGTCTAGCTGCTGTACAGCCCGCCTACTCCACTGTGCAGCCTGCGGGACTTCGCAATCCCCAGTTTCATGTGGAGCGAAGTGGTCGCACTGTGGATGTTGGTTCTGGAGGATTTGTCGTCCAAAGGAGCAGACGTAGTCCTCAGTTCCATGTGGAGCGTCCTGGTCGCACTGTGGATGTGGGTTCCGGAGGATTTTTTGTCCAAAGGGGCAGGCGTAGTCCCCAGTTTCCTGTGGAGCGACCTTGGCAAAG----------GGCTTTGAAGGAC>line_832ATGGTCTCGCCCAAACGGAGACTAATCGACGATTCCATTGCTCCCTCAGCTGCCGATCTCCAGCGGACATACGACGGACATGATGGGCCTCACGTTTTCGGAACACCCGGCAATCAAGTCTACATTCGGGGGCAGAACGATGGCGTATACAAGGTGCCAGGAGTAGGTGGTCAGTTCCATCACGCATCGTCGCCAGCAGATCACGTCTATACGGATGAGCAGGGGATCACGTATGTGCACAAAAAAGACGCTGGAGGACCAGGAACACATACGCTTAGAGGACCAGGTCTAGCTGCTGTACAGCCCGCCTACTCCACTGTGCAGCCTGCGGGACTTCGCAATCCCCAGTTTCATGTGGAGCGAAGTGGTCGCACTGTGGATGTTGGTTCTGGAGGATTTGTCGTCCAAAGGAGCAGACGTAGTCCTCAGTTCCATGTGGAGCGTCCTGGTCGCACTGTGGATGTGGGTTCCGGAGGATTTTTTGTCCAAAGGGGCAGGCGTAGTCCCCAGTTTCCTGTGGAGCGACCTTGGCAAAG----------GGCTTTGAAGGAC>line_843ATGGTCTCGCCCAAACGGAGACTAATCGACGATTCCATTGCTCCCTCAGCTGCCGATCTCCAGCGGACATACGACGGACATGATGGGCCTCACGTTTTCGGAACACCCGGCAATCAAGTCTACATTCGGGGGCAGAACGATGGCGTATACAAGGTGCCAGGAGTAGGTGGTCAGTTCCATCACGCATCGTCGCCAGCAGATCACGTCTATACGGATGAGCAGGGGATCACGTATGTGCACAAAAAAGACGCTGGAGGACCAGGAACACATACGCTTAGAGGACCAGGTCTAGCTGCTGTACAGCCCGCCTACTCCACTGTGCAGCCTGCGGGACTTCGCAATCCCCAGTTTCATGTGGAGCGAAGTGGTCGCACTGTGGATGTTGGTTCTGGAGGATTTGTCGTCCAAAGGAGCAGACGTAGTCCTCAGTTCCATGTGGAGCGTCCTGGTCGCACTGTGGATGTGGGTTCCGGAGGATTTTTTGTCCAAAGGGGCAGGCGTAGTCCCCAGTTTCCTGTGGAGCGACCTTGGCAAAG----------GGCTTTGAAGGAC>line_850ATGGTCTCGCCCAAACGGAGACTAATCGACGATTCCATTGCTCCCTCAGCTGCCGATCTCCAGCGGACATACGACGGACATGATGGGCCTCACGTTTTCGGAACACCCGGCAATCAAGTCTACATTCGGGGGCAGAACGATGGCGTATACAAGGTGCCAGGAGTAGGTGGTCAGTTCCATCACGCATCGTCGCCAGCAGATCACGTCTATACGGATGAGCAGGGGATCACGTATGTGCACAAAAAAGACGCTGGAGGACCAGGAACACATACGCTTAGAGGACCAGGTCTAGCTGCTGTACAGCCCGCCTACTCCACTGTGCAGCCTGCGGGACTTCGCAATCCCCAGTTTCATGTGGAGCGAAGTGGTCGCACTGTGGATGTTGGTTCTGGAGGATTTGTCGTCCAAAGGAGCAGACGTAGTCCTCAGTTCCATGTGGAGCGTCCTGGTCGCACTGTGGATGTGGGTTCCGGAGGATTTTTTGTCCAAAGGGGCAGGCGTAGTCCCCAGTTTCCTGTGGAGCGACCTTGGCAAAG----------GGCTTTGAAGGAC>line_882ATGGTCTCGCCCAAACGGAGACTAATCGACGATTCCATTGCTCCCTCAGCTGCCGATCTCCAGCGGACATACGACGGACATGATGGGCCTCACGTTTTCGGAACACCCGGCAATCAAGTCTACATTCGGGGGCAGAACGATGGCGTATACAAGGTGCCAGGAGTAGGTGGTCAGTTCCATCACGCATCGTCGCCAGCAGATCACGTCTATACGGATGAGCAGGGGATCACGTATGTGCACAAAAAAGACGCTGGAGGACCAGGAACACATACGCTTAGAGGACCAGGTCTAGCTGCTGTACAGCCCGCCTACTCCACTGTGCAGCCTGCGGGACTTCGCAATCCCCAGTTTCATGTGGAGCGAAGTGGTCGCACTGTGGATGTTGGTTCTGGAGGATTTGTCGTCCAAAGGAGCAGACGTAGTCCTCAGTTCCATGTGGAGCGTCCTGGTCGCACTGTGGATGTGGGTTCCGGAGGATTTTTTGTCCAAAGGGGCAGGCGTAGTCCCCAGTTTCCTGTGGAGCGACCTTGGCAAAG----------GGCTTTGAAGGAC>line_884ATGGTCTCGCCCAAACGGAGACTAATCGACGATTCCATTGCTCCCTCAGCTGCCGATCTCCAGCGGACATACGACGGACATGATGGGCCTCACGTTTTCGGAACACCCGGCAATCAAGTCTACATTCGGGGGCAGAACGATGGCGTATACAAGGTGCCAGGAGTAGGTGGTCAGTTCCATCACGCATCGTCGCCAGCAGATCACGTCTATACGGATGAGCAGGGGATCACGTATGTGCACAAAAAAGACGCTGGAGGACCAGGAACACATACGCTTAGAGGACCAGGTCTAGCTGCTGTACAGCCCGCCTACTCCACTGTGCAGCCTGCGGGACTTCGCAATCCCCAGTTTCATGTGGAGCGAAGTGGTCGCACTGTGGATGTTGGTTCTGGAGGATTTGTCGTCCAAAGGAGCAGACGTAGTCCTCAGTTCCATGTGGAGCGTCCTGGTCGCACTGTGGATGTGGGTTCCGGAGGATTTTTTGTCCAAAGGGGCAGGCGTAGTCCCCAGTTTCCTGTGGAGCGACCTTGGCAAAG----------GGCTTTGAAGGAC>line_887ATGGTCTCGCCCAAACGGAGACTAATCGACGATTCCATTGCTCCCTCAGCTGCCGATCTCCAGCGGACATACGACGGACATGATGGGCCTCACGTTTTCGGAACACCCGGCAATCAAGTCTACATTCGGGGGCAGAACGATGGCGTATACAAGGTGCCAGGAGTAGGTGGTCAGTTCCATCACGCATCGTCGCCAGCAGATCACGTCTATACGGATGAGCAGGGGATCACGTATGTGCACAAAAAAGACGCTGGAGGACCAGGAACACATACGCTTAGAGGACCAGGTCTAGCTGCTGTACAGCCCGCCTACTCCACTGTGCAGCCTGCGGGACTTCGCAATCCCCAGTTTCATGTGGAGCGAAGTGGTCGCACTGTGGATGTTGGTTCTGGAGGATTTGTCGTCCAAAGGAGCAGACGTAGTCCTCAGTTCCATGTGGAGCGTCCTGGTCGCACTGTGGATGTGGGTTCCGGAGGATTTTTTGTCCAAAGGGGCAGGCGTAGTCCCCAGTTTCCTGTGGAGCGACCTTGGCAAAG----------GGCTTTGAAGGAC>line_900ATGGTCTCGCCCAAACGGAGACTAATCGACGATTCCATTGCTCCCTCAGCTGCCGATCTCCAGCGGACATACGACGGACATGATGGGCCTCACGTTTTCGGAACACCCGGCAATCAAGTCTACATTCGGGGGCAGAACGATGGCGTATACAAGGTGCCAGGAGTAGGTGGTCAGTTCCATCACGCATCGTCGCCAGCAGATCACGTCTATACGGATGAGCAGGGGATCACGTATGTGCACAAAAAAGACGCTGGAGGACCAGGAACACATACGCTTAGAGGACCAGGTCTAGCTGCTGTACAGCCCGCCTACTCCACTGTGCAGCCTGCGGGACTTCGCAATCCCCAGTTTCATGTGGAGCGAAGTGGTCGCACTGTGGATGTTGGTTCTGGAGGATTTGTCGTCCAAAGGAGCAGACGTAGTCCTCAGTTCCATGTGGAGCGTCCTGGTCGCACTGTGGATGTGGGTTCCGGAGGATTTTTTGTCCAAAGGGGCAGGCGTAGTCCCCAGTTTCCTGTGGAGCGACCTTGGCAAAG----------GGCTTTGAAGGAC>line_237ATGGTCTCGCCCAAACGGAGACTAATCGACGATTCCATTGCTCCCTCAGCTGCCGATCTCCAGCGGACATACGACGGACATGATGGGCCTCACGTTTTCGGAACACCCGGCAATCAAGTCTACATTCGGGGGCAGAACGATGGCGTATACAAGGTGCCAGGAGTAGGTGGTCAGTTCCATCACGCATCGTCGCCAGCAGATCACGTCTATACGGATGAGCAGGGGATCACGTATGTGCACAAAAAAGACGCTGGAGGACCAGGAACACATACGCTTAGAGGACCAGGTCTAGCTGCTGTACAGCCCGCCTACTCCACTGTGAAGCCTGCGGGACTTCGCAATCCCCAGTTTCATGTGGAGCGAAGTGGTCGCACTGTGGATGTTGGTTCTGGAGGATTTGTCGTCCAAAGGAGCAGACGTAGTCCTCAGTTCCATGTGGAGCGTCCTGGTCGCACTGTGGATGTGGGTTCCGGAGGATTTTTTGTCCAAAGGGGCAGGCGTAGTCCCCAGTTTCCTGTGGAGCGACCTTGGCAAAG----------GGCTTTGAAGGAC>line_634ATGGTCTCGCCCAAACGGAGACTAATCGACGATTCCATTGCTCCCTCAGCTGCCGATCTCCAGCGGACATACGACGGACATGATGGGCCTCACGTTTTCGGAACACCCGGCAATCAAGTCTACATTCGGGGGCAGAACGATGGCGTATACAAGGTGCCAGGAGTAGGTGGTCAGTTCCATCACGCATCGTCGCCAGCAGATCACGTCTATACGGATGAGCAGGGGATCACGTATGTGCACAAAAAAGACGCTGGAGGACCAGGAACACATACGCTTAGAGGACCAGGTCTAGCTGCTGTACAGCCCGCCTACTCCACTGTGAAGCCTGCGGGACTTCGCAATCCCCAGTTTCATGTGGAGCGAAGTGGTCGCACTGTGGATGTTGGTTCTGGAGGATTTGTCGTCCAAAGGAGCAGACGTAGTCCTCAGTTCCATGTGGAGCGTCCTGGTCGCACTGTGGATGTGGGTTCCGGAGGATTTTTTGTCCAAAGGGGCAGGCGTAGTCCCCAGTTTCCTGTGGAGCGACCTTGGCAAAG----------GGCTTTGAAGGAC>line_703ATGGTCTCGCCCAAACGGAGACTAATCGACGATTCCATTGCTCCCTCAGCTGCCGATCTCCAGCGGACATACGACGGACATGATGGGCCTCACGTTTTCGGAACACCCGGCAATCAAGTCTACATTCGGGGGCAGAACGATGGCGTATACAAGGTGCCAGGAGTAGGTGGTCAGTTCCATCACGCATCGTCGCCAGCAGATCACGTCTATACGGATGAGCAGGGGATCACGTATGTGCACAAAAAAGACGCTGGAGGACCAGGAACACATACGCTTAGAGGACCAGGTCTAGCTGCTGTACAGCCCGCCTACTCCACTGTGAAGCCTGCGGGACTTCGCAATCCCCAGTTTCATGTGGAGCGAAGTGGTCGCACTGTGGATGTTGGTTCTGGAGGATTTGTCGTCCAAAGGAGCAGACGTAGTCCTCAGTTCCATGTGGAGCGTCCTGGTCGCACTGTGGATGTGGGTTCCGGAGGATTTTTTGTCCAAAGGGGCAGGCGTAGTCCCCAGTTTCCTGTGGAGCGACCTTGGCAAAG----------GGCTTTGAAGGAC>line_301ATGGTCTCGCCCAAACGGAGACTAATCGACGATTCCATTGCTCCCTCAGCTGCCGATCTCCAGCGGACATACGACGGACATGATGGGCCTCACGTTTTCGGAACACCCGGCAATCAAGTCTACATTCGGGGGCAGAACGATGGCGTATACAAGGTGCCAGGAGTAGGTGGTCAGTTCCATCACGCATCGTCGCCAGCAGAACACGTCTATACGGATGAGCAGGGGATCACGTATGTGCACAAAAAAGACGCTGGAGGACCAGGAACACATACGCTTAGAGGACCAGGTCTAGCTGCTGTACAGCCCGCCTACTCCACTGTGAAGCCTGCGGGACTTCGCAATCCCCAGTTTCATGTGGAGCGAAGTGGTCGCACTGTGGATGTTGGTTCTGGAGGATTTGTCGTCCAAAGGAGCAGACGTAGTCCTCAGTTCCATGTGGAGCGTCCTGGTCGCACTGTGGATGTGGGTTCCGGAGGATTTTTTGTCCAAAGGGGCAGGCGTAGTCCCCAGTTTCCTGTGGAGCGACCTTGGCAAAG----------GGCTTTGAAGGAC>line_149ATGGTCTCGCCCAAACGGAGACTAATCGACGATTCCATTGCTCCCTCAGCTGCCGATCTCCAGCGGACATACGACGGACATGATGGGCCTCACGTTTTCGGAACACCCGGCAATCAAGTCTACATTCGGGGGCAGAACGATGGCGTATACAAGGTGCCAGGAGTAGGTGGTCAGTTCCATCACGCATCGTCGCCAGCAGAACACGTCTATACGGATGAGCAGGGGATCACGTATGTGCACAAAAAAGACGCTGGAGGACCAGGAACACATACGCTTAGAGGACCAGGTCTAGCTGCTGTACAGCCCGCCTACTCCACTGTGCAGCCTGCGGGACTTCGCAGTCCCCAGTTTCATGTGGAGCGAAGTGGTCGCACTGTGGATGTTGGTTCTGGAGGATTTGTCGTCCAAAGGAGCAGACGTAGTCCTCAGTTCCATGTGGAGCGTCCTGGTCGCACTGTGGATGTGGGTTCCGGAGGATTTTTTGTCCAAAGGGGCAGGCGTAGTCCCCAGTTTCCTGTGGAGCGACCTTGGCAAAG----------GGCTTTGAAGGAC>line_177ATGGTCTCGCCCAAACGGAGACTAATCGACGATTCCATTGCTCCCTCAGCTGCCGATCTCCAGCGGACATACGACGGACATGATGGGCCTCACGTTTTCGGAACACCCGGCAATCAAGTCTACATTCGGGGGCAGAACGATGGCGTATACAAGGTGCCAGGAGTAGGTGGTCAGTTCCATCACGCATCGTCGCCAGCAGAACACGTCTATACGGATGAGCAGGGGATCACGTATGTGCACAAAAAAGACGCTGGAGGACCAGGAACACATACGCTTAGAGGACCAGGTCTAGCTGCTGTACAGCCCGCCTACTCCACTGTGCAGCCTGCGGGACTTCGCAGTCCCCAGTTTCATGTGGAGCGAAGTGGTCGCACTGTGGATGTTGGTTCTGGAGGATTTGTCGTCCAAAGGAGCAGACGTAGTCCTCAGTTCCATGTGGAGCGTCCTGGTCGCACTGTGGATGTGGGTTCCGGAGGATTTTTTGTCCAAAGGGGCAGGCGTAGTCCCCAGTTTCCTGTGGAGCGACCTTGGCAAAG----------GGCTTTGAAGGAC>line_361ATGGTCTCGCCCAAACGGAGACTAATCGACGATTCCATTGCTCCCTCAGCTGCCGATCTCCAGCGGACATACGACGGACATGATGGGCCTCACGTTTTCGGAACACCCGGCAATCAAGTCTACATTCGGGGGCAGAACGATGGCGTATACAAGGTGCCAGGAGTAGGTGGTCAGTTCCATCACGCATCGTCGCCAGCAGAACACGTCTATACGGATGAGCAGGGGATCACGTATGTGCACAAAAAAGACGCTGGAGGACCAGGAACACATACGCTTAGAGGACCAGGTCTAGCTGCTGTACAGCCCGCCTACTCCACTGTGCAGCCTGCGGGACTTCGCAGTCCCCAGTTTCATGTGGAGCGAAGTGGTCGCACTGTGGATGTTGGTTCTGGAGGATTTGTCGTCCAAAGGAGCAGACGTAGTCCTCAGTTCCATGTGGAGCGTCCTGGTCGCACTGTGGATGTGGGTTCCGGAGGATTTTTTGTCCAAAGGGGCAGGCGTAGTCCCCAGTTTCCTGTGGAGCGACCTTGGCAAAG----------GGCTTTGAAGGAC>line_373ATGGTCTCGCCCAAACGGAGACTAATCGACGATTCCATTGCTCCCTCAGCTGCCGATCTCCAGCGGACATACGACGGACATGATGGGCCTCACGTTTTCGGAACACCCGGCAATCAAGTCTACATTCGGGGGCAGAACGATGGCGTATACAAGGTGCCAGGAGTAGGTGGTCAGTTCCATCACGCATCGTCGCCAGCAGAACACGTCTATACGGATGAGCAGGGGATCACGTATGTGCACAAAAAAGACGCTGGAGGACCAGGAACACATACGCTTAGAGGACCAGGTCTAGCTGCTGTACAGCCCGCCTACTCCACTGTGCAGCCTGCGGGACTTCGCAGTCCCCAGTTTCATGTGGAGCGAAGTGGTCGCACTGTGGATGTTGGTTCTGGAGGATTTGTCGTCCAAAGGAGCAGACGTAGTCCTCAGTTCCATGTGGAGCGTCCTGGTCGCACTGTGGATGTGGGTTCCGGAGGATTTTTTGTCCAAAGGGGCAGGCGTAGTCCCCAGTTTCCTGTGGAGCGACCTTGGCAAAG----------GGCTTTGAAGGAC>line_461ATGGTCTCGCCCAAACGGAGACTAATCGACGATTCCATTGCTCCCTCAGCTGCCGATCTCCAGCGGACATACGACGGACATGATGGGCCTCACGTTTTCGGAACACCCGGCAATCAAGTCTACATTCGGGGGCAGAACGATGGCGTATACAAGGTGCCAGGAGTAGGTGGTCAGTTCCATCACGCATCGTCGCCAGCAGAACACGTCTATACGGATGAGCAGGGGATCACGTATGTGCACAAAAAAGACGCTGGAGGACCAGGAACACATACGCTTAGAGGACCAGGTCTAGCTGCTGTACAGCCCGCCTACTCCACTGTGCAGCCTGCGGGACTTCGCAGTCCCCAGTTTCATGTGGAGCGAAGTGGTCGCACTGTGGATGTTGGTTCTGGAGGATTTGTCGTCCAAAGGAGCAGACGTAGTCCTCAGTTCCATGTGGAGCGTCCTGGTCGCACTGTGGATGTGGGTTCCGGAGGATTTTTTGTCCAAAGGGGCAGGCGTAGTCCCCAGTTTCCTGTGGAGCGACCTTGGCAAAG----------GGCTTTGAAGGAC>line_502ATGGTCTCGCCCAAACGGAGACTAATCGACGATTCCATTGCTCCCTCAGCTGCCGATCTCCAGCGGACATACGACGGACATGATGGGCCTCACGTTTTCGGAACACCCGGCAATCAAGTCTACATTCGGGGGCAGAACGATGGCGTATACAAGGTGCCAGGAGTAGGTGGTCAGTTCCATCACGCATCGTCGCCAGCAGAACACGTCTATACGGATGAGCAGGGGATCACGTATGTGCACAAAAAAGACGCTGGAGGACCAGGAACACATACGCTTAGAGGACCAGGTCTAGCTGCTGTACAGCCCGCCTACTCCACTGTGCAGCCTGCGGGACTTCGCAGTCCCCAGTTTCATGTGGAGCGAAGTGGTCGCACTGTGGATGTTGGTTCTGGAGGATTTGTCGTCCAAAGGAGCAGACGTAGTCCTCAGTTCCATGTGGAGCGTCCTGGTCGCACTGTGGATGTGGGTTCCGGAGGATTTTTTGTCCAAAGGGGCAGGCGTAGTCCCCAGTTTCCTGTGGAGCGACCTTGGCAAAG----------GGCTTTGAAGGAC>line_642ATGGTCTCGCCCAAACGGAGACTAATCGACGATTCCATTGCTCCCTCAGCTGCCGATCTCCAGCGGACATACGACGGACATGATGGGCCTCACGTTTTCGGAACACCCGGCAATCAAGTCTACATTCGGGGGCAGAACGATGGCGTATACAAGGTGCCAGGAGTAGGTGGTCAGTTCCATCACGCATCGTCGCCAGCAGAACACGTCTATACGGATGAGCAGGGGATCACGTATGTGCACAAAAAAGACGCTGGAGGACCAGGAACACATACGCTTAGAGGACCAGGTCTAGCTGCTGTACAGCCCGCCTACTCCACTGTGCAGCCTGCGGGACTTCGCAGTCCCCAGTTTCATGTGGAGCGAAGTGGTCGCACTGTGGATGTTGGTTCTGGAGGATTTGTCGTCCAAAGGAGCAGACGTAGTCCTCAGTTCCATGTGGAGCGTCCTGGTCGCACTGTGGATGTGGGTTCCGGAGGATTTTTTGTCCAAAGGGGCAGGCGTAGTCCCCAGTTTCCTGTGGAGCGACCTTGGCAAAG----------GGCTTTGAAGGAC>line_646ATGGTCTCGCCCAAACGGAGACTAATCGACGATTCCATTGCTCCCTCAGCTGCCGATCTCCAGCGGACATACGACGGACATGATGGGCCTCACGTTTTCGGAACACCCGGCAATCAAGTCTACATTCGGGGGCAGAACGATGGCGTATACAAGGTGCCAGGAGTAGGTGGTCAGTTCCATCACGCATCGTCGCCAGCAGAACACGTCTATACGGATGAGCAGGGGATCACGTATGTGCACAAAAAAGACGCTGGAGGACCAGGAACACATACGCTTAGAGGACCAGGTCTAGCTGCTGTACAGCCCGCCTACTCCACTGTGCAGCCTGCGGGACTTCGCAGTCCCCAGTTTCATGTGGAGCGAAGTGGTCGCACTGTGGATGTTGGTTCTGGAGGATTTGTCGTCCAAAGGAGCAGACGTAGTCCTCAGTTCCATGTGGAGCGTCCTGGTCGCACTGTGGATGTGGGTTCCGGAGGATTTTTTGTCCAAAGGGGCAGGCGTAGTCCCCAGTTTCCTGTGGAGCGACCTTGGCAAAG----------GGCTTTGAAGGAC>line_712ATGGTCTCGCCCAAACGGAGACTAATCGACGATTCCATTGCTCCCTCAGCTGCCGATCTCCAGCGGACATACGACGGACATGATGGGCCTCACGTTTTCGGAACACCCGGCAATCAAGTCTACATTCGGGGGCAGAACGATGGCGTATACAAGGTGCCAGGAGTAGGTGGTCAGTTCCATCACGCATCGTCGCCAGCAGAACACGTCTATACGGATGAGCAGGGGATCACGTATGTGCACAAAAAAGACGCTGGAGGACCAGGAACACATACGCTTAGAGGACCAGGTCTAGCTGCTGTACAGCCCGCCTACTCCACTGTGCAGCCTGCGGGACTTCGCAGTCCCCAGTTTCATGTGGAGCGAAGTGGTCGCACTGTGGATGTTGGTTCTGGAGGATTTGTCGTCCAAAGGAGCAGACGTAGTCCTCAGTTCCATGTGGAGCGTCCTGGTCGCACTGTGGATGTGGGTTCCGGAGGATTTTTTGTCCAAAGGGGCAGGCGTAGTCCCCAGTTTCCTGTGGAGCGACCTTGGCAAAG----------GGCTTTGAAGGAC>line_819ATGGTCTCGCCCAAACGGAGACTAATCGACGATTCCATTGCTCCCTCAGCTGCCGATCTCCAGCGGACATACGACGGACATGATGGGCCTCACGTTTTCGGAACACCCGGCAATCAAGTCTACATTCGGGGGCAGAACGATGGCGTATACAAGGTGCCAGGAGTAGGTGGTCAGTTCCATCACGCATCGTCGCCAGCAGAACACGTCTATACGGATGAGCAGGGGATCACGTATGTGCACAAAAAAGACGCTGGAGGACCAGGAACACATACGCTTAGAGGACCAGGTCTAGCTGCTGTACAGCCCGCCTACTCCACTGTGCAGCCTGCGGGACTTCGCAGTCCCCAGTTTCATGTGGAGCGAAGTGGTCGCACTGTGGATGTTGGTTCTGGAGGATTTGTCGTCCAAAGGAGCAGACGTAGTCCTCAGTTCCATGTGGAGCGTCCTGGTCGCACTGTGGATGTGGGTTCCGGAGGATTTTTTGTCCAAAGGGGCAGGCGTAGTCCCCAGTTTCCTGTGGAGCGACCTTGGCAAAG----------GGCTTTGAAGGAC>line_158ATGGTCTCGCCCAAACGGAGACTAATCGACGATTCCATTGCTCCCTCAGCTGCCGATCTCCAGCGGACATACGACGGACATGATGGGCCTCACGTTTTCGGAACACCCGGCAATCAAGTCTACATTCGGGGGCAGAACGATGGCGTATACAAGGTGCCAGGAGTAGGTGGTCAGTTCCATCACGCATCGTCGCCAGCAGAACACGTCTATACGGATGAGCAGGGGATCACGTATGTGCACAAAAAAGACGCTGGAGGACCAGGAACACATACGCTTAGAGGACCAGGTCTAGCTGCTGTACAGCCCGCCTACTCCACTGTGCAGCCTGCGGGACTTCGCAATCCCCAGTTTCATGTGGAGCGAAGTGGTCGCACTGTGGATGTTGGTTCTGGAGGATTTGTCGTCCAAAGGAGCAGACGTAGTCCTCAGTTCCATGTGGAGCGTCCTGGTCGCACTGTGGATGTGGGTTCCGGAGGATTTTTTGTCCAAAGGGGCAGGCGTAGTCCCCAGTTTCCTGTGGAGCGACCTTGGCAAAG----------GGCTTTGAAGGAC>line_229ATGGTCTCGCCCAAACGGAGACTAATCGACGATTCCATTGCTCCCTCAGCTGCCGATCTCCAGCGGACATACGACGGACATGATGGGCCTCACGTTTTCGGAACACCCGGCAATCAAGTCTACATTCGGGGGCAGAACGATGGCGTATACAAGGTGCCAGGAGTAGGTGGTCAGTTCCATCACGCATCGTCGCCAGCAGAACACGTCTATACGGATGAGCAGGGGATCACGTATGTGCACAAAAAAGACGCTGGAGGACCAGGAACACATACGCTTAGAGGACCAGGTCTAGCTGCTGTACAGCCCGCCTACTCCACTGTGCAGCCTGCGGGACTTCGCAATCCCCAGTTTCATGTGGAGCGAAGTGGTCGCACTGTGGATGTTGGTTCTGGAGGATTTGTCGTCCAAAGGAGCAGACGTAGTCCTCAGTTCCATGTGGAGCGTCCTGGTCGCACTGTGGATGTGGGTTCCGGAGGATTTTTTGTCCAAAGGGGCAGGCGTAGTCCCCAGTTTCCTGTGGAGCGACCTTGGCAAAG----------GGCTTTGAAGGAC>line_310ATGGTCTCGCCCAAACGGAGACTAATCGACGATTCCATTGCTCCCTCAGCTGCCGATCTCCAGCGGACATACGACGGACATGATGGGCCTCACGTTTTCGGAACACCCGGCAATCAAGTCTACATTCGGGGGCAGAACGATGGCGTATACAAGGTGCCAGGAGTAGGTGGTCAGTTCCATCACGCATCGTCGCCAGCAGAACACGTCTATACGGATGAGCAGGGGATCACGTATGTGCACAAAAAAGACGCTGGAGGACCAGGAACACATACGCTTAGAGGACCAGGTCTAGCTGCTGTACAGCCCGCCTACTCCACTGTGCAGCCTGCGGGACTTCGCAATCCCCAGTTTCATGTGGAGCGAAGTGGTCGCACTGTGGATGTTGGTTCTGGAGGATTTGTCGTCCAAAGGAGCAGACGTAGTCCTCAGTTCCATGTGGAGCGTCCTGGTCGCACTGTGGATGTGGGTTCCGGAGGATTTTTTGTCCAAAGGGGCAGGCGTAGTCCCCAGTTTCCTGTGGAGCGACCTTGGCAAAG----------GGCTTTGAAGGAC>line_324ATGGTCTCGCCCAAACGGAGACTAATCGACGATTCCATTGCTCCCTCAGCTGCCGATCTCCAGCGGACATACGACGGACATGATGGGCCTCACGTTTTCGGAACACCCGGCAATCAAGTCTACATTCGGGGGCAGAACGATGGCGTATACAAGGTGCCAGGAGTAGGTGGTCAGTTCCATCACGCATCGTCGCCAGCAGAACACGTCTATACGGATGAGCAGGGGATCACGTATGTGCACAAAAAAGACGCTGGAGGACCAGGAACACATACGCTTAGAGGACCAGGTCTAGCTGCTGTACAGCCCGCCTACTCCACTGTGCAGCCTGCGGGACTTCGCAATCCCCAGTTTCATGTGGAGCGAAGTGGTCGCACTGTGGATGTTGGTTCTGGAGGATTTGTCGTCCAAAGGAGCAGACGTAGTCCTCAGTTCCATGTGGAGCGTCCTGGTCGCACTGTGGATGTGGGTTCCGGAGGATTTTTTGTCCAAAGGGGCAGGCGTAGTCCCCAGTTTCCTGTGGAGCGACCTTGGCAAAG----------GGCTTTGAAGGAC>line_340ATGGTCTCGCCCAAACGGAGACTAATCGACGATTCCATTGCTCCCTCAGCTGCCGATCTCCAGCGGACATACGACGGACATGATGGGCCTCACGTTTTCGGAACACCCGGCAATCAAGTCTACATTCGGGGGCAGAACGATGGCGTATACAAGGTGCCAGGAGTAGGTGGTCAGTTCCATCACGCATCGTCGCCAGCAGAACACGTCTATACGGATGAGCAGGGGATCACGTATGTGCACAAAAAAGACGCTGGAGGACCAGGAACACATACGCTTAGAGGACCAGGTCTAGCTGCTGTACAGCCCGCCTACTCCACTGTGCAGCCTGCGGGACTTCGCAATCCCCAGTTTCATGTGGAGCGAAGTGGTCGCACTGTGGATGTTGGTTCTGGAGGATTTGTCGTCCAAAGGAGCAGACGTAGTCCTCAGTTCCATGTGGAGCGTCCTGGTCGCACTGTGGATGTGGGTTCCGGAGGATTTTTTGTCCAAAGGGGCAGGCGTAGTCCCCAGTTTCCTGTGGAGCGACCTTGGCAAAG----------GGCTTTGAAGGAC>line_365ATGGTCTCGCCCAAACGGAGACTAATCGACGATTCCATTGCTCCCTCAGCTGCCGATCTCCAGCGGACATACGACGGACATGATGGGCCTCACGTTTTCGGAACACCCGGCAATCAAGTCTACATTCGGGGGCAGAACGATGGCGTATACAAGGTGCCAGGAGTAGGTGGTCAGTTCCATCACGCATCGTCGCCAGCAGAACACGTCTATACGGATGAGCAGGGGATCACGTATGTGCACAAAAAAGACGCTGGAGGACCAGGAACACATACGCTTAGAGGACCAGGTCTAGCTGCTGTACAGCCCGCCTACTCCACTGTGCAGCCTGCGGGACTTCGCAATCCCCAGTTTCATGTGGAGCGAAGTGGTCGCACTGTGGATGTTGGTTCTGGAGGATTTGTCGTCCAAAGGAGCAGACGTAGTCCTCAGTTCCATGTGGAGCGTCCTGGTCGCACTGTGGATGTGGGTTCCGGAGGATTTTTTGTCCAAAGGGGCAGGCGTAGTCCCCAGTTTCCTGTGGAGCGACCTTGGCAAAG----------GGCTTTGAAGGAC>line_513ATGGTCTCGCCCAAACGGAGACTAATCGACGATTCCATTGCTCCCTCAGCTGCCGATCTCCAGCGGACATACGACGGACATGATGGGCCTCACGTTTTCGGAACACCCGGCAATCAAGTCTACATTCGGGGGCAGAACGATGGCGTATACAAGGTGCCAGGAGTAGGTGGTCAGTTCCATCACGCATCGTCGCCAGCAGAACACGTCTATACGGATGAGCAGGGGATCACGTATGTGCACAAAAAAGACGCTGGAGGACCAGGAACACATACGCTTAGAGGACCAGGTCTAGCTGCTGTACAGCCCGCCTACTCCACTGTGCAGCCTGCGGGACTTCGCAATCCCCAGTTTCATGTGGAGCGAAGTGGTCGCACTGTGGATGTTGGTTCTGGAGGATTTGTCGTCCAAAGGAGCAGACGTAGTCCTCAGTTCCATGTGGAGCGTCCTGGTCGCACTGTGGATGTGGGTTCCGGAGGATTTTTTGTCCAAAGGGGCAGGCGTAGTCCCCAGTTTCCTGTGGAGCGACCTTGGCAAAG----------GGCTTTGAAGGAC>line_566ATGGTCTCGCCCAAACGGAGACTAATCGACGATTCCATTGCTCCCTCAGCTGCCGATCTCCAGCGGACATACGACGGACATGATGGGCCTCACGTTTTCGGAACACCCGGCAATCAAGTCTACATTCGGGGGCAGAACGATGGCGTATACAAGGTGCCAGGAGTAGGTGGTCAGTTCCATCACGCATCGTCGCCAGCAGAACACGTCTATACGGATGAGCAGGGGATCACGTATGTGCACAAAAAAGACGCTGGAGGACCAGGAACACATACGCTTAGAGGACCAGGTCTAGCTGCTGTACAGCCCGCCTACTCCACTGTGCAGCCTGCGGGACTTCGCAATCCCCAGTTTCATGTGGAGCGAAGTGGTCGCACTGTGGATGTTGGTTCTGGAGGATTTGTCGTCCAAAGGAGCAGACGTAGTCCTCAGTTCCATGTGGAGCGTCCTGGTCGCACTGTGGATGTGGGTTCCGGAGGATTTTTTGTCCAAAGGGGCAGGCGTAGTCCCCAGTTTCCTGTGGAGCGACCTTGGCAAAG----------GGCTTTGAAGGAC>line_714ATGGTCTCGCCCAAACGGAGACTAATCGACGATTCCATTGCTCCCTCAGCTGCCGATCTCCAGCGGACATACGACGGACATGATGGGCCTCACGTTTTCGGAACACCCGGCAATCAAGTCTACATTCGGGGGCAGAACGATGGCGTATACAAGGTGCCAGGAGTAGGTGGTCAGTTCCATCACGCATCGTCGCCAGCAGAACACGTCTATACGGATGAGCAGGGGATCACGTATGTGCACAAAAAAGACGCTGGAGGACCAGGAACACATACGCTTAGAGGACCAGGTCTAGCTGCTGTACAGCCCGCCTACTCCACTGTGCAGCCTGCGGGACTTCGCAATCCCCAGTTTCATGTGGAGCGAAGTGGTCGCACTGTGGATGTTGGTTCTGGAGGATTTGTCGTCCAAAGGAGCAGACGTAGTCCTCAGTTCCATGTGGAGCGTCCTGGTCGCACTGTGGATGTGGGTTCCGGAGGATTTTTTGTCCAAAGGGGCAGGCGTAGTCCCCAGTTTCCTGTGGAGCGACCTTGGCAAAG----------GGCTTTGAAGGAC>line_812ATGGTCTCGCCCAAACGGAGACTAATCGACGATTCCATTGCTCCCTCAGCTGCCGATCTCCAGCGGACATACGACGGACATGATGGGCCTCACGTTTTCGGAACACCCGGCAATCAAGTCTACATTCGGGGGCAGAACGATGGCGTATACAAGGTGCCAGGAGTAGGTGGTCAGTTCCATCACGCATCGTCGCCAGCAGAACACGTCTATACGGATGAGCAGGGGATCACGTATGTGCACAAAAAAGACGCTGGAGGACCAGGAACACATACGCTTAGAGGACCAGGTCTAGCTGCTGTACAGCCCGCCTACTCCACTGTGCAGCCTGCGGGACTTCGCAATCCCCAGTTTCATGTGGAGCGAAGTGGTCGCACTGTGGATGTTGGTTCTGGAGGATTTGTCGTCCAAAGGAGCAGACGTAGTCCTCAGTTCCATGTGGAGCGTCCTGGTCGCACTGTGGATGTGGGTTCCGGAGGATTTTTTGTCCAAAGGGGCAGGCGTAGTCCCCAGTTTCCTGTGGAGCGACCTTGGCAAAG----------GGCTTTGAAGGAC>line_853ATGGTCTCGCCCAAACGGAGACTAATCGACGATTCCATTGCTCCCTCAGCTGCCGATCTCCAGCGGACATACGACGGACATGATGGGCCTCACGTTTTCGGAACACCCGGCAATCAAGTCTACATTCGGGGGCAGAACGATGGCGTATACAAGGTGCCAGGAGTAGGTGGTCAGTTCCATCACGCATCGTCGCCAGCAGAACACGTCTATACGGATGAGCAGGGGATCACGTATGTGCACAAAAAAGACGCTGGAGGACCAGGAACACATACGCTTAGAGGACCAGGTCTAGCTGCTGTACAGCCCGCCTACTCCACTGTGCAGCCTGCGGGACTTCGCAATCCCCAGTTTCATGTGGAGCGAAGTGGTCGCACTGTGGATGTTGGTTCTGGAGGATTTGTCGTCCAAAGGAGCAGACGTAGTCCTCAGTTCCATGTGGAGCGTCCTGGTCGCACTGTGGATGTGGGTTCCGGAGGATTTTTTGTCCAAAGGGGCAGGCGTAGTCCCCAGTTTCCTGTGGAGCGACCTTGGCAAAG----------GGCTTTGAAGGAC>line_859ATGGTCTCGCCCAAACGGAGACTAATCGACGATTCCATTGCTCCCTCAGCTGCCGATCTCCAGCGGACATACGACGGACATGATGGGCCTCACGTTTTCGGAACACCCGGCAATCAAGTCTACATTCGGGGGCAGAACGATGGCGTATACAAGGTGCCAGGAGTAGGTGGTCAGTTCCATCACGCATCGTCGCCAGCAGAACACGTCTATACGGATGAGCAGGGGATCACGTATGTGCACAAAAAAGACGCTGGAGGACCAGGAACACATACGCTTAGAGGACCAGGTCTAGCTGCTGTACAGCCCGCCTACTCCACTGTGCAGCCTGCGGGACTTCGCAATCCCCAGTTTCATGTGGAGCGAAGTGGTCGCACTGTGGATGTTGGTTCTGGAGGATTTGTCGTCCAAAGGAGCAGACGTAGTCCTCAGTTCCATGTGGAGCGTCCTGGTCGCACTGTGGATGTGGGTTCCGGAGGATTTTTTGTCCAAAGGGGCAGGCGTAGTCCCCAGTTTCCTGTGGAGCGACCTTGGCAAAG----------GGCTTTGAAGGAC>line_913ATGGTCTCGCCCAAACGGAGACTAATCGACGATTCCATTGCTCCCTCAGCTGCCGATCTCCAGCGGACATACGACGGACATGATGGGCCTCACGTTTTCGGAACACCCGGCAATCAAGTCTACATTCGGGGGCAGAACGATGGCGTATACAAGGTGCCAGGAGTAGGTGGTCAGTTCCATCACGCATCGTCGCCAGCAGAACACGTCTATACGGATGAGCAGGGGATCACGTATGTGCACAAAAAAGACGCTGGAGGACCAGGAACACATACGCTTAGAGGACCAGGTCTAGCTGCTGTACAGCCCGCCTACTCCACTGTGCAGCCTGCGGGACTTCGCAATCCCCAGTTTCATGTGGAGCGAAGTGGTCGCACTGTGGATGTTGGTTCTGGAGGATTTGTCGTCCAAAGGAGCAGACGTAGTCCTCAGTTCCATGTGGAGCGTCCTGGTCGCACTGTGGATGTGGGTTCCGGAGGATTTTTTGTCCAAAGGGGCAGGCGTAGTCCCCAGTTTCCTGTGGAGCGACCTTGGCAAAG----------GGCTTTGAAGGAC>line_181ATGGTCTCGCCCAAACGGAGACTAATCGACGATTCCATTGCTCCCTCAGCTGCCGATCTCCAGCGGACATACGACGGACATGATGGGCCTCACGTTTTCGGAACACCCGGCAATCAAGTCTACATTCGGGGGCAGAACGATGGCGTATACAAGGTGCCAGGAGTAGGTGGTCAGTTCCATCACGCATCGTCGCCAGCAGAACACGTCTATACGGATGAGCAGGGGATCACGTATGTGCACAAAAAAGACGCTGGAGGACCAGGAACACATACGCTTAGAGGACCAGGTCTAGCTGCTGTACAGCCCGCCTACTCCACTGTGCAGCCTGCGGGACTTCGCAATCCCCAGTTTCATGTGGAGCGAAGTGGTCGCACTGTGGATGTTGGTTCTGGAGGATTTGTCGTCCAAAGGAGCAGACGTAGTCCTCAGTTCCATGTGGAGCGTCCTGGTCGCACTGTGGATGTGGGTTCCGGAGGATTTTTTGTCCAAAGGGGCAGGCGTAGTCCCCAGTTTCCTGTGGAGCGACCTTGGCAAAG----------GGCTTTAAGGGAC>line_707ATGGTCTCGCCCAAACGGAGACTAATCGACGATTCCATTGCTCCCTCAGCTGCCGATCTCCAGCGGACATACGACGGACATGATGGGCCTCACGTTTTCGGAACACCCGGCAATCAAGTCTACATTCGGGGGCAGAACGATGGCGTATACAAGGTGCCAGGAGTAGGTGGTCAGTTCCATCACGCATCGTCGCCAGCAGAACACGTCTATACGGATGAGCAGGGGATCACGTATGTGCACAAAAAAGACGCTGGAGGACCAGGAACACATACGCTTAGAGGACCAGGTCTAGCTGCTGTACAGCCCGCCTACTCCACTGTGCAGCCTGCGGGACTTCGCAATCCCCAGTTTCATGTGGAGCGAAGTGGTCGCACTGTGGATGTTGGTTCTGGAGGATTTGTCGTCCAAAGGAGCAGACGTAGTCCTCAGTTCCATGTGGAGCGTCCTGGTCGCACTGTGGATGTGGGTTCCGGAGGATTTTTTGTCCAAAGGGGCAGGCGTAGTCCCCAGTTTCCTGTGGAGCGACCTTGGCAAAG----------GGCTTTAAGGGAC>line_857ATGGTCTCGCCCAAACGGAGACTAATCGACGATTCCATTGCTCCCTCAGCTGCCGATCTCCAGCGGACATACGACGGACATGATGGGCCTCACGTTTTCGGAACACCCGGCAATCAAGTCTACATTCGGGGGCAGAACGATGGCGTATACAAGGTGCCAGGAGTAGGTGGTCAGTTCCATCACGCATCGTCGCCAGCAGAACACGTCTATACGGATGAGCAGGGGATCACGTATGTGCACAAAAAAGACGCTGGAGGACCAGGAACACATACGCTTAGAGGACCAGGTCTAGCTGCTGTACAGCCCGCCTACTCCACTGTGCAGCCTGCGGGACTTCGCAATCCCCAGTTTCATGTGGAGCGAAGTGGTCGCACTGTGGATGTTGGTTCTGGAGGATTTGTCGTCCAAAGGAGCAGACGTAGTCCTCAGTTCCATGTGGAGCGTCCTGGTCGCACTGTGGATGTGGGTTCCGGAGGATTTTTTGTCCAAAGGGGCAGGCGTAGTCCCCAGTTTCCTGTGGAGCGACCTTGGCAAAG----------GGCTTTAAGGGAC>line_304ATGGTCTCGCCCAAACGGAGACTAATCGACGATTCCATTGCTCCCTCAGCTGCCGATCTCCAGCGGACATACGACGGACATGATGGGCCTCACGTTTTCGGAACACCCGGCAATCAAGTCTACATTCTGGGGCAGAACGATGGCGTATACAAGGTGCCAGGAGTAGGTGGTCAGTTCCATCACGCATCGTCGCCAGCAGAACACGTCTATACGGATGAGCAGGGGATCACGTATGTGCACAAAAAAGACGCTGGAGGACCAGGAACACATACGCTTAGAGGACCAGGTCTAGCTGCTGTACAGCCCGCCTACTCCACTGTGCAGCCTGCGGGACTTCGCAATCCCCAGTTTCATGTGGAGCGAAGTGGTCGCACTGTGGATGTTGGTTCTGGAGGATTTGTCGTCCAAAGGAGCAGACGTAGTCCTCAGTTCCATGTGGAGCGTCCTGGTCGCACTGTGGATGTGGGTTCCGGAGGATTTTTTGTCCAAAGGGGCAGGCGTAGTCCCCAGTTTCCTGTGGAGCGACCTTGGCAAAG----------GGCTTTGAAGGAC>line_31ATGGTCTCGCCCAAACGGAGACTAATCGACGATTCCATTGCTCCCTCAGCTGCCGATCTCCAGCGGACATACGACGGACATGATGGGCCTCAATTTTTCGGAACACCCGGCAATCAAGTCTACATTCGGGGGCAGAACGATGGCGTATACAAGGTGCCAGGAGTAGGTGGTCAGTTCCATCACGCATCGTCGCCAGCAGAACACGTCTATACGGATGAGCAGGGGATCACGTATGTGCACAAAAAAGACGCTGGAGGACCAGGAACACATACGCTTAGAGGACCAGGTCTAGCTGCTGTACAGCCCGCCTACTCCACTGTGCAGCCTGCGGGACTTCGCAATCCCCAGTTTCATGTGGAGCGAAGTGGTCGCACTGTGGATGTTGGTTCTGGAGGATTTGTCGTCCAAAGGAGCAGACGTAGTCCTCAGTTCCATGTGGAGCGTCCTGGTCGCACTGTGGATGTGGGTTCCGGAGGATTTTTTGTCCAAAGGGGCAGGCGTAGTCCCCAGTTTCCTGTGGAGCGACCTTGGCAAAG----------GGCTTTGAAGGAC>line_315ATGGTCTCGCCCAAACGGAGACTAATCGACGATTCCATTGCTCCCTCAGCTGCCGATCTCCAGCGGACATACGACGGACATGATGGGCCTCAATTTTTCGGAACACCCGGCAATCAAGTCTACATTCGGGGGCAGAACGATGGCGTATACAAGGTGCCAGGAGTAGGTGGTCAGTTCCATCACGCATCGTCGCCAGCAGAACACGTCTATACGGATGAGCAGGGGATCACGTATGTGCACAAAAAAGACGCTGGAGGACCAGGAACACATACGCTTAGAGGACCAGGTCTAGCTGCTGTACAGCCCGCCTACTCCACTGTGCAGCCTGCGGGACTTCGCAATCCCCAGTTTCATGTGGAGCGAAGTGGTCGCACTGTGGATGTTGGTTCTGGAGGATTTGTCGTCCAAAGGAGCAGACGTAGTCCTCAGTTCCATGTGGAGCGTCCTGGTCGCACTGTGGATGTGGGTTCCGGAGGATTTTTTGTCCAAAGGGGCAGGCGTAGTCCCCAGTTTCCTGTGGAGCGACCTTGGCAAAG----------GGCTTTGAAGGAC>line_382ATGGTCTCGCCCAAACGGAGACTAATCGACGATTCCATTGCTCCCTCAGCTGCCGATCTCCAGCGGACATACGACGGACATGATGGGCCTCAATTTTTCGGAACACCCGGCAATCAAGTCTACATTCGGGGGCAGAACGATGGCGTATACAAGGTGCCAGGAGTAGGTGGTCAGTTCCATCACGCATCGTCGCCAGCAGAACACGTCTATACGGATGAGCAGGGGATCACGTATGTGCACAAAAAAGACGCTGGAGGACCAGGAACACATACGCTTAGAGGACCAGGTCTAGCTGCTGTACAGCCCGCCTACTCCACTGTGCAGCCTGCGGGACTTCGCAATCCCCAGTTTCATGTGGAGCGAAGTGGTCGCACTGTGGATGTTGGTTCTGGAGGATTTGTCGTCCAAAGGAGCAGACGTAGTCCTCAGTTCCATGTGGAGCGTCCTGGTCGCACTGTGGATGTGGGTTCCGGAGGATTTTTTGTCCAAAGGGGCAGGCGTAGTCCCCAGTTTCCTGTGGAGCGACCTTGGCAAAG----------GGCTTTGAAGGAC>line_406ATGGTCTCGCCCAAACGGAGACTAATCGACGATTCCATTGCTCCCTCAGCTGCCGATCTCCAGCGGACATACGACGGACATGATGGGCCTCAATTTTTCGGAACACCCGGCAATCAAGTCTACATTCGGGGGCAGAACGATGGCGTATACAAGGTGCCAGGAGTAGGTGGTCAGTTCCATCACGCATCGTCGCCAGCAGAACACGTCTATACGGATGAGCAGGGGATCACGTATGTGCACAAAAAAGACGCTGGAGGACCAGGAACACATACGCTTAGAGGACCAGGTCTAGCTGCTGTACAGCCCGCCTACTCCACTGTGCAGCCTGCGGGACTTCGCAATCCCCAGTTTCATGTGGAGCGAAGTGGTCGCACTGTGGATGTTGGTTCTGGAGGATTTGTCGTCCAAAGGAGCAGACGTAGTCCTCAGTTCCATGTGGAGCGTCCTGGTCGCACTGTGGATGTGGGTTCCGGAGGATTTTTTGTCCAAAGGGGCAGGCGTAGTCCCCAGTTTCCTGTGGAGCGACCTTGGCAAAG----------GGCTTTGAAGGAC>line_426ATGGTCTCGCCCAAACGGAGACTAATCGACGATTCCATTGCTCCCTCAGCTGCCGATCTCCAGCGGACATACGACGGACATGATGGGCCTCAATTTTTCGGAACACCCGGCAATCAAGTCTACATTCGGGGGCAGAACGATGGCGTATACAAGGTGCCAGGAGTAGGTGGTCAGTTCCATCACGCATCGTCGCCAGCAGAACACGTCTATACGGATGAGCAGGGGATCACGTATGTGCACAAAAAAGACGCTGGAGGACCAGGAACACATACGCTTAGAGGACCAGGTCTAGCTGCTGTACAGCCCGCCTACTCCACTGTGCAGCCTGCGGGACTTCGCAATCCCCAGTTTCATGTGGAGCGAAGTGGTCGCACTGTGGATGTTGGTTCTGGAGGATTTGTCGTCCAAAGGAGCAGACGTAGTCCTCAGTTCCATGTGGAGCGTCCTGGTCGCACTGTGGATGTGGGTTCCGGAGGATTTTTTGTCCAAAGGGGCAGGCGTAGTCCCCAGTTTCCTGTGGAGCGACCTTGGCAAAG----------GGCTTTGAAGGAC>line_530ATGGTCTCGCCCAAACGGAGACTAATCGACGATTCCATTGCTCCCTCAGCTGCCGATCTCCAGCGGACATACGACGGACATGATGGGCCTCAATTTTTCGGAACACCCGGCAATCAAGTCTACATTCGGGGGCAGAACGATGGCGTATACAAGGTGCCAGGAGTAGGTGGTCAGTTCCATCACGCATCGTCGCCAGCAGAACACGTCTATACGGATGAGCAGGGGATCACGTATGTGCACAAAAAAGACGCTGGAGGACCAGGAACACATACGCTTAGAGGACCAGGTCTAGCTGCTGTACAGCCCGCCTACTCCACTGTGCAGCCTGCGGGACTTCGCAATCCCCAGTTTCATGTGGAGCGAAGTGGTCGCACTGTGGATGTTGGTTCTGGAGGATTTGTCGTCCAAAGGAGCAGACGTAGTCCTCAGTTCCATGTGGAGCGTCCTGGTCGCACTGTGGATGTGGGTTCCGGAGGATTTTTTGTCCAAAGGGGCAGGCGTAGTCCCCAGTTTCCTGTGGAGCGACCTTGGCAAAG----------GGCTTTGAAGGAC>line_908ATGGTCTCGCCCAAACGGAGACTAATCGACGATTCCATTGCTCCCTCAGCTGCCGATCTCCAGCGGACATACGACGGACATGATGGGCCTCAATTTTTCGGAACACCCGGCAATCAAGTCTACATTCGGGGGCAGAACGATGGCGTATACAAGGTGCCAGGAGTAGGTGGTCAGTTCCATCACGCATCGTCGCCAGCAGAACACGTCTATACGGATGAGCAGGGGATCACGTATGTGCACAAAAAAGACGCTGGAGGACCAGGAACACATACGCTTAGAGGACCAGGTCTAGCTGCTGTACAGCCCGCCTACTCCACTGTGCAGCCTGCGGGACTTCGCAATCCCCAGTTTCATGTGGAGCGAAGTGGTCGCACTGTGGATGTTGGTTCTGGAGGATTTGTCGTCCAAAGGAGCAGACGTAGTCCTCAGTTCCATGTGGAGCGTCCTGGTCGCACTGTGGATGTGGGTTCCGGAGGATTTTTTGTCCAAAGGGGCAGGCGTAGTCCCCAGTTTCCTGTGGAGCGACCTTGGCAAAG----------GGCTTTGAAGGAC>line_26ATGGTCTCGCCCAAACGGAGACTAATCGACGATTCCATTGCTCCCTCAGCTGCCGATCTCCAGCGGACATACGACGGACATGATGGGCCTCACGTTTTCGGAACACCCGGCAATCAAGTCTACATTCGGGGGCAGAACGATGGCGTATACAAGGTGCCAGGAGTAGGTGGTCAGTTCCATCACGCATCGTCGCCAGCAGATCACGTCTATACGGATGAGCAGGGGATCACGTATGTGCACAAAAAAGACGCTGGAGGACCAGGAACACATACGCTTAGAGGACCAGGTCTAGCTGCTGTACAGCCCGCCTACTCCACTGTGCAGCCTGCGGGACTTCGCAATCCCCAGTTTCATGTGGAGCGAAGTGGTCGCACTGTGGATGTTGGTTCTGGAGGATTTGTCGTCCAAAGGAGCAGACGTAGTCCTCAGTTCCATGTGGAGCGTCCTGGTCGCACTGTGGATGTGGGTTCCGGAGGATTTTTTGTCCAAAGGGGCAGGCGTAGTCCCCAGTTTCCTGTGGAGCGACCTTGGCAAAGGGTCGACGTCGGCTTTGAAGGAC>line_28ATGGTCTCGCCCAAACGGAGACTAATCGACGATTCCATTGCTCCCTCAGCTGCCGATCTCCAGCGGACATACGACGGACATGATGGGCCTCACGTTTTCGGAACACCCGGCAATCAAGTCTACATTCGGGGGCAGAACGATGGCGTATACAAGGTGCCAGGAGTAGGTGGTCAGTTCCATCACGCATCGTCGCCAGCAGATCACGTCTATACGGATGAGCAGGGGATCACGTATGTGCACAAAAAAGACGCTGGAGGACCAGGAACACATACGCTTAGAGGACCAGGTCTAGCTGCTGTACAGCCCGCCTACTCCACTGTGCAGCCTGCGGGACTTCGCAATCCCCAGTTTCATGTGGAGCGAAGTGGTCGCACTGTGGATGTTGGTTCTGGAGGATTTGTCGTCCAAAGGAGCAGACGTAGTCCTCAGTTCCATGTGGAGCGTCCTGGTCGCACTGTGGATGTGGGTTCCGGAGGATTTTTTGTCCAAAGGGGCAGGCGTAGTCCCCAGTTTCCTGTGGAGCGACCTTGGCAAAGGGTCGACGTCGGCTTTGAAGGAC>line_38ATGGTCTCGCCCAAACGGAGACTAATCGACGATTCCATTGCTCCCTCAGCTGCCGATCTCCAGCGGACATACGACGGACATGATGGGCCTCACGTTTTCGGAACACCCGGCAATCAAGTCTACATTCGGGGGCAGAACGATGGCGTATACAAGGTGCCAGGAGTAGGTGGTCAGTTCCATCACGCATCGTCGCCAGCAGATCACGTCTATACGGATGAGCAGGGGATCACGTATGTGCACAAAAAAGACGCTGGAGGACCAGGAACACATACGCTTAGAGGACCAGGTCTAGCTGCTGTACAGCCCGCCTACTCCACTGTGCAGCCTGCGGGACTTCGCAATCCCCAGTTTCATGTGGAGCGAAGTGGTCGCACTGTGGATGTTGGTTCTGGAGGATTTGTCGTCCAAAGGAGCAGACGTAGTCCTCAGTTCCATGTGGAGCGTCCTGGTCGCACTGTGGATGTGGGTTCCGGAGGATTTTTTGTCCAAAGGGGCAGGCGTAGTCCCCAGTTTCCTGTGGAGCGACCTTGGCAAAGGGTCGACGTCGGCTTTGAAGGAC>line_41ATGGTCTCGCCCAAACGGAGACTAATCGACGATTCCATTGCTCCCTCAGCTGCCGATCTCCAGCGGACATACGACGGACATGATGGGCCTCACGTTTTCGGAACACCCGGCAATCAAGTCTACATTCGGGGGCAGAACGATGGCGTATACAAGGTGCCAGGAGTAGGTGGTCAGTTCCATCACGCATCGTCGCCAGCAGATCACGTCTATACGGATGAGCAGGGGATCACGTATGTGCACAAAAAAGACGCTGGAGGACCAGGAACACATACGCTTAGAGGACCAGGTCTAGCTGCTGTACAGCCCGCCTACTCCACTGTGCAGCCTGCGGGACTTCGCAATCCCCAGTTTCATGTGGAGCGAAGTGGTCGCACTGTGGATGTTGGTTCTGGAGGATTTGTCGTCCAAAGGAGCAGACGTAGTCCTCAGTTCCATGTGGAGCGTCCTGGTCGCACTGTGGATGTGGGTTCCGGAGGATTTTTTGTCCAAAGGGGCAGGCGTAGTCCCCAGTTTCCTGTGGAGCGACCTTGGCAAAGGGTCGACGTCGGCTTTGAAGGAC>line_45ATGGTCTCGCCCAAACGGAGACTAATCGACGATTCCATTGCTCCCTCAGCTGCCGATCTCCAGCGGACATACGACGGACATGATGGGCCTCACGTTTTCGGAACACCCGGCAATCAAGTCTACATTCGGGGGCAGAACGATGGCGTATACAAGGTGCCAGGAGTAGGTGGTCAGTTCCATCACGCATCGTCGCCAGCAGATCACGTCTATACGGATGAGCAGGGGATCACGTATGTGCACAAAAAAGACGCTGGAGGACCAGGAACACATACGCTTAGAGGACCAGGTCTAGCTGCTGTACAGCCCGCCTACTCCACTGTGCAGCCTGCGGGACTTCGCAATCCCCAGTTTCATGTGGAGCGAAGTGGTCGCACTGTGGATGTTGGTTCTGGAGGATTTGTCGTCCAAAGGAGCAGACGTAGTCCTCAGTTCCATGTGGAGCGTCCTGGTCGCACTGTGGATGTGGGTTCCGGAGGATTTTTTGTCCAAAGGGGCAGGCGTAGTCCCCAGTTTCCTGTGGAGCGACCTTGGCAAAGGGTCGACGTCGGCTTTGAAGGAC>line_48ATGGTCTCGCCCAAACGGAGACTAATCGACGATTCCATTGCTCCCTCAGCTGCCGATCTCCAGCGGACATACGACGGACATGATGGGCCTCACGTTTTCGGAACACCCGGCAATCAAGTCTACATTCGGGGGCAGAACGATGGCGTATACAAGGTGCCAGGAGTAGGTGGTCAGTTCCATCACGCATCGTCGCCAGCAGATCACGTCTATACGGATGAGCAGGGGATCACGTATGTGCACAAAAAAGACGCTGGAGGACCAGGAACACATACGCTTAGAGGACCAGGTCTAGCTGCTGTACAGCCCGCCTACTCCACTGTGCAGCCTGCGGGACTTCGCAATCCCCAGTTTCATGTGGAGCGAAGTGGTCGCACTGTGGATGTTGGTTCTGGAGGATTTGTCGTCCAAAGGAGCAGACGTAGTCCTCAGTTCCATGTGGAGCGTCCTGGTCGCACTGTGGATGTGGGTTCCGGAGGATTTTTTGTCCAAAGGGGCAGGCGTAGTCCCCAGTTTCCTGTGGAGCGACCTTGGCAAAGGGTCGACGTCGGCTTTGAAGGAC>line_49ATGGTCTCGCCCAAACGGAGACTAATCGACGATTCCATTGCTCCCTCAGCTGCCGATCTCCAGCGGACATACGACGGACATGATGGGCCTCACGTTTTCGGAACACCCGGCAATCAAGTCTACATTCGGGGGCAGAACGATGGCGTATACAAGGTGCCAGGAGTAGGTGGTCAGTTCCATCACGCATCGTCGCCAGCAGATCACGTCTATACGGATGAGCAGGGGATCACGTATGTGCACAAAAAAGACGCTGGAGGACCAGGAACACATACGCTTAGAGGACCAGGTCTAGCTGCTGTACAGCCCGCCTACTCCACTGTGCAGCCTGCGGGACTTCGCAATCCCCAGTTTCATGTGGAGCGAAGTGGTCGCACTGTGGATGTTGGTTCTGGAGGATTTGTCGTCCAAAGGAGCAGACGTAGTCCTCAGTTCCATGTGGAGCGTCCTGGTCGCACTGTGGATGTGGGTTCCGGAGGATTTTTTGTCCAAAGGGGCAGGCGTAGTCCCCAGTTTCCTGTGGAGCGACCTTGGCAAAGGGTCGACGTCGGCTTTGAAGGAC>line_57ATGGTCTCGCCCAAACGGAGACTAATCGACGATTCCATTGCTCCCTCAGCTGCCGATCTCCAGCGGACATACGACGGACATGATGGGCCTCACGTTTTCGGAACACCCGGCAATCAAGTCTACATTCGGGGGCAGAACGATGGCGTATACAAGGTGCCAGGAGTAGGTGGTCAGTTCCATCACGCATCGTCGCCAGCAGATCACGTCTATACGGATGAGCAGGGGATCACGTATGTGCACAAAAAAGACGCTGGAGGACCAGGAACACATACGCTTAGAGGACCAGGTCTAGCTGCTGTACAGCCCGCCTACTCCACTGTGCAGCCTGCGGGACTTCGCAATCCCCAGTTTCATGTGGAGCGAAGTGGTCGCACTGTGGATGTTGGTTCTGGAGGATTTGTCGTCCAAAGGAGCAGACGTAGTCCTCAGTTCCATGTGGAGCGTCCTGGTCGCACTGTGGATGTGGGTTCCGGAGGATTTTTTGTCCAAAGGGGCAGGCGTAGTCCCCAGTTTCCTGTGGAGCGACCTTGGCAAAGGGTCGACGTCGGCTTTGAAGGAC>line_73ATGGTCTCGCCCAAACGGAGACTAATCGACGATTCCATTGCTCCCTCAGCTGCCGATCTCCAGCGGACATACGACGGACATGATGGGCCTCACGTTTTCGGAACACCCGGCAATCAAGTCTACATTCGGGGGCAGAACGATGGCGTATACAAGGTGCCAGGAGTAGGTGGTCAGTTCCATCACGCATCGTCGCCAGCAGATCACGTCTATACGGATGAGCAGGGGATCACGTATGTGCACAAAAAAGACGCTGGAGGACCAGGAACACATACGCTTAGAGGACCAGGTCTAGCTGCTGTACAGCCCGCCTACTCCACTGTGCAGCCTGCGGGACTTCGCAATCCCCAGTTTCATGTGGAGCGAAGTGGTCGCACTGTGGATGTTGGTTCTGGAGGATTTGTCGTCCAAAGGAGCAGACGTAGTCCTCAGTTCCATGTGGAGCGTCCTGGTCGCACTGTGGATGTGGGTTCCGGAGGATTTTTTGTCCAAAGGGGCAGGCGTAGTCCCCAGTTTCCTGTGGAGCGACCTTGGCAAAGGGTCGACGTCGGCTTTGAAGGAC>line_75ATGGTCTCGCCCAAACGGAGACTAATCGACGATTCCATTGCTCCCTCAGCTGCCGATCTCCAGCGGACATACGACGGACATGATGGGCCTCACGTTTTCGGAACACCCGGCAATCAAGTCTACATTCGGGGGCAGAACGATGGCGTATACAAGGTGCCAGGAGTAGGTGGTCAGTTCCATCACGCATCGTCGCCAGCAGATCACGTCTATACGGATGAGCAGGGGATCACGTATGTGCACAAAAAAGACGCTGGAGGACCAGGAACACATACGCTTAGAGGACCAGGTCTAGCTGCTGTACAGCCCGCCTACTCCACTGTGCAGCCTGCGGGACTTCGCAATCCCCAGTTTCATGTGGAGCGAAGTGGTCGCACTGTGGATGTTGGTTCTGGAGGATTTGTCGTCCAAAGGAGCAGACGTAGTCCTCAGTTCCATGTGGAGCGTCCTGGTCGCACTGTGGATGTGGGTTCCGGAGGATTTTTTGTCCAAAGGGGCAGGCGTAGTCCCCAGTTTCCTGTGGAGCGACCTTGGCAAAGGGTCGACGTCGGCTTTGAAGGAC>line_83ATGGTCTCGCCCAAACGGAGACTAATCGACGATTCCATTGCTCCCTCAGCTGCCGATCTCCAGCGGACATACGACGGACATGATGGGCCTCACGTTTTCGGAACACCCGGCAATCAAGTCTACATTCGGGGGCAGAACGATGGCGTATACAAGGTGCCAGGAGTAGGTGGTCAGTTCCATCACGCATCGTCGCCAGCAGATCACGTCTATACGGATGAGCAGGGGATCACGTATGTGCACAAAAAAGACGCTGGAGGACCAGGAACACATACGCTTAGAGGACCAGGTCTAGCTGCTGTACAGCCCGCCTACTCCACTGTGCAGCCTGCGGGACTTCGCAATCCCCAGTTTCATGTGGAGCGAAGTGGTCGCACTGTGGATGTTGGTTCTGGAGGATTTGTCGTCCAAAGGAGCAGACGTAGTCCTCAGTTCCATGTGGAGCGTCCTGGTCGCACTGTGGATGTGGGTTCCGGAGGATTTTTTGTCCAAAGGGGCAGGCGTAGTCCCCAGTTTCCTGTGGAGCGACCTTGGCAAAGGGTCGACGTCGGCTTTGAAGGAC>line_85ATGGTCTCGCCCAAACGGAGACTAATCGACGATTCCATTGCTCCCTCAGCTGCCGATCTCCAGCGGACATACGACGGACATGATGGGCCTCACGTTTTCGGAACACCCGGCAATCAAGTCTACATTCGGGGGCAGAACGATGGCGTATACAAGGTGCCAGGAGTAGGTGGTCAGTTCCATCACGCATCGTCGCCAGCAGATCACGTCTATACGGATGAGCAGGGGATCACGTATGTGCACAAAAAAGACGCTGGAGGACCAGGAACACATACGCTTAGAGGACCAGGTCTAGCTGCTGTACAGCCCGCCTACTCCACTGTGCAGCCTGCGGGACTTCGCAATCCCCAGTTTCATGTGGAGCGAAGTGGTCGCACTGTGGATGTTGGTTCTGGAGGATTTGTCGTCCAAAGGAGCAGACGTAGTCCTCAGTTCCATGTGGAGCGTCCTGGTCGCACTGTGGATGTGGGTTCCGGAGGATTTTTTGTCCAAAGGGGCAGGCGTAGTCCCCAGTTTCCTGTGGAGCGACCTTGGCAAAGGGTCGACGTCGGCTTTGAAGGAC>line_88ATGGTCTCGCCCAAACGGAGACTAATCGACGATTCCATTGCTCCCTCAGCTGCCGATCTCCAGCGGACATACGACGGACATGATGGGCCTCACGTTTTCGGAACACCCGGCAATCAAGTCTACATTCGGGGGCAGAACGATGGCGTATACAAGGTGCCAGGAGTAGGTGGTCAGTTCCATCACGCATCGTCGCCAGCAGATCACGTCTATACGGATGAGCAGGGGATCACGTATGTGCACAAAAAAGACGCTGGAGGACCAGGAACACATACGCTTAGAGGACCAGGTCTAGCTGCTGTACAGCCCGCCTACTCCACTGTGCAGCCTGCGGGACTTCGCAATCCCCAGTTTCATGTGGAGCGAAGTGGTCGCACTGTGGATGTTGGTTCTGGAGGATTTGTCGTCCAAAGGAGCAGACGTAGTCCTCAGTTCCATGTGGAGCGTCCTGGTCGCACTGTGGATGTGGGTTCCGGAGGATTTTTTGTCCAAAGGGGCAGGCGTAGTCCCCAGTTTCCTGTGGAGCGACCTTGGCAAAGGGTCGACGTCGGCTTTGAAGGAC>line_91ATGGTCTCGCCCAAACGGAGACTAATCGACGATTCCATTGCTCCCTCAGCTGCCGATCTCCAGCGGACATACGACGGACATGATGGGCCTCACGTTTTCGGAACACCCGGCAATCAAGTCTACATTCGGGGGCAGAACGATGGCGTATACAAGGTGCCAGGAGTAGGTGGTCAGTTCCATCACGCATCGTCGCCAGCAGATCACGTCTATACGGATGAGCAGGGGATCACGTATGTGCACAAAAAAGACGCTGGAGGACCAGGAACACATACGCTTAGAGGACCAGGTCTAGCTGCTGTACAGCCCGCCTACTCCACTGTGCAGCCTGCGGGACTTCGCAATCCCCAGTTTCATGTGGAGCGAAGTGGTCGCACTGTGGATGTTGGTTCTGGAGGATTTGTCGTCCAAAGGAGCAGACGTAGTCCTCAGTTCCATGTGGAGCGTCCTGGTCGCACTGTGGATGTGGGTTCCGGAGGATTTTTTGTCCAAAGGGGCAGGCGTAGTCCCCAGTTTCCTGTGGAGCGACCTTGGCAAAGGGTCGACGTCGGCTTTGAAGGAC>line_100ATGGTCTCGCCCAAACGGAGACTAATCGACGATTCCATTGCTCCCTCAGCTGCCGATCTCCAGCGGACATACGACGGACATGATGGGCCTCACGTTTTCGGAACACCCGGCAATCAAGTCTACATTCGGGGGCAGAACGATGGCGTATACAAGGTGCCAGGAGTAGGTGGTCAGTTCCATCACGCATCGTCGCCAGCAGATCACGTCTATACGGATGAGCAGGGGATCACGTATGTGCACAAAAAAGACGCTGGAGGACCAGGAACACATACGCTTAGAGGACCAGGTCTAGCTGCTGTACAGCCCGCCTACTCCACTGTGCAGCCTGCGGGACTTCGCAATCCCCAGTTTCATGTGGAGCGAAGTGGTCGCACTGTGGATGTTGGTTCTGGAGGATTTGTCGTCCAAAGGAGCAGACGTAGTCCTCAGTTCCATGTGGAGCGTCCTGGTCGCACTGTGGATGTGGGTTCCGGAGGATTTTTTGTCCAAAGGGGCAGGCGTAGTCCCCAGTTTCCTGTGGAGCGACCTTGGCAAAGGGTCGACGTCGGCTTTGAAGGAC>line_105ATGGTCTCGCCCAAACGGAGACTAATCGACGATTCCATTGCTCCCTCAGCTGCCGATCTCCAGCGGACATACGACGGACATGATGGGCCTCACGTTTTCGGAACACCCGGCAATCAAGTCTACATTCGGGGGCAGAACGATGGCGTATACAAGGTGCCAGGAGTAGGTGGTCAGTTCCATCACGCATCGTCGCCAGCAGATCACGTCTATACGGATGAGCAGGGGATCACGTATGTGCACAAAAAAGACGCTGGAGGACCAGGAACACATACGCTTAGAGGACCAGGTCTAGCTGCTGTACAGCCCGCCTACTCCACTGTGCAGCCTGCGGGACTTCGCAATCCCCAGTTTCATGTGGAGCGAAGTGGTCGCACTGTGGATGTTGGTTCTGGAGGATTTGTCGTCCAAAGGAGCAGACGTAGTCCTCAGTTCCATGTGGAGCGTCCTGGTCGCACTGTGGATGTGGGTTCCGGAGGATTTTTTGTCCAAAGGGGCAGGCGTAGTCCCCAGTTTCCTGTGGAGCGACCTTGGCAAAGGGTCGACGTCGGCTTTGAAGGAC>line_109ATGGTCTCGCCCAAACGGAGACTAATCGACGATTCCATTGCTCCCTCAGCTGCCGATCTCCAGCGGACATACGACGGACATGATGGGCCTCACGTTTTCGGAACACCCGGCAATCAAGTCTACATTCGGGGGCAGAACGATGGCGTATACAAGGTGCCAGGAGTAGGTGGTCAGTTCCATCACGCATCGTCGCCAGCAGATCACGTCTATACGGATGAGCAGGGGATCACGTATGTGCACAAAAAAGACGCTGGAGGACCAGGAACACATACGCTTAGAGGACCAGGTCTAGCTGCTGTACAGCCCGCCTACTCCACTGTGCAGCCTGCGGGACTTCGCAATCCCCAGTTTCATGTGGAGCGAAGTGGTCGCACTGTGGATGTTGGTTCTGGAGGATTTGTCGTCCAAAGGAGCAGACGTAGTCCTCAGTTCCATGTGGAGCGTCCTGGTCGCACTGTGGATGTGGGTTCCGGAGGATTTTTTGTCCAAAGGGGCAGGCGTAGTCCCCAGTTTCCTGTGGAGCGACCTTGGCAAAGGGTCGACGTCGGCTTTGAAGGAC>line_136ATGGTCTCGCCCAAACGGAGACTAATCGACGATTCCATTGCTCCCTCAGCTGCCGATCTCCAGCGGACATACGACGGACATGATGGGCCTCACGTTTTCGGAACACCCGGCAATCAAGTCTACATTCGGGGGCAGAACGATGGCGTATACAAGGTGCCAGGAGTAGGTGGTCAGTTCCATCACGCATCGTCGCCAGCAGATCACGTCTATACGGATGAGCAGGGGATCACGTATGTGCACAAAAAAGACGCTGGAGGACCAGGAACACATACGCTTAGAGGACCAGGTCTAGCTGCTGTACAGCCCGCCTACTCCACTGTGCAGCCTGCGGGACTTCGCAATCCCCAGTTTCATGTGGAGCGAAGTGGTCGCACTGTGGATGTTGGTTCTGGAGGATTTGTCGTCCAAAGGAGCAGACGTAGTCCTCAGTTCCATGTGGAGCGTCCTGGTCGCACTGTGGATGTGGGTTCCGGAGGATTTTTTGTCCAAAGGGGCAGGCGTAGTCCCCAGTTTCCTGTGGAGCGACCTTGGCAAAGGGTCGACGTCGGCTTTGAAGGAC>line_142ATGGTCTCGCCCAAACGGAGACTAATCGACGATTCCATTGCTCCCTCAGCTGCCGATCTCCAGCGGACATACGACGGACATGATGGGCCTCACGTTTTCGGAACACCCGGCAATCAAGTCTACATTCGGGGGCAGAACGATGGCGTATACAAGGTGCCAGGAGTAGGTGGTCAGTTCCATCACGCATCGTCGCCAGCAGATCACGTCTATACGGATGAGCAGGGGATCACGTATGTGCACAAAAAAGACGCTGGAGGACCAGGAACACATACGCTTAGAGGACCAGGTCTAGCTGCTGTACAGCCCGCCTACTCCACTGTGCAGCCTGCGGGACTTCGCAATCCCCAGTTTCATGTGGAGCGAAGTGGTCGCACTGTGGATGTTGGTTCTGGAGGATTTGTCGTCCAAAGGAGCAGACGTAGTCCTCAGTTCCATGTGGAGCGTCCTGGTCGCACTGTGGATGTGGGTTCCGGAGGATTTTTTGTCCAAAGGGGCAGGCGTAGTCCCCAGTTTCCTGTGGAGCGACCTTGGCAAAGGGTCGACGTCGGCTTTGAAGGAC>line_153ATGGTCTCGCCCAAACGGAGACTAATCGACGATTCCATTGCTCCCTCAGCTGCCGATCTCCAGCGGACATACGACGGACATGATGGGCCTCACGTTTTCGGAACACCCGGCAATCAAGTCTACATTCGGGGGCAGAACGATGGCGTATACAAGGTGCCAGGAGTAGGTGGTCAGTTCCATCACGCATCGTCGCCAGCAGATCACGTCTATACGGATGAGCAGGGGATCACGTATGTGCACAAAAAAGACGCTGGAGGACCAGGAACACATACGCTTAGAGGACCAGGTCTAGCTGCTGTACAGCCCGCCTACTCCACTGTGCAGCCTGCGGGACTTCGCAATCCCCAGTTTCATGTGGAGCGAAGTGGTCGCACTGTGGATGTTGGTTCTGGAGGATTTGTCGTCCAAAGGAGCAGACGTAGTCCTCAGTTCCATGTGGAGCGTCCTGGTCGCACTGTGGATGTGGGTTCCGGAGGATTTTTTGTCCAAAGGGGCAGGCGTAGTCCCCAGTTTCCTGTGGAGCGACCTTGGCAAAGGGTCGACGTCGGCTTTGAAGGAC>line_161ATGGTCTCGCCCAAACGGAGACTAATCGACGATTCCATTGCTCCCTCAGCTGCCGATCTCCAGCGGACATACGACGGACATGATGGGCCTCACGTTTTCGGAACACCCGGCAATCAAGTCTACATTCGGGGGCAGAACGATGGCGTATACAAGGTGCCAGGAGTAGGTGGTCAGTTCCATCACGCATCGTCGCCAGCAGATCACGTCTATACGGATGAGCAGGGGATCACGTATGTGCACAAAAAAGACGCTGGAGGACCAGGAACACATACGCTTAGAGGACCAGGTCTAGCTGCTGTACAGCCCGCCTACTCCACTGTGCAGCCTGCGGGACTTCGCAATCCCCAGTTTCATGTGGAGCGAAGTGGTCGCACTGTGGATGTTGGTTCTGGAGGATTTGTCGTCCAAAGGAGCAGACGTAGTCCTCAGTTCCATGTGGAGCGTCCTGGTCGCACTGTGGATGTGGGTTCCGGAGGATTTTTTGTCCAAAGGGGCAGGCGTAGTCCCCAGTTTCCTGTGGAGCGACCTTGGCAAAGGGTCGACGTCGGCTTTGAAGGAC>line_176ATGGTCTCGCCCAAACGGAGACTAATCGACGATTCCATTGCTCCCTCAGCTGCCGATCTCCAGCGGACATACGACGGACATGATGGGCCTCACGTTTTCGGAACACCCGGCAATCAAGTCTACATTCGGGGGCAGAACGATGGCGTATACAAGGTGCCAGGAGTAGGTGGTCAGTTCCATCACGCATCGTCGCCAGCAGATCACGTCTATACGGATGAGCAGGGGATCACGTATGTGCACAAAAAAGACGCTGGAGGACCAGGAACACATACGCTTAGAGGACCAGGTCTAGCTGCTGTACAGCCCGCCTACTCCACTGTGCAGCCTGCGGGACTTCGCAATCCCCAGTTTCATGTGGAGCGAAGTGGTCGCACTGTGGATGTTGGTTCTGGAGGATTTGTCGTCCAAAGGAGCAGACGTAGTCCTCAGTTCCATGTGGAGCGTCCTGGTCGCACTGTGGATGTGGGTTCCGGAGGATTTTTTGTCCAAAGGGGCAGGCGTAGTCCCCAGTTTCCTGTGGAGCGACCTTGGCAAAGGGTCGACGTCGGCTTTGAAGGAC>line_189ATGGTCTCGCCCAAACGGAGACTAATCGACGATTCCATTGCTCCCTCAGCTGCCGATCTCCAGCGGACATACGACGGACATGATGGGCCTCACGTTTTCGGAACACCCGGCAATCAAGTCTACATTCGGGGGCAGAACGATGGCGTATACAAGGTGCCAGGAGTAGGTGGTCAGTTCCATCACGCATCGTCGCCAGCAGATCACGTCTATACGGATGAGCAGGGGATCACGTATGTGCACAAAAAAGACGCTGGAGGACCAGGAACACATACGCTTAGAGGACCAGGTCTAGCTGCTGTACAGCCCGCCTACTCCACTGTGCAGCCTGCGGGACTTCGCAATCCCCAGTTTCATGTGGAGCGAAGTGGTCGCACTGTGGATGTTGGTTCTGGAGGATTTGTCGTCCAAAGGAGCAGACGTAGTCCTCAGTTCCATGTGGAGCGTCCTGGTCGCACTGTGGATGTGGGTTCCGGAGGATTTTTTGTCCAAAGGGGCAGGCGTAGTCCCCAGTTTCCTGTGGAGCGACCTTGGCAAAGGGTCGACGTCGGCTTTGAAGGAC>line_208ATGGTCTCGCCCAAACGGAGACTAATCGACGATTCCATTGCTCCCTCAGCTGCCGATCTCCAGCGGACATACGACGGACATGATGGGCCTCACGTTTTCGGAACACCCGGCAATCAAGTCTACATTCGGGGGCAGAACGATGGCGTATACAAGGTGCCAGGAGTAGGTGGTCAGTTCCATCACGCATCGTCGCCAGCAGATCACGTCTATACGGATGAGCAGGGGATCACGTATGTGCACAAAAAAGACGCTGGAGGACCAGGAACACATACGCTTAGAGGACCAGGTCTAGCTGCTGTACAGCCCGCCTACTCCACTGTGCAGCCTGCGGGACTTCGCAATCCCCAGTTTCATGTGGAGCGAAGTGGTCGCACTGTGGATGTTGGTTCTGGAGGATTTGTCGTCCAAAGGAGCAGACGTAGTCCTCAGTTCCATGTGGAGCGTCCTGGTCGCACTGTGGATGTGGGTTCCGGAGGATTTTTTGTCCAAAGGGGCAGGCGTAGTCCCCAGTTTCCTGTGGAGCGACCTTGGCAAAGGGTCGACGTCGGCTTTGAAGGAC>line_223ATGGTCTCGCCCAAACGGAGACTAATCGACGATTCCATTGCTCCCTCAGCTGCCGATCTCCAGCGGACATACGACGGACATGATGGGCCTCACGTTTTCGGAACACCCGGCAATCAAGTCTACATTCGGGGGCAGAACGATGGCGTATACAAGGTGCCAGGAGTAGGTGGTCAGTTCCATCACGCATCGTCGCCAGCAGATCACGTCTATACGGATGAGCAGGGGATCACGTATGTGCACAAAAAAGACGCTGGAGGACCAGGAACACATACGCTTAGAGGACCAGGTCTAGCTGCTGTACAGCCCGCCTACTCCACTGTGCAGCCTGCGGGACTTCGCAATCCCCAGTTTCATGTGGAGCGAAGTGGTCGCACTGTGGATGTTGGTTCTGGAGGATTTGTCGTCCAAAGGAGCAGACGTAGTCCTCAGTTCCATGTGGAGCGTCCTGGTCGCACTGTGGATGTGGGTTCCGGAGGATTTTTTGTCCAAAGGGGCAGGCGTAGTCCCCAGTTTCCTGTGGAGCGACCTTGGCAAAGGGTCGACGTCGGCTTTGAAGGAC>line_227ATGGTCTCGCCCAAACGGAGACTAATCGACGATTCCATTGCTCCCTCAGCTGCCGATCTCCAGCGGACATACGACGGACATGATGGGCCTCACGTTTTCGGAACACCCGGCAATCAAGTCTACATTCGGGGGCAGAACGATGGCGTATACAAGGTGCCAGGAGTAGGTGGTCAGTTCCATCACGCATCGTCGCCAGCAGATCACGTCTATACGGATGAGCAGGGGATCACGTATGTGCACAAAAAAGACGCTGGAGGACCAGGAACACATACGCTTAGAGGACCAGGTCTAGCTGCTGTACAGCCCGCCTACTCCACTGTGCAGCCTGCGGGACTTCGCAATCCCCAGTTTCATGTGGAGCGAAGTGGTCGCACTGTGGATGTTGGTTCTGGAGGATTTGTCGTCCAAAGGAGCAGACGTAGTCCTCAGTTCCATGTGGAGCGTCCTGGTCGCACTGTGGATGTGGGTTCCGGAGGATTTTTTGTCCAAAGGGGCAGGCGTAGTCCCCAGTTTCCTGTGGAGCGACCTTGGCAAAGGGTCGACGTCGGCTTTGAAGGAC>line_256ATGGTCTCGCCCAAACGGAGACTAATCGACGATTCCATTGCTCCCTCAGCTGCCGATCTCCAGCGGACATACGACGGACATGATGGGCCTCACGTTTTCGGAACACCCGGCAATCAAGTCTACATTCGGGGGCAGAACGATGGCGTATACAAGGTGCCAGGAGTAGGTGGTCAGTTCCATCACGCATCGTCGCCAGCAGATCACGTCTATACGGATGAGCAGGGGATCACGTATGTGCACAAAAAAGACGCTGGAGGACCAGGAACACATACGCTTAGAGGACCAGGTCTAGCTGCTGTACAGCCCGCCTACTCCACTGTGCAGCCTGCGGGACTTCGCAATCCCCAGTTTCATGTGGAGCGAAGTGGTCGCACTGTGGATGTTGGTTCTGGAGGATTTGTCGTCCAAAGGAGCAGACGTAGTCCTCAGTTCCATGTGGAGCGTCCTGGTCGCACTGTGGATGTGGGTTCCGGAGGATTTTTTGTCCAAAGGGGCAGGCGTAGTCCCCAGTTTCCTGTGGAGCGACCTTGGCAAAGGGTCGACGTCGGCTTTGAAGGAC>line_280ATGGTCTCGCCCAAACGGAGACTAATCGACGATTCCATTGCTCCCTCAGCTGCCGATCTCCAGCGGACATACGACGGACATGATGGGCCTCACGTTTTCGGAACACCCGGCAATCAAGTCTACATTCGGGGGCAGAACGATGGCGTATACAAGGTGCCAGGAGTAGGTGGTCAGTTCCATCACGCATCGTCGCCAGCAGATCACGTCTATACGGATGAGCAGGGGATCACGTATGTGCACAAAAAAGACGCTGGAGGACCAGGAACACATACGCTTAGAGGACCAGGTCTAGCTGCTGTACAGCCCGCCTACTCCACTGTGCAGCCTGCGGGACTTCGCAATCCCCAGTTTCATGTGGAGCGAAGTGGTCGCACTGTGGATGTTGGTTCTGGAGGATTTGTCGTCCAAAGGAGCAGACGTAGTCCTCAGTTCCATGTGGAGCGTCCTGGTCGCACTGTGGATGTGGGTTCCGGAGGATTTTTTGTCCAAAGGGGCAGGCGTAGTCCCCAGTTTCCTGTGGAGCGACCTTGGCAAAGGGTCGACGTCGGCTTTGAAGGAC>line_287ATGGTCTCGCCCAAACGGAGACTAATCGACGATTCCATTGCTCCCTCAGCTGCCGATCTCCAGCGGACATACGACGGACATGATGGGCCTCACGTTTTCGGAACACCCGGCAATCAAGTCTACATTCGGGGGCAGAACGATGGCGTATACAAGGTGCCAGGAGTAGGTGGTCAGTTCCATCACGCATCGTCGCCAGCAGATCACGTCTATACGGATGAGCAGGGGATCACGTATGTGCACAAAAAAGACGCTGGAGGACCAGGAACACATACGCTTAGAGGACCAGGTCTAGCTGCTGTACAGCCCGCCTACTCCACTGTGCAGCCTGCGGGACTTCGCAATCCCCAGTTTCATGTGGAGCGAAGTGGTCGCACTGTGGATGTTGGTTCTGGAGGATTTGTCGTCCAAAGGAGCAGACGTAGTCCTCAGTTCCATGTGGAGCGTCCTGGTCGCACTGTGGATGTGGGTTCCGGAGGATTTTTTGTCCAAAGGGGCAGGCGTAGTCCCCAGTTTCCTGTGGAGCGACCTTGGCAAAGGGTCGACGTCGGCTTTGAAGGAC>line_303ATGGTCTCGCCCAAACGGAGACTAATCGACGATTCCATTGCTCCCTCAGCTGCCGATCTCCAGCGGACATACGACGGACATGATGGGCCTCACGTTTTCGGAACACCCGGCAATCAAGTCTACATTCGGGGGCAGAACGATGGCGTATACAAGGTGCCAGGAGTAGGTGGTCAGTTCCATCACGCATCGTCGCCAGCAGATCACGTCTATACGGATGAGCAGGGGATCACGTATGTGCACAAAAAAGACGCTGGAGGACCAGGAACACATACGCTTAGAGGACCAGGTCTAGCTGCTGTACAGCCCGCCTACTCCACTGTGCAGCCTGCGGGACTTCGCAATCCCCAGTTTCATGTGGAGCGAAGTGGTCGCACTGTGGATGTTGGTTCTGGAGGATTTGTCGTCCAAAGGAGCAGACGTAGTCCTCAGTTCCATGTGGAGCGTCCTGGTCGCACTGTGGATGTGGGTTCCGGAGGATTTTTTGTCCAAAGGGGCAGGCGTAGTCCCCAGTTTCCTGTGGAGCGACCTTGGCAAAGGGTCGACGTCGGCTTTGAAGGAC>line_307ATGGTCTCGCCCAAACGGAGACTAATCGACGATTCCATTGCTCCCTCAGCTGCCGATCTCCAGCGGACATACGACGGACATGATGGGCCTCACGTTTTCGGAACACCCGGCAATCAAGTCTACATTCGGGGGCAGAACGATGGCGTATACAAGGTGCCAGGAGTAGGTGGTCAGTTCCATCACGCATCGTCGCCAGCAGATCACGTCTATACGGATGAGCAGGGGATCACGTATGTGCACAAAAAAGACGCTGGAGGACCAGGAACACATACGCTTAGAGGACCAGGTCTAGCTGCTGTACAGCCCGCCTACTCCACTGTGCAGCCTGCGGGACTTCGCAATCCCCAGTTTCATGTGGAGCGAAGTGGTCGCACTGTGGATGTTGGTTCTGGAGGATTTGTCGTCCAAAGGAGCAGACGTAGTCCTCAGTTCCATGTGGAGCGTCCTGGTCGCACTGTGGATGTGGGTTCCGGAGGATTTTTTGTCCAAAGGGGCAGGCGTAGTCCCCAGTTTCCTGTGGAGCGACCTTGGCAAAGGGTCGACGTCGGCTTTGAAGGAC>line_313ATGGTCTCGCCCAAACGGAGACTAATCGACGATTCCATTGCTCCCTCAGCTGCCGATCTCCAGCGGACATACGACGGACATGATGGGCCTCACGTTTTCGGAACACCCGGCAATCAAGTCTACATTCGGGGGCAGAACGATGGCGTATACAAGGTGCCAGGAGTAGGTGGTCAGTTCCATCACGCATCGTCGCCAGCAGATCACGTCTATACGGATGAGCAGGGGATCACGTATGTGCACAAAAAAGACGCTGGAGGACCAGGAACACATACGCTTAGAGGACCAGGTCTAGCTGCTGTACAGCCCGCCTACTCCACTGTGCAGCCTGCGGGACTTCGCAATCCCCAGTTTCATGTGGAGCGAAGTGGTCGCACTGTGGATGTTGGTTCTGGAGGATTTGTCGTCCAAAGGAGCAGACGTAGTCCTCAGTTCCATGTGGAGCGTCCTGGTCGCACTGTGGATGTGGGTTCCGGAGGATTTTTTGTCCAAAGGGGCAGGCGTAGTCCCCAGTTTCCTGTGGAGCGACCTTGGCAAAGGGTCGACGTCGGCTTTGAAGGAC>line_318ATGGTCTCGCCCAAACGGAGACTAATCGACGATTCCATTGCTCCCTCAGCTGCCGATCTCCAGCGGACATACGACGGACATGATGGGCCTCACGTTTTCGGAACACCCGGCAATCAAGTCTACATTCGGGGGCAGAACGATGGCGTATACAAGGTGCCAGGAGTAGGTGGTCAGTTCCATCACGCATCGTCGCCAGCAGATCACGTCTATACGGATGAGCAGGGGATCACGTATGTGCACAAAAAAGACGCTGGAGGACCAGGAACACATACGCTTAGAGGACCAGGTCTAGCTGCTGTACAGCCCGCCTACTCCACTGTGCAGCCTGCGGGACTTCGCAATCCCCAGTTTCATGTGGAGCGAAGTGGTCGCACTGTGGATGTTGGTTCTGGAGGATTTGTCGTCCAAAGGAGCAGACGTAGTCCTCAGTTCCATGTGGAGCGTCCTGGTCGCACTGTGGATGTGGGTTCCGGAGGATTTTTTGTCCAAAGGGGCAGGCGTAGTCCCCAGTTTCCTGTGGAGCGACCTTGGCAAAGGGTCGACGTCGGCTTTGAAGGAC>line_335ATGGTCTCGCCCAAACGGAGACTAATCGACGATTCCATTGCTCCCTCAGCTGCCGATCTCCAGCGGACATACGACGGACATGATGGGCCTCACGTTTTCGGAACACCCGGCAATCAAGTCTACATTCGGGGGCAGAACGATGGCGTATACAAGGTGCCAGGAGTAGGTGGTCAGTTCCATCACGCATCGTCGCCAGCAGATCACGTCTATACGGATGAGCAGGGGATCACGTATGTGCACAAAAAAGACGCTGGAGGACCAGGAACACATACGCTTAGAGGACCAGGTCTAGCTGCTGTACAGCCCGCCTACTCCACTGTGCAGCCTGCGGGACTTCGCAATCCCCAGTTTCATGTGGAGCGAAGTGGTCGCACTGTGGATGTTGGTTCTGGAGGATTTGTCGTCCAAAGGAGCAGACGTAGTCCTCAGTTCCATGTGGAGCGTCCTGGTCGCACTGTGGATGTGGGTTCCGGAGGATTTTTTGTCCAAAGGGGCAGGCGTAGTCCCCAGTTTCCTGTGGAGCGACCTTGGCAAAGGGTCGACGTCGGCTTTGAAGGAC>line_354ATGGTCTCGCCCAAACGGAGACTAATCGACGATTCCATTGCTCCCTCAGCTGCCGATCTCCAGCGGACATACGACGGACATGATGGGCCTCACGTTTTCGGAACACCCGGCAATCAAGTCTACATTCGGGGGCAGAACGATGGCGTATACAAGGTGCCAGGAGTAGGTGGTCAGTTCCATCACGCATCGTCGCCAGCAGATCACGTCTATACGGATGAGCAGGGGATCACGTATGTGCACAAAAAAGACGCTGGAGGACCAGGAACACATACGCTTAGAGGACCAGGTCTAGCTGCTGTACAGCCCGCCTACTCCACTGTGCAGCCTGCGGGACTTCGCAATCCCCAGTTTCATGTGGAGCGAAGTGGTCGCACTGTGGATGTTGGTTCTGGAGGATTTGTCGTCCAAAGGAGCAGACGTAGTCCTCAGTTCCATGTGGAGCGTCCTGGTCGCACTGTGGATGTGGGTTCCGGAGGATTTTTTGTCCAAAGGGGCAGGCGTAGTCCCCAGTTTCCTGTGGAGCGACCTTGGCAAAGGGTCGACGTCGGCTTTGAAGGAC>line_355ATGGTCTCGCCCAAACGGAGACTAATCGACGATTCCATTGCTCCCTCAGCTGCCGATCTCCAGCGGACATACGACGGACATGATGGGCCTCACGTTTTCGGAACACCCGGCAATCAAGTCTACATTCGGGGGCAGAACGATGGCGTATACAAGGTGCCAGGAGTAGGTGGTCAGTTCCATCACGCATCGTCGCCAGCAGATCACGTCTATACGGATGAGCAGGGGATCACGTATGTGCACAAAAAAGACGCTGGAGGACCAGGAACACATACGCTTAGAGGACCAGGTCTAGCTGCTGTACAGCCCGCCTACTCCACTGTGCAGCCTGCGGGACTTCGCAATCCCCAGTTTCATGTGGAGCGAAGTGGTCGCACTGTGGATGTTGGTTCTGGAGGATTTGTCGTCCAAAGGAGCAGACGTAGTCCTCAGTTCCATGTGGAGCGTCCTGGTCGCACTGTGGATGTGGGTTCCGGAGGATTTTTTGTCCAAAGGGGCAGGCGTAGTCCCCAGTTTCCTGTGGAGCGACCTTGGCAAAGGGTCGACGTCGGCTTTGAAGGAC>line_356ATGGTCTCGCCCAAACGGAGACTAATCGACGATTCCATTGCTCCCTCAGCTGCCGATCTCCAGCGGACATACGACGGACATGATGGGCCTCACGTTTTCGGAACACCCGGCAATCAAGTCTACATTCGGGGGCAGAACGATGGCGTATACAAGGTGCCAGGAGTAGGTGGTCAGTTCCATCACGCATCGTCGCCAGCAGATCACGTCTATACGGATGAGCAGGGGATCACGTATGTGCACAAAAAAGACGCTGGAGGACCAGGAACACATACGCTTAGAGGACCAGGTCTAGCTGCTGTACAGCCCGCCTACTCCACTGTGCAGCCTGCGGGACTTCGCAATCCCCAGTTTCATGTGGAGCGAAGTGGTCGCACTGTGGATGTTGGTTCTGGAGGATTTGTCGTCCAAAGGAGCAGACGTAGTCCTCAGTTCCATGTGGAGCGTCCTGGTCGCACTGTGGATGTGGGTTCCGGAGGATTTTTTGTCCAAAGGGGCAGGCGTAGTCCCCAGTTTCCTGTGGAGCGACCTTGGCAAAGGGTCGACGTCGGCTTTGAAGGAC>line_359ATGGTCTCGCCCAAACGGAGACTAATCGACGATTCCATTGCTCCCTCAGCTGCCGATCTCCAGCGGACATACGACGGACATGATGGGCCTCACGTTTTCGGAACACCCGGCAATCAAGTCTACATTCGGGGGCAGAACGATGGCGTATACAAGGTGCCAGGAGTAGGTGGTCAGTTCCATCACGCATCGTCGCCAGCAGATCACGTCTATACGGATGAGCAGGGGATCACGTATGTGCACAAAAAAGACGCTGGAGGACCAGGAACACATACGCTTAGAGGACCAGGTCTAGCTGCTGTACAGCCCGCCTACTCCACTGTGCAGCCTGCGGGACTTCGCAATCCCCAGTTTCATGTGGAGCGAAGTGGTCGCACTGTGGATGTTGGTTCTGGAGGATTTGTCGTCCAAAGGAGCAGACGTAGTCCTCAGTTCCATGTGGAGCGTCCTGGTCGCACTGTGGATGTGGGTTCCGGAGGATTTTTTGTCCAAAGGGGCAGGCGTAGTCCCCAGTTTCCTGTGGAGCGACCTTGGCAAAGGGTCGACGTCGGCTTTGAAGGAC>line_362ATGGTCTCGCCCAAACGGAGACTAATCGACGATTCCATTGCTCCCTCAGCTGCCGATCTCCAGCGGACATACGACGGACATGATGGGCCTCACGTTTTCGGAACACCCGGCAATCAAGTCTACATTCGGGGGCAGAACGATGGCGTATACAAGGTGCCAGGAGTAGGTGGTCAGTTCCATCACGCATCGTCGCCAGCAGATCACGTCTATACGGATGAGCAGGGGATCACGTATGTGCACAAAAAAGACGCTGGAGGACCAGGAACACATACGCTTAGAGGACCAGGTCTAGCTGCTGTACAGCCCGCCTACTCCACTGTGCAGCCTGCGGGACTTCGCAATCCCCAGTTTCATGTGGAGCGAAGTGGTCGCACTGTGGATGTTGGTTCTGGAGGATTTGTCGTCCAAAGGAGCAGACGTAGTCCTCAGTTCCATGTGGAGCGTCCTGGTCGCACTGTGGATGTGGGTTCCGGAGGATTTTTTGTCCAAAGGGGCAGGCGTAGTCCCCAGTTTCCTGTGGAGCGACCTTGGCAAAGGGTCGACGTCGGCTTTGAAGGAC>line_370ATGGTCTCGCCCAAACGGAGACTAATCGACGATTCCATTGCTCCCTCAGCTGCCGATCTCCAGCGGACATACGACGGACATGATGGGCCTCACGTTTTCGGAACACCCGGCAATCAAGTCTACATTCGGGGGCAGAACGATGGCGTATACAAGGTGCCAGGAGTAGGTGGTCAGTTCCATCACGCATCGTCGCCAGCAGATCACGTCTATACGGATGAGCAGGGGATCACGTATGTGCACAAAAAAGACGCTGGAGGACCAGGAACACATACGCTTAGAGGACCAGGTCTAGCTGCTGTACAGCCCGCCTACTCCACTGTGCAGCCTGCGGGACTTCGCAATCCCCAGTTTCATGTGGAGCGAAGTGGTCGCACTGTGGATGTTGGTTCTGGAGGATTTGTCGTCCAAAGGAGCAGACGTAGTCCTCAGTTCCATGTGGAGCGTCCTGGTCGCACTGTGGATGTGGGTTCCGGAGGATTTTTTGTCCAAAGGGGCAGGCGTAGTCCCCAGTTTCCTGTGGAGCGACCTTGGCAAAGGGTCGACGTCGGCTTTGAAGGAC>line_374ATGGTCTCGCCCAAACGGAGACTAATCGACGATTCCATTGCTCCCTCAGCTGCCGATCTCCAGCGGACATACGACGGACATGATGGGCCTCACGTTTTCGGAACACCCGGCAATCAAGTCTACATTCGGGGGCAGAACGATGGCGTATACAAGGTGCCAGGAGTAGGTGGTCAGTTCCATCACGCATCGTCGCCAGCAGATCACGTCTATACGGATGAGCAGGGGATCACGTATGTGCACAAAAAAGACGCTGGAGGACCAGGAACACATACGCTTAGAGGACCAGGTCTAGCTGCTGTACAGCCCGCCTACTCCACTGTGCAGCCTGCGGGACTTCGCAATCCCCAGTTTCATGTGGAGCGAAGTGGTCGCACTGTGGATGTTGGTTCTGGAGGATTTGTCGTCCAAAGGAGCAGACGTAGTCCTCAGTTCCATGTGGAGCGTCCTGGTCGCACTGTGGATGTGGGTTCCGGAGGATTTTTTGTCCAAAGGGGCAGGCGTAGTCCCCAGTTTCCTGTGGAGCGACCTTGGCAAAGGGTCGACGTCGGCTTTGAAGGAC>line_379ATGGTCTCGCCCAAACGGAGACTAATCGACGATTCCATTGCTCCCTCAGCTGCCGATCTCCAGCGGACATACGACGGACATGATGGGCCTCACGTTTTCGGAACACCCGGCAATCAAGTCTACATTCGGGGGCAGAACGATGGCGTATACAAGGTGCCAGGAGTAGGTGGTCAGTTCCATCACGCATCGTCGCCAGCAGATCACGTCTATACGGATGAGCAGGGGATCACGTATGTGCACAAAAAAGACGCTGGAGGACCAGGAACACATACGCTTAGAGGACCAGGTCTAGCTGCTGTACAGCCCGCCTACTCCACTGTGCAGCCTGCGGGACTTCGCAATCCCCAGTTTCATGTGGAGCGAAGTGGTCGCACTGTGGATGTTGGTTCTGGAGGATTTGTCGTCCAAAGGAGCAGACGTAGTCCTCAGTTCCATGTGGAGCGTCCTGGTCGCACTGTGGATGTGGGTTCCGGAGGATTTTTTGTCCAAAGGGGCAGGCGTAGTCCCCAGTTTCCTGTGGAGCGACCTTGGCAAAGGGTCGACGTCGGCTTTGAAGGAC>line_380ATGGTCTCGCCCAAACGGAGACTAATCGACGATTCCATTGCTCCCTCAGCTGCCGATCTCCAGCGGACATACGACGGACATGATGGGCCTCACGTTTTCGGAACACCCGGCAATCAAGTCTACATTCGGGGGCAGAACGATGGCGTATACAAGGTGCCAGGAGTAGGTGGTCAGTTCCATCACGCATCGTCGCCAGCAGATCACGTCTATACGGATGAGCAGGGGATCACGTATGTGCACAAAAAAGACGCTGGAGGACCAGGAACACATACGCTTAGAGGACCAGGTCTAGCTGCTGTACAGCCCGCCTACTCCACTGTGCAGCCTGCGGGACTTCGCAATCCCCAGTTTCATGTGGAGCGAAGTGGTCGCACTGTGGATGTTGGTTCTGGAGGATTTGTCGTCCAAAGGAGCAGACGTAGTCCTCAGTTCCATGTGGAGCGTCCTGGTCGCACTGTGGATGTGGGTTCCGGAGGATTTTTTGTCCAAAGGGGCAGGCGTAGTCCCCAGTTTCCTGTGGAGCGACCTTGGCAAAGGGTCGACGTCGGCTTTGAAGGAC>line_381ATGGTCTCGCCCAAACGGAGACTAATCGACGATTCCATTGCTCCCTCAGCTGCCGATCTCCAGCGGACATACGACGGACATGATGGGCCTCACGTTTTCGGAACACCCGGCAATCAAGTCTACATTCGGGGGCAGAACGATGGCGTATACAAGGTGCCAGGAGTAGGTGGTCAGTTCCATCACGCATCGTCGCCAGCAGATCACGTCTATACGGATGAGCAGGGGATCACGTATGTGCACAAAAAAGACGCTGGAGGACCAGGAACACATACGCTTAGAGGACCAGGTCTAGCTGCTGTACAGCCCGCCTACTCCACTGTGCAGCCTGCGGGACTTCGCAATCCCCAGTTTCATGTGGAGCGAAGTGGTCGCACTGTGGATGTTGGTTCTGGAGGATTTGTCGTCCAAAGGAGCAGACGTAGTCCTCAGTTCCATGTGGAGCGTCCTGGTCGCACTGTGGATGTGGGTTCCGGAGGATTTTTTGTCCAAAGGGGCAGGCGTAGTCCCCAGTTTCCTGTGGAGCGACCTTGGCAAAGGGTCGACGTCGGCTTTGAAGGAC>line_385ATGGTCTCGCCCAAACGGAGACTAATCGACGATTCCATTGCTCCCTCAGCTGCCGATCTCCAGCGGACATACGACGGACATGATGGGCCTCACGTTTTCGGAACACCCGGCAATCAAGTCTACATTCGGGGGCAGAACGATGGCGTATACAAGGTGCCAGGAGTAGGTGGTCAGTTCCATCACGCATCGTCGCCAGCAGATCACGTCTATACGGATGAGCAGGGGATCACGTATGTGCACAAAAAAGACGCTGGAGGACCAGGAACACATACGCTTAGAGGACCAGGTCTAGCTGCTGTACAGCCCGCCTACTCCACTGTGCAGCCTGCGGGACTTCGCAATCCCCAGTTTCATGTGGAGCGAAGTGGTCGCACTGTGGATGTTGGTTCTGGAGGATTTGTCGTCCAAAGGAGCAGACGTAGTCCTCAGTTCCATGTGGAGCGTCCTGGTCGCACTGTGGATGTGGGTTCCGGAGGATTTTTTGTCCAAAGGGGCAGGCGTAGTCCCCAGTTTCCTGTGGAGCGACCTTGGCAAAGGGTCGACGTCGGCTTTGAAGGAC>line_391ATGGTCTCGCCCAAACGGAGACTAATCGACGATTCCATTGCTCCCTCAGCTGCCGATCTCCAGCGGACATACGACGGACATGATGGGCCTCACGTTTTCGGAACACCCGGCAATCAAGTCTACATTCGGGGGCAGAACGATGGCGTATACAAGGTGCCAGGAGTAGGTGGTCAGTTCCATCACGCATCGTCGCCAGCAGATCACGTCTATACGGATGAGCAGGGGATCACGTATGTGCACAAAAAAGACGCTGGAGGACCAGGAACACATACGCTTAGAGGACCAGGTCTAGCTGCTGTACAGCCCGCCTACTCCACTGTGCAGCCTGCGGGACTTCGCAATCCCCAGTTTCATGTGGAGCGAAGTGGTCGCACTGTGGATGTTGGTTCTGGAGGATTTGTCGTCCAAAGGAGCAGACGTAGTCCTCAGTTCCATGTGGAGCGTCCTGGTCGCACTGTGGATGTGGGTTCCGGAGGATTTTTTGTCCAAAGGGGCAGGCGTAGTCCCCAGTTTCCTGTGGAGCGACCTTGGCAAAGGGTCGACGTCGGCTTTGAAGGAC>line_427ATGGTCTCGCCCAAACGGAGACTAATCGACGATTCCATTGCTCCCTCAGCTGCCGATCTCCAGCGGACATACGACGGACATGATGGGCCTCACGTTTTCGGAACACCCGGCAATCAAGTCTACATTCGGGGGCAGAACGATGGCGTATACAAGGTGCCAGGAGTAGGTGGTCAGTTCCATCACGCATCGTCGCCAGCAGATCACGTCTATACGGATGAGCAGGGGATCACGTATGTGCACAAAAAAGACGCTGGAGGACCAGGAACACATACGCTTAGAGGACCAGGTCTAGCTGCTGTACAGCCCGCCTACTCCACTGTGCAGCCTGCGGGACTTCGCAATCCCCAGTTTCATGTGGAGCGAAGTGGTCGCACTGTGGATGTTGGTTCTGGAGGATTTGTCGTCCAAAGGAGCAGACGTAGTCCTCAGTTCCATGTGGAGCGTCCTGGTCGCACTGTGGATGTGGGTTCCGGAGGATTTTTTGTCCAAAGGGGCAGGCGTAGTCCCCAGTTTCCTGTGGAGCGACCTTGGCAAAGGGTCGACGTCGGCTTTGAAGGAC>line_439ATGGTCTCGCCCAAACGGAGACTAATCGACGATTCCATTGCTCCCTCAGCTGCCGATCTCCAGCGGACATACGACGGACATGATGGGCCTCACGTTTTCGGAACACCCGGCAATCAAGTCTACATTCGGGGGCAGAACGATGGCGTATACAAGGTGCCAGGAGTAGGTGGTCAGTTCCATCACGCATCGTCGCCAGCAGATCACGTCTATACGGATGAGCAGGGGATCACGTATGTGCACAAAAAAGACGCTGGAGGACCAGGAACACATACGCTTAGAGGACCAGGTCTAGCTGCTGTACAGCCCGCCTACTCCACTGTGCAGCCTGCGGGACTTCGCAATCCCCAGTTTCATGTGGAGCGAAGTGGTCGCACTGTGGATGTTGGTTCTGGAGGATTTGTCGTCCAAAGGAGCAGACGTAGTCCTCAGTTCCATGTGGAGCGTCCTGGTCGCACTGTGGATGTGGGTTCCGGAGGATTTTTTGTCCAAAGGGGCAGGCGTAGTCCCCAGTTTCCTGTGGAGCGACCTTGGCAAAGGGTCGACGTCGGCTTTGAAGGAC>line_441ATGGTCTCGCCCAAACGGAGACTAATCGACGATTCCATTGCTCCCTCAGCTGCCGATCTCCAGCGGACATACGACGGACATGATGGGCCTCACGTTTTCGGAACACCCGGCAATCAAGTCTACATTCGGGGGCAGAACGATGGCGTATACAAGGTGCCAGGAGTAGGTGGTCAGTTCCATCACGCATCGTCGCCAGCAGATCACGTCTATACGGATGAGCAGGGGATCACGTATGTGCACAAAAAAGACGCTGGAGGACCAGGAACACATACGCTTAGAGGACCAGGTCTAGCTGCTGTACAGCCCGCCTACTCCACTGTGCAGCCTGCGGGACTTCGCAATCCCCAGTTTCATGTGGAGCGAAGTGGTCGCACTGTGGATGTTGGTTCTGGAGGATTTGTCGTCCAAAGGAGCAGACGTAGTCCTCAGTTCCATGTGGAGCGTCCTGGTCGCACTGTGGATGTGGGTTCCGGAGGATTTTTTGTCCAAAGGGGCAGGCGTAGTCCCCAGTTTCCTGTGGAGCGACCTTGGCAAAGGGTCGACGTCGGCTTTGAAGGAC>line_491ATGGTCTCGCCCAAACGGAGACTAATCGACGATTCCATTGCTCCCTCAGCTGCCGATCTCCAGCGGACATACGACGGACATGATGGGCCTCACGTTTTCGGAACACCCGGCAATCAAGTCTACATTCGGGGGCAGAACGATGGCGTATACAAGGTGCCAGGAGTAGGTGGTCAGTTCCATCACGCATCGTCGCCAGCAGATCACGTCTATACGGATGAGCAGGGGATCACGTATGTGCACAAAAAAGACGCTGGAGGACCAGGAACACATACGCTTAGAGGACCAGGTCTAGCTGCTGTACAGCCCGCCTACTCCACTGTGCAGCCTGCGGGACTTCGCAATCCCCAGTTTCATGTGGAGCGAAGTGGTCGCACTGTGGATGTTGGTTCTGGAGGATTTGTCGTCCAAAGGAGCAGACGTAGTCCTCAGTTCCATGTGGAGCGTCCTGGTCGCACTGTGGATGTGGGTTCCGGAGGATTTTTTGTCCAAAGGGGCAGGCGTAGTCCCCAGTTTCCTGTGGAGCGACCTTGGCAAAGGGTCGACGTCGGCTTTGAAGGAC>line_508ATGGTCTCGCCCAAACGGAGACTAATCGACGATTCCATTGCTCCCTCAGCTGCCGATCTCCAGCGGACATACGACGGACATGATGGGCCTCACGTTTTCGGAACACCCGGCAATCAAGTCTACATTCGGGGGCAGAACGATGGCGTATACAAGGTGCCAGGAGTAGGTGGTCAGTTCCATCACGCATCGTCGCCAGCAGATCACGTCTATACGGATGAGCAGGGGATCACGTATGTGCACAAAAAAGACGCTGGAGGACCAGGAACACATACGCTTAGAGGACCAGGTCTAGCTGCTGTACAGCCCGCCTACTCCACTGTGCAGCCTGCGGGACTTCGCAATCCCCAGTTTCATGTGGAGCGAAGTGGTCGCACTGTGGATGTTGGTTCTGGAGGATTTGTCGTCCAAAGGAGCAGACGTAGTCCTCAGTTCCATGTGGAGCGTCCTGGTCGCACTGTGGATGTGGGTTCCGGAGGATTTTTTGTCCAAAGGGGCAGGCGTAGTCCCCAGTTTCCTGTGGAGCGACCTTGGCAAAGGGTCGACGTCGGCTTTGAAGGAC>line_535ATGGTCTCGCCCAAACGGAGACTAATCGACGATTCCATTGCTCCCTCAGCTGCCGATCTCCAGCGGACATACGACGGACATGATGGGCCTCACGTTTTCGGAACACCCGGCAATCAAGTCTACATTCGGGGGCAGAACGATGGCGTATACAAGGTGCCAGGAGTAGGTGGTCAGTTCCATCACGCATCGTCGCCAGCAGATCACGTCTATACGGATGAGCAGGGGATCACGTATGTGCACAAAAAAGACGCTGGAGGACCAGGAACACATACGCTTAGAGGACCAGGTCTAGCTGCTGTACAGCCCGCCTACTCCACTGTGCAGCCTGCGGGACTTCGCAATCCCCAGTTTCATGTGGAGCGAAGTGGTCGCACTGTGGATGTTGGTTCTGGAGGATTTGTCGTCCAAAGGAGCAGACGTAGTCCTCAGTTCCATGTGGAGCGTCCTGGTCGCACTGTGGATGTGGGTTCCGGAGGATTTTTTGTCCAAAGGGGCAGGCGTAGTCCCCAGTTTCCTGTGGAGCGACCTTGGCAAAGGGTCGACGTCGGCTTTGAAGGAC>line_551ATGGTCTCGCCCAAACGGAGACTAATCGACGATTCCATTGCTCCCTCAGCTGCCGATCTCCAGCGGACATACGACGGACATGATGGGCCTCACGTTTTCGGAACACCCGGCAATCAAGTCTACATTCGGGGGCAGAACGATGGCGTATACAAGGTGCCAGGAGTAGGTGGTCAGTTCCATCACGCATCGTCGCCAGCAGATCACGTCTATACGGATGAGCAGGGGATCACGTATGTGCACAAAAAAGACGCTGGAGGACCAGGAACACATACGCTTAGAGGACCAGGTCTAGCTGCTGTACAGCCCGCCTACTCCACTGTGCAGCCTGCGGGACTTCGCAATCCCCAGTTTCATGTGGAGCGAAGTGGTCGCACTGTGGATGTTGGTTCTGGAGGATTTGTCGTCCAAAGGAGCAGACGTAGTCCTCAGTTCCATGTGGAGCGTCCTGGTCGCACTGTGGATGTGGGTTCCGGAGGATTTTTTGTCCAAAGGGGCAGGCGTAGTCCCCAGTTTCCTGTGGAGCGACCTTGGCAAAGGGTCGACGTCGGCTTTGAAGGAC>line_559ATGGTCTCGCCCAAACGGAGACTAATCGACGATTCCATTGCTCCCTCAGCTGCCGATCTCCAGCGGACATACGACGGACATGATGGGCCTCACGTTTTCGGAACACCCGGCAATCAAGTCTACATTCGGGGGCAGAACGATGGCGTATACAAGGTGCCAGGAGTAGGTGGTCAGTTCCATCACGCATCGTCGCCAGCAGATCACGTCTATACGGATGAGCAGGGGATCACGTATGTGCACAAAAAAGACGCTGGAGGACCAGGAACACATACGCTTAGAGGACCAGGTCTAGCTGCTGTACAGCCCGCCTACTCCACTGTGCAGCCTGCGGGACTTCGCAATCCCCAGTTTCATGTGGAGCGAAGTGGTCGCACTGTGGATGTTGGTTCTGGAGGATTTGTCGTCCAAAGGAGCAGACGTAGTCCTCAGTTCCATGTGGAGCGTCCTGGTCGCACTGTGGATGTGGGTTCCGGAGGATTTTTTGTCCAAAGGGGCAGGCGTAGTCCCCAGTTTCCTGTGGAGCGACCTTGGCAAAGGGTCGACGTCGGCTTTGAAGGAC>line_584ATGGTCTCGCCCAAACGGAGACTAATCGACGATTCCATTGCTCCCTCAGCTGCCGATCTCCAGCGGACATACGACGGACATGATGGGCCTCACGTTTTCGGAACACCCGGCAATCAAGTCTACATTCGGGGGCAGAACGATGGCGTATACAAGGTGCCAGGAGTAGGTGGTCAGTTCCATCACGCATCGTCGCCAGCAGATCACGTCTATACGGATGAGCAGGGGATCACGTATGTGCACAAAAAAGACGCTGGAGGACCAGGAACACATACGCTTAGAGGACCAGGTCTAGCTGCTGTACAGCCCGCCTACTCCACTGTGCAGCCTGCGGGACTTCGCAATCCCCAGTTTCATGTGGAGCGAAGTGGTCGCACTGTGGATGTTGGTTCTGGAGGATTTGTCGTCCAAAGGAGCAGACGTAGTCCTCAGTTCCATGTGGAGCGTCCTGGTCGCACTGTGGATGTGGGTTCCGGAGGATTTTTTGTCCAAAGGGGCAGGCGTAGTCCCCAGTTTCCTGTGGAGCGACCTTGGCAAAGGGTCGACGTCGGCTTTGAAGGAC>line_589ATGGTCTCGCCCAAACGGAGACTAATCGACGATTCCATTGCTCCCTCAGCTGCCGATCTCCAGCGGACATACGACGGACATGATGGGCCTCACGTTTTCGGAACACCCGGCAATCAAGTCTACATTCGGGGGCAGAACGATGGCGTATACAAGGTGCCAGGAGTAGGTGGTCAGTTCCATCACGCATCGTCGCCAGCAGATCACGTCTATACGGATGAGCAGGGGATCACGTATGTGCACAAAAAAGACGCTGGAGGACCAGGAACACATACGCTTAGAGGACCAGGTCTAGCTGCTGTACAGCCCGCCTACTCCACTGTGCAGCCTGCGGGACTTCGCAATCCCCAGTTTCATGTGGAGCGAAGTGGTCGCACTGTGGATGTTGGTTCTGGAGGATTTGTCGTCCAAAGGAGCAGACGTAGTCCTCAGTTCCATGTGGAGCGTCCTGGTCGCACTGTGGATGTGGGTTCCGGAGGATTTTTTGTCCAAAGGGGCAGGCGTAGTCCCCAGTTTCCTGTGGAGCGACCTTGGCAAAGGGTCGACGTCGGCTTTGAAGGAC>line_595ATGGTCTCGCCCAAACGGAGACTAATCGACGATTCCATTGCTCCCTCAGCTGCCGATCTCCAGCGGACATACGACGGACATGATGGGCCTCACGTTTTCGGAACACCCGGCAATCAAGTCTACATTCGGGGGCAGAACGATGGCGTATACAAGGTGCCAGGAGTAGGTGGTCAGTTCCATCACGCATCGTCGCCAGCAGATCACGTCTATACGGATGAGCAGGGGATCACGTATGTGCACAAAAAAGACGCTGGAGGACCAGGAACACATACGCTTAGAGGACCAGGTCTAGCTGCTGTACAGCCCGCCTACTCCACTGTGCAGCCTGCGGGACTTCGCAATCCCCAGTTTCATGTGGAGCGAAGTGGTCGCACTGTGGATGTTGGTTCTGGAGGATTTGTCGTCCAAAGGAGCAGACGTAGTCCTCAGTTCCATGTGGAGCGTCCTGGTCGCACTGTGGATGTGGGTTCCGGAGGATTTTTTGTCCAAAGGGGCAGGCGTAGTCCCCAGTTTCCTGTGGAGCGACCTTGGCAAAGGGTCGACGTCGGCTTTGAAGGAC>line_639ATGGTCTCGCCCAAACGGAGACTAATCGACGATTCCATTGCTCCCTCAGCTGCCGATCTCCAGCGGACATACGACGGACATGATGGGCCTCACGTTTTCGGAACACCCGGCAATCAAGTCTACATTCGGGGGCAGAACGATGGCGTATACAAGGTGCCAGGAGTAGGTGGTCAGTTCCATCACGCATCGTCGCCAGCAGATCACGTCTATACGGATGAGCAGGGGATCACGTATGTGCACAAAAAAGACGCTGGAGGACCAGGAACACATACGCTTAGAGGACCAGGTCTAGCTGCTGTACAGCCCGCCTACTCCACTGTGCAGCCTGCGGGACTTCGCAATCCCCAGTTTCATGTGGAGCGAAGTGGTCGCACTGTGGATGTTGGTTCTGGAGGATTTGTCGTCCAAAGGAGCAGACGTAGTCCTCAGTTCCATGTGGAGCGTCCTGGTCGCACTGTGGATGTGGGTTCCGGAGGATTTTTTGTCCAAAGGGGCAGGCGTAGTCCCCAGTTTCCTGTGGAGCGACCTTGGCAAAGGGTCGACGTCGGCTTTGAAGGAC>line_705ATGGTCTCGCCCAAACGGAGACTAATCGACGATTCCATTGCTCCCTCAGCTGCCGATCTCCAGCGGACATACGACGGACATGATGGGCCTCACGTTTTCGGAACACCCGGCAATCAAGTCTACATTCGGGGGCAGAACGATGGCGTATACAAGGTGCCAGGAGTAGGTGGTCAGTTCCATCACGCATCGTCGCCAGCAGATCACGTCTATACGGATGAGCAGGGGATCACGTATGTGCACAAAAAAGACGCTGGAGGACCAGGAACACATACGCTTAGAGGACCAGGTCTAGCTGCTGTACAGCCCGCCTACTCCACTGTGCAGCCTGCGGGACTTCGCAATCCCCAGTTTCATGTGGAGCGAAGTGGTCGCACTGTGGATGTTGGTTCTGGAGGATTTGTCGTCCAAAGGAGCAGACGTAGTCCTCAGTTCCATGTGGAGCGTCCTGGTCGCACTGTGGATGTGGGTTCCGGAGGATTTTTTGTCCAAAGGGGCAGGCGTAGTCCCCAGTTTCCTGTGGAGCGACCTTGGCAAAGGGTCGACGTCGGCTTTGAAGGAC>line_716ATGGTCTCGCCCAAACGGAGACTAATCGACGATTCCATTGCTCCCTCAGCTGCCGATCTCCAGCGGACATACGACGGACATGATGGGCCTCACGTTTTCGGAACACCCGGCAATCAAGTCTACATTCGGGGGCAGAACGATGGCGTATACAAGGTGCCAGGAGTAGGTGGTCAGTTCCATCACGCATCGTCGCCAGCAGATCACGTCTATACGGATGAGCAGGGGATCACGTATGTGCACAAAAAAGACGCTGGAGGACCAGGAACACATACGCTTAGAGGACCAGGTCTAGCTGCTGTACAGCCCGCCTACTCCACTGTGCAGCCTGCGGGACTTCGCAATCCCCAGTTTCATGTGGAGCGAAGTGGTCGCACTGTGGATGTTGGTTCTGGAGGATTTGTCGTCCAAAGGAGCAGACGTAGTCCTCAGTTCCATGTGGAGCGTCCTGGTCGCACTGTGGATGTGGGTTCCGGAGGATTTTTTGTCCAAAGGGGCAGGCGTAGTCCCCAGTTTCCTGTGGAGCGACCTTGGCAAAGGGTCGACGTCGGCTTTGAAGGAC>line_721ATGGTCTCGCCCAAACGGAGACTAATCGACGATTCCATTGCTCCCTCAGCTGCCGATCTCCAGCGGACATACGACGGACATGATGGGCCTCACGTTTTCGGAACACCCGGCAATCAAGTCTACATTCGGGGGCAGAACGATGGCGTATACAAGGTGCCAGGAGTAGGTGGTCAGTTCCATCACGCATCGTCGCCAGCAGATCACGTCTATACGGATGAGCAGGGGATCACGTATGTGCACAAAAAAGACGCTGGAGGACCAGGAACACATACGCTTAGAGGACCAGGTCTAGCTGCTGTACAGCCCGCCTACTCCACTGTGCAGCCTGCGGGACTTCGCAATCCCCAGTTTCATGTGGAGCGAAGTGGTCGCACTGTGGATGTTGGTTCTGGAGGATTTGTCGTCCAAAGGAGCAGACGTAGTCCTCAGTTCCATGTGGAGCGTCCTGGTCGCACTGTGGATGTGGGTTCCGGAGGATTTTTTGTCCAAAGGGGCAGGCGTAGTCCCCAGTTTCCTGTGGAGCGACCTTGGCAAAGGGTCGACGTCGGCTTTGAAGGAC>line_737ATGGTCTCGCCCAAACGGAGACTAATCGACGATTCCATTGCTCCCTCAGCTGCCGATCTCCAGCGGACATACGACGGACATGATGGGCCTCACGTTTTCGGAACACCCGGCAATCAAGTCTACATTCGGGGGCAGAACGATGGCGTATACAAGGTGCCAGGAGTAGGTGGTCAGTTCCATCACGCATCGTCGCCAGCAGATCACGTCTATACGGATGAGCAGGGGATCACGTATGTGCACAAAAAAGACGCTGGAGGACCAGGAACACATACGCTTAGAGGACCAGGTCTAGCTGCTGTACAGCCCGCCTACTCCACTGTGCAGCCTGCGGGACTTCGCAATCCCCAGTTTCATGTGGAGCGAAGTGGTCGCACTGTGGATGTTGGTTCTGGAGGATTTGTCGTCCAAAGGAGCAGACGTAGTCCTCAGTTCCATGTGGAGCGTCCTGGTCGCACTGTGGATGTGGGTTCCGGAGGATTTTTTGTCCAAAGGGGCAGGCGTAGTCCCCAGTTTCCTGTGGAGCGACCTTGGCAAAGGGTCGACGTCGGCTTTGAAGGAC>line_738ATGGTCTCGCCCAAACGGAGACTAATCGACGATTCCATTGCTCCCTCAGCTGCCGATCTCCAGCGGACATACGACGGACATGATGGGCCTCACGTTTTCGGAACACCCGGCAATCAAGTCTACATTCGGGGGCAGAACGATGGCGTATACAAGGTGCCAGGAGTAGGTGGTCAGTTCCATCACGCATCGTCGCCAGCAGATCACGTCTATACGGATGAGCAGGGGATCACGTATGTGCACAAAAAAGACGCTGGAGGACCAGGAACACATACGCTTAGAGGACCAGGTCTAGCTGCTGTACAGCCCGCCTACTCCACTGTGCAGCCTGCGGGACTTCGCAATCCCCAGTTTCATGTGGAGCGAAGTGGTCGCACTGTGGATGTTGGTTCTGGAGGATTTGTCGTCCAAAGGAGCAGACGTAGTCCTCAGTTCCATGTGGAGCGTCCTGGTCGCACTGTGGATGTGGGTTCCGGAGGATTTTTTGTCCAAAGGGGCAGGCGTAGTCCCCAGTTTCCTGTGGAGCGACCTTGGCAAAGGGTCGACGTCGGCTTTGAAGGAC>line_748ATGGTCTCGCCCAAACGGAGACTAATCGACGATTCCATTGCTCCCTCAGCTGCCGATCTCCAGCGGACATACGACGGACATGATGGGCCTCACGTTTTCGGAACACCCGGCAATCAAGTCTACATTCGGGGGCAGAACGATGGCGTATACAAGGTGCCAGGAGTAGGTGGTCAGTTCCATCACGCATCGTCGCCAGCAGATCACGTCTATACGGATGAGCAGGGGATCACGTATGTGCACAAAAAAGACGCTGGAGGACCAGGAACACATACGCTTAGAGGACCAGGTCTAGCTGCTGTACAGCCCGCCTACTCCACTGTGCAGCCTGCGGGACTTCGCAATCCCCAGTTTCATGTGGAGCGAAGTGGTCGCACTGTGGATGTTGGTTCTGGAGGATTTGTCGTCCAAAGGAGCAGACGTAGTCCTCAGTTCCATGTGGAGCGTCCTGGTCGCACTGTGGATGTGGGTTCCGGAGGATTTTTTGTCCAAAGGGGCAGGCGTAGTCCCCAGTTTCCTGTGGAGCGACCTTGGCAAAGGGTCGACGTCGGCTTTGAAGGAC>line_765ATGGTCTCGCCCAAACGGAGACTAATCGACGATTCCATTGCTCCCTCAGCTGCCGATCTCCAGCGGACATACGACGGACATGATGGGCCTCACGTTTTCGGAACACCCGGCAATCAAGTCTACATTCGGGGGCAGAACGATGGCGTATACAAGGTGCCAGGAGTAGGTGGTCAGTTCCATCACGCATCGTCGCCAGCAGATCACGTCTATACGGATGAGCAGGGGATCACGTATGTGCACAAAAAAGACGCTGGAGGACCAGGAACACATACGCTTAGAGGACCAGGTCTAGCTGCTGTACAGCCCGCCTACTCCACTGTGCAGCCTGCGGGACTTCGCAATCCCCAGTTTCATGTGGAGCGAAGTGGTCGCACTGTGGATGTTGGTTCTGGAGGATTTGTCGTCCAAAGGAGCAGACGTAGTCCTCAGTTCCATGTGGAGCGTCCTGGTCGCACTGTGGATGTGGGTTCCGGAGGATTTTTTGTCCAAAGGGGCAGGCGTAGTCCCCAGTTTCCTGTGGAGCGACCTTGGCAAAGGGTCGACGTCGGCTTTGAAGGAC>line_774ATGGTCTCGCCCAAACGGAGACTAATCGACGATTCCATTGCTCCCTCAGCTGCCGATCTCCAGCGGACATACGACGGACATGATGGGCCTCACGTTTTCGGAACACCCGGCAATCAAGTCTACATTCGGGGGCAGAACGATGGCGTATACAAGGTGCCAGGAGTAGGTGGTCAGTTCCATCACGCATCGTCGCCAGCAGATCACGTCTATACGGATGAGCAGGGGATCACGTATGTGCACAAAAAAGACGCTGGAGGACCAGGAACACATACGCTTAGAGGACCAGGTCTAGCTGCTGTACAGCCCGCCTACTCCACTGTGCAGCCTGCGGGACTTCGCAATCCCCAGTTTCATGTGGAGCGAAGTGGTCGCACTGTGGATGTTGGTTCTGGAGGATTTGTCGTCCAAAGGAGCAGACGTAGTCCTCAGTTCCATGTGGAGCGTCCTGGTCGCACTGTGGATGTGGGTTCCGGAGGATTTTTTGTCCAAAGGGGCAGGCGTAGTCCCCAGTTTCCTGTGGAGCGACCTTGGCAAAGGGTCGACGTCGGCTTTGAAGGAC>line_783ATGGTCTCGCCCAAACGGAGACTAATCGACGATTCCATTGCTCCCTCAGCTGCCGATCTCCAGCGGACATACGACGGACATGATGGGCCTCACGTTTTCGGAACACCCGGCAATCAAGTCTACATTCGGGGGCAGAACGATGGCGTATACAAGGTGCCAGGAGTAGGTGGTCAGTTCCATCACGCATCGTCGCCAGCAGATCACGTCTATACGGATGAGCAGGGGATCACGTATGTGCACAAAAAAGACGCTGGAGGACCAGGAACACATACGCTTAGAGGACCAGGTCTAGCTGCTGTACAGCCCGCCTACTCCACTGTGCAGCCTGCGGGACTTCGCAATCCCCAGTTTCATGTGGAGCGAAGTGGTCGCACTGTGGATGTTGGTTCTGGAGGATTTGTCGTCCAAAGGAGCAGACGTAGTCCTCAGTTCCATGTGGAGCGTCCTGGTCGCACTGTGGATGTGGGTTCCGGAGGATTTTTTGTCCAAAGGGGCAGGCGTAGTCCCCAGTTTCCTGTGGAGCGACCTTGGCAAAGGGTCGACGTCGGCTTTGAAGGAC>line_790ATGGTCTCGCCCAAACGGAGACTAATCGACGATTCCATTGCTCCCTCAGCTGCCGATCTCCAGCGGACATACGACGGACATGATGGGCCTCACGTTTTCGGAACACCCGGCAATCAAGTCTACATTCGGGGGCAGAACGATGGCGTATACAAGGTGCCAGGAGTAGGTGGTCAGTTCCATCACGCATCGTCGCCAGCAGATCACGTCTATACGGATGAGCAGGGGATCACGTATGTGCACAAAAAAGACGCTGGAGGACCAGGAACACATACGCTTAGAGGACCAGGTCTAGCTGCTGTACAGCCCGCCTACTCCACTGTGCAGCCTGCGGGACTTCGCAATCCCCAGTTTCATGTGGAGCGAAGTGGTCGCACTGTGGATGTTGGTTCTGGAGGATTTGTCGTCCAAAGGAGCAGACGTAGTCCTCAGTTCCATGTGGAGCGTCCTGGTCGCACTGTGGATGTGGGTTCCGGAGGATTTTTTGTCCAAAGGGGCAGGCGTAGTCCCCAGTTTCCTGTGGAGCGACCTTGGCAAAGGGTCGACGTCGGCTTTGAAGGAC>line_801ATGGTCTCGCCCAAACGGAGACTAATCGACGATTCCATTGCTCCCTCAGCTGCCGATCTCCAGCGGACATACGACGGACATGATGGGCCTCACGTTTTCGGAACACCCGGCAATCAAGTCTACATTCGGGGGCAGAACGATGGCGTATACAAGGTGCCAGGAGTAGGTGGTCAGTTCCATCACGCATCGTCGCCAGCAGATCACGTCTATACGGATGAGCAGGGGATCACGTATGTGCACAAAAAAGACGCTGGAGGACCAGGAACACATACGCTTAGAGGACCAGGTCTAGCTGCTGTACAGCCCGCCTACTCCACTGTGCAGCCTGCGGGACTTCGCAATCCCCAGTTTCATGTGGAGCGAAGTGGTCGCACTGTGGATGTTGGTTCTGGAGGATTTGTCGTCCAAAGGAGCAGACGTAGTCCTCAGTTCCATGTGGAGCGTCCTGGTCGCACTGTGGATGTGGGTTCCGGAGGATTTTTTGTCCAAAGGGGCAGGCGTAGTCCCCAGTTTCCTGTGGAGCGACCTTGGCAAAGGGTCGACGTCGGCTTTGAAGGAC>line_802ATGGTCTCGCCCAAACGGAGACTAATCGACGATTCCATTGCTCCCTCAGCTGCCGATCTCCAGCGGACATACGACGGACATGATGGGCCTCACGTTTTCGGAACACCCGGCAATCAAGTCTACATTCGGGGGCAGAACGATGGCGTATACAAGGTGCCAGGAGTAGGTGGTCAGTTCCATCACGCATCGTCGCCAGCAGATCACGTCTATACGGATGAGCAGGGGATCACGTATGTGCACAAAAAAGACGCTGGAGGACCAGGAACACATACGCTTAGAGGACCAGGTCTAGCTGCTGTACAGCCCGCCTACTCCACTGTGCAGCCTGCGGGACTTCGCAATCCCCAGTTTCATGTGGAGCGAAGTGGTCGCACTGTGGATGTTGGTTCTGGAGGATTTGTCGTCCAAAGGAGCAGACGTAGTCCTCAGTTCCATGTGGAGCGTCCTGGTCGCACTGTGGATGTGGGTTCCGGAGGATTTTTTGTCCAAAGGGGCAGGCGTAGTCCCCAGTTTCCTGTGGAGCGACCTTGGCAAAGGGTCGACGTCGGCTTTGAAGGAC>line_804ATGGTCTCGCCCAAACGGAGACTAATCGACGATTCCATTGCTCCCTCAGCTGCCGATCTCCAGCGGACATACGACGGACATGATGGGCCTCACGTTTTCGGAACACCCGGCAATCAAGTCTACATTCGGGGGCAGAACGATGGCGTATACAAGGTGCCAGGAGTAGGTGGTCAGTTCCATCACGCATCGTCGCCAGCAGATCACGTCTATACGGATGAGCAGGGGATCACGTATGTGCACAAAAAAGACGCTGGAGGACCAGGAACACATACGCTTAGAGGACCAGGTCTAGCTGCTGTACAGCCCGCCTACTCCACTGTGCAGCCTGCGGGACTTCGCAATCCCCAGTTTCATGTGGAGCGAAGTGGTCGCACTGTGGATGTTGGTTCTGGAGGATTTGTCGTCCAAAGGAGCAGACGTAGTCCTCAGTTCCATGTGGAGCGTCCTGGTCGCACTGTGGATGTGGGTTCCGGAGGATTTTTTGTCCAAAGGGGCAGGCGTAGTCCCCAGTTTCCTGTGGAGCGACCTTGGCAAAGGGTCGACGTCGGCTTTGAAGGAC>line_805ATGGTCTCGCCCAAACGGAGACTAATCGACGATTCCATTGCTCCCTCAGCTGCCGATCTCCAGCGGACATACGACGGACATGATGGGCCTCACGTTTTCGGAACACCCGGCAATCAAGTCTACATTCGGGGGCAGAACGATGGCGTATACAAGGTGCCAGGAGTAGGTGGTCAGTTCCATCACGCATCGTCGCCAGCAGATCACGTCTATACGGATGAGCAGGGGATCACGTATGTGCACAAAAAAGACGCTGGAGGACCAGGAACACATACGCTTAGAGGACCAGGTCTAGCTGCTGTACAGCCCGCCTACTCCACTGTGCAGCCTGCGGGACTTCGCAATCCCCAGTTTCATGTGGAGCGAAGTGGTCGCACTGTGGATGTTGGTTCTGGAGGATTTGTCGTCCAAAGGAGCAGACGTAGTCCTCAGTTCCATGTGGAGCGTCCTGGTCGCACTGTGGATGTGGGTTCCGGAGGATTTTTTGTCCAAAGGGGCAGGCGTAGTCCCCAGTTTCCTGTGGAGCGACCTTGGCAAAGGGTCGACGTCGGCTTTGAAGGAC>line_808ATGGTCTCGCCCAAACGGAGACTAATCGACGATTCCATTGCTCCCTCAGCTGCCGATCTCCAGCGGACATACGACGGACATGATGGGCCTCACGTTTTCGGAACACCCGGCAATCAAGTCTACATTCGGGGGCAGAACGATGGCGTATACAAGGTGCCAGGAGTAGGTGGTCAGTTCCATCACGCATCGTCGCCAGCAGATCACGTCTATACGGATGAGCAGGGGATCACGTATGTGCACAAAAAAGACGCTGGAGGACCAGGAACACATACGCTTAGAGGACCAGGTCTAGCTGCTGTACAGCCCGCCTACTCCACTGTGCAGCCTGCGGGACTTCGCAATCCCCAGTTTCATGTGGAGCGAAGTGGTCGCACTGTGGATGTTGGTTCTGGAGGATTTGTCGTCCAAAGGAGCAGACGTAGTCCTCAGTTCCATGTGGAGCGTCCTGGTCGCACTGTGGATGTGGGTTCCGGAGGATTTTTTGTCCAAAGGGGCAGGCGTAGTCCCCAGTTTCCTGTGGAGCGACCTTGGCAAAGGGTCGACGTCGGCTTTGAAGGAC>line_810ATGGTCTCGCCCAAACGGAGACTAATCGACGATTCCATTGCTCCCTCAGCTGCCGATCTCCAGCGGACATACGACGGACATGATGGGCCTCACGTTTTCGGAACACCCGGCAATCAAGTCTACATTCGGGGGCAGAACGATGGCGTATACAAGGTGCCAGGAGTAGGTGGTCAGTTCCATCACGCATCGTCGCCAGCAGATCACGTCTATACGGATGAGCAGGGGATCACGTATGTGCACAAAAAAGACGCTGGAGGACCAGGAACACATACGCTTAGAGGACCAGGTCTAGCTGCTGTACAGCCCGCCTACTCCACTGTGCAGCCTGCGGGACTTCGCAATCCCCAGTTTCATGTGGAGCGAAGTGGTCGCACTGTGGATGTTGGTTCTGGAGGATTTGTCGTCCAAAGGAGCAGACGTAGTCCTCAGTTCCATGTGGAGCGTCCTGGTCGCACTGTGGATGTGGGTTCCGGAGGATTTTTTGTCCAAAGGGGCAGGCGTAGTCCCCAGTTTCCTGTGGAGCGACCTTGGCAAAGGGTCGACGTCGGCTTTGAAGGAC>line_818ATGGTCTCGCCCAAACGGAGACTAATCGACGATTCCATTGCTCCCTCAGCTGCCGATCTCCAGCGGACATACGACGGACATGATGGGCCTCACGTTTTCGGAACACCCGGCAATCAAGTCTACATTCGGGGGCAGAACGATGGCGTATACAAGGTGCCAGGAGTAGGTGGTCAGTTCCATCACGCATCGTCGCCAGCAGATCACGTCTATACGGATGAGCAGGGGATCACGTATGTGCACAAAAAAGACGCTGGAGGACCAGGAACACATACGCTTAGAGGACCAGGTCTAGCTGCTGTACAGCCCGCCTACTCCACTGTGCAGCCTGCGGGACTTCGCAATCCCCAGTTTCATGTGGAGCGAAGTGGTCGCACTGTGGATGTTGGTTCTGGAGGATTTGTCGTCCAAAGGAGCAGACGTAGTCCTCAGTTCCATGTGGAGCGTCCTGGTCGCACTGTGGATGTGGGTTCCGGAGGATTTTTTGTCCAAAGGGGCAGGCGTAGTCCCCAGTTTCCTGTGGAGCGACCTTGGCAAAGGGTCGACGTCGGCTTTGAAGGAC>line_820ATGGTCTCGCCCAAACGGAGACTAATCGACGATTCCATTGCTCCCTCAGCTGCCGATCTCCAGCGGACATACGACGGACATGATGGGCCTCACGTTTTCGGAACACCCGGCAATCAAGTCTACATTCGGGGGCAGAACGATGGCGTATACAAGGTGCCAGGAGTAGGTGGTCAGTTCCATCACGCATCGTCGCCAGCAGATCACGTCTATACGGATGAGCAGGGGATCACGTATGTGCACAAAAAAGACGCTGGAGGACCAGGAACACATACGCTTAGAGGACCAGGTCTAGCTGCTGTACAGCCCGCCTACTCCACTGTGCAGCCTGCGGGACTTCGCAATCCCCAGTTTCATGTGGAGCGAAGTGGTCGCACTGTGGATGTTGGTTCTGGAGGATTTGTCGTCCAAAGGAGCAGACGTAGTCCTCAGTTCCATGTGGAGCGTCCTGGTCGCACTGTGGATGTGGGTTCCGGAGGATTTTTTGTCCAAAGGGGCAGGCGTAGTCCCCAGTTTCCTGTGGAGCGACCTTGGCAAAGGGTCGACGTCGGCTTTGAAGGAC>line_837ATGGTCTCGCCCAAACGGAGACTAATCGACGATTCCATTGCTCCCTCAGCTGCCGATCTCCAGCGGACATACGACGGACATGATGGGCCTCACGTTTTCGGAACACCCGGCAATCAAGTCTACATTCGGGGGCAGAACGATGGCGTATACAAGGTGCCAGGAGTAGGTGGTCAGTTCCATCACGCATCGTCGCCAGCAGATCACGTCTATACGGATGAGCAGGGGATCACGTATGTGCACAAAAAAGACGCTGGAGGACCAGGAACACATACGCTTAGAGGACCAGGTCTAGCTGCTGTACAGCCCGCCTACTCCACTGTGCAGCCTGCGGGACTTCGCAATCCCCAGTTTCATGTGGAGCGAAGTGGTCGCACTGTGGATGTTGGTTCTGGAGGATTTGTCGTCCAAAGGAGCAGACGTAGTCCTCAGTTCCATGTGGAGCGTCCTGGTCGCACTGTGGATGTGGGTTCCGGAGGATTTTTTGTCCAAAGGGGCAGGCGTAGTCCCCAGTTTCCTGTGGAGCGACCTTGGCAAAGGGTCGACGTCGGCTTTGAAGGAC>line_849ATGGTCTCGCCCAAACGGAGACTAATCGACGATTCCATTGCTCCCTCAGCTGCCGATCTCCAGCGGACATACGACGGACATGATGGGCCTCACGTTTTCGGAACACCCGGCAATCAAGTCTACATTCGGGGGCAGAACGATGGCGTATACAAGGTGCCAGGAGTAGGTGGTCAGTTCCATCACGCATCGTCGCCAGCAGATCACGTCTATACGGATGAGCAGGGGATCACGTATGTGCACAAAAAAGACGCTGGAGGACCAGGAACACATACGCTTAGAGGACCAGGTCTAGCTGCTGTACAGCCCGCCTACTCCACTGTGCAGCCTGCGGGACTTCGCAATCCCCAGTTTCATGTGGAGCGAAGTGGTCGCACTGTGGATGTTGGTTCTGGAGGATTTGTCGTCCAAAGGAGCAGACGTAGTCCTCAGTTCCATGTGGAGCGTCCTGGTCGCACTGTGGATGTGGGTTCCGGAGGATTTTTTGTCCAAAGGGGCAGGCGTAGTCCCCAGTTTCCTGTGGAGCGACCTTGGCAAAGGGTCGACGTCGGCTTTGAAGGAC>line_852ATGGTCTCGCCCAAACGGAGACTAATCGACGATTCCATTGCTCCCTCAGCTGCCGATCTCCAGCGGACATACGACGGACATGATGGGCCTCACGTTTTCGGAACACCCGGCAATCAAGTCTACATTCGGGGGCAGAACGATGGCGTATACAAGGTGCCAGGAGTAGGTGGTCAGTTCCATCACGCATCGTCGCCAGCAGATCACGTCTATACGGATGAGCAGGGGATCACGTATGTGCACAAAAAAGACGCTGGAGGACCAGGAACACATACGCTTAGAGGACCAGGTCTAGCTGCTGTACAGCCCGCCTACTCCACTGTGCAGCCTGCGGGACTTCGCAATCCCCAGTTTCATGTGGAGCGAAGTGGTCGCACTGTGGATGTTGGTTCTGGAGGATTTGTCGTCCAAAGGAGCAGACGTAGTCCTCAGTTCCATGTGGAGCGTCCTGGTCGCACTGTGGATGTGGGTTCCGGAGGATTTTTTGTCCAAAGGGGCAGGCGTAGTCCCCAGTTTCCTGTGGAGCGACCTTGGCAAAGGGTCGACGTCGGCTTTGAAGGAC>line_879ATGGTCTCGCCCAAACGGAGACTAATCGACGATTCCATTGCTCCCTCAGCTGCCGATCTCCAGCGGACATACGACGGACATGATGGGCCTCACGTTTTCGGAACACCCGGCAATCAAGTCTACATTCGGGGGCAGAACGATGGCGTATACAAGGTGCCAGGAGTAGGTGGTCAGTTCCATCACGCATCGTCGCCAGCAGATCACGTCTATACGGATGAGCAGGGGATCACGTATGTGCACAAAAAAGACGCTGGAGGACCAGGAACACATACGCTTAGAGGACCAGGTCTAGCTGCTGTACAGCCCGCCTACTCCACTGTGCAGCCTGCGGGACTTCGCAATCCCCAGTTTCATGTGGAGCGAAGTGGTCGCACTGTGGATGTTGGTTCTGGAGGATTTGTCGTCCAAAGGAGCAGACGTAGTCCTCAGTTCCATGTGGAGCGTCCTGGTCGCACTGTGGATGTGGGTTCCGGAGGATTTTTTGTCCAAAGGGGCAGGCGTAGTCCCCAGTTTCCTGTGGAGCGACCTTGGCAAAGGGTCGACGTCGGCTTTGAAGGAC>line_890ATGGTCTCGCCCAAACGGAGACTAATCGACGATTCCATTGCTCCCTCAGCTGCCGATCTCCAGCGGACATACGACGGACATGATGGGCCTCACGTTTTCGGAACACCCGGCAATCAAGTCTACATTCGGGGGCAGAACGATGGCGTATACAAGGTGCCAGGAGTAGGTGGTCAGTTCCATCACGCATCGTCGCCAGCAGATCACGTCTATACGGATGAGCAGGGGATCACGTATGTGCACAAAAAAGACGCTGGAGGACCAGGAACACATACGCTTAGAGGACCAGGTCTAGCTGCTGTACAGCCCGCCTACTCCACTGTGCAGCCTGCGGGACTTCGCAATCCCCAGTTTCATGTGGAGCGAAGTGGTCGCACTGTGGATGTTGGTTCTGGAGGATTTGTCGTCCAAAGGAGCAGACGTAGTCCTCAGTTCCATGTGGAGCGTCCTGGTCGCACTGTGGATGTGGGTTCCGGAGGATTTTTTGTCCAAAGGGGCAGGCGTAGTCCCCAGTTTCCTGTGGAGCGACCTTGGCAAAGGGTCGACGTCGGCTTTGAAGGAC>line_892ATGGTCTCGCCCAAACGGAGACTAATCGACGATTCCATTGCTCCCTCAGCTGCCGATCTCCAGCGGACATACGACGGACATGATGGGCCTCACGTTTTCGGAACACCCGGCAATCAAGTCTACATTCGGGGGCAGAACGATGGCGTATACAAGGTGCCAGGAGTAGGTGGTCAGTTCCATCACGCATCGTCGCCAGCAGATCACGTCTATACGGATGAGCAGGGGATCACGTATGTGCACAAAAAAGACGCTGGAGGACCAGGAACACATACGCTTAGAGGACCAGGTCTAGCTGCTGTACAGCCCGCCTACTCCACTGTGCAGCCTGCGGGACTTCGCAATCCCCAGTTTCATGTGGAGCGAAGTGGTCGCACTGTGGATGTTGGTTCTGGAGGATTTGTCGTCCAAAGGAGCAGACGTAGTCCTCAGTTCCATGTGGAGCGTCCTGGTCGCACTGTGGATGTGGGTTCCGGAGGATTTTTTGTCCAAAGGGGCAGGCGTAGTCCCCAGTTTCCTGTGGAGCGACCTTGGCAAAGGGTCGACGTCGGCTTTGAAGGAC>line_897ATGGTCTCGCCCAAACGGAGACTAATCGACGATTCCATTGCTCCCTCAGCTGCCGATCTCCAGCGGACATACGACGGACATGATGGGCCTCACGTTTTCGGAACACCCGGCAATCAAGTCTACATTCGGGGGCAGAACGATGGCGTATACAAGGTGCCAGGAGTAGGTGGTCAGTTCCATCACGCATCGTCGCCAGCAGATCACGTCTATACGGATGAGCAGGGGATCACGTATGTGCACAAAAAAGACGCTGGAGGACCAGGAACACATACGCTTAGAGGACCAGGTCTAGCTGCTGTACAGCCCGCCTACTCCACTGTGCAGCCTGCGGGACTTCGCAATCCCCAGTTTCATGTGGAGCGAAGTGGTCGCACTGTGGATGTTGGTTCTGGAGGATTTGTCGTCCAAAGGAGCAGACGTAGTCCTCAGTTCCATGTGGAGCGTCCTGGTCGCACTGTGGATGTGGGTTCCGGAGGATTTTTTGTCCAAAGGGGCAGGCGTAGTCCCCAGTTTCCTGTGGAGCGACCTTGGCAAAGGGTCGACGTCGGCTTTGAAGGAC>line_911ATGGTCTCGCCCAAACGGAGACTAATCGACGATTCCATTGCTCCCTCAGCTGCCGATCTCCAGCGGACATACGACGGACATGATGGGCCTCACGTTTTCGGAACACCCGGCAATCAAGTCTACATTCGGGGGCAGAACGATGGCGTATACAAGGTGCCAGGAGTAGGTGGTCAGTTCCATCACGCATCGTCGCCAGCAGATCACGTCTATACGGATGAGCAGGGGATCACGTATGTGCACAAAAAAGACGCTGGAGGACCAGGAACACATACGCTTAGAGGACCAGGTCTAGCTGCTGTACAGCCCGCCTACTCCACTGTGCAGCCTGCGGGACTTCGCAATCCCCAGTTTCATGTGGAGCGAAGTGGTCGCACTGTGGATGTTGGTTCTGGAGGATTTGTCGTCCAAAGGAGCAGACGTAGTCCTCAGTTCCATGTGGAGCGTCCTGGTCGCACTGTGGATGTGGGTTCCGGAGGATTTTTTGTCCAAAGGGGCAGGCGTAGTCCCCAGTTTCCTGTGGAGCGACCTTGGCAAAGGGTCGACGTCGGCTTTGAAGGAC>line_233ATGGTCTCGCCCAAACGGAGACTAATCGACGATTCCATTGCTCCCTCAGCTGCCGATCTCCAGCGGACATACGACGGACATGATGGGCCTCACGTTTTCGGAACACCCGGCAATCAAGTCTACATTCGGGGGCAGAACGATGGCGTATACAAGGTGCCAGGAGTAGGTGGTCAGTTCCATCACGCATCGTCGCCAGCAGATCACGTCTATACGGATGAGCAGGGGATCACGTATGTGCACAAAAAAGACGCTGGAGGACCAGGAACACATACGCTTAGAGGACCAGGTCTAGCTGCTGTACAGCCCGCCTACTCCACTGTGCAGCCTGCGGGACTTCGCAATCCCCAGTTTCATGTGGAGCGAAGTGGTCGCACTGTGGATGTTGGTTCTGGAGGATTTATCGTCCAAAGGAGCAGACGTAGTCCTCAGTTCCATGTGGAGCGTCCTGGTCGCACTGTGGATGTGGGTTCCGGAGGATTTTTTGTCCAAAGGGGCAGGCGTAGTCCCCAGTTTCCTGTGGAGCGACCTTGGCAAAGGGTCGACGTCGGCTTTGAAGGAC>line_443ATGGTCTCGCCCAAACGGAGACTAATCGACGATTCCATTGCTCCCTCAGCTGCCGATCTCCAGCGGACATACGACGGACATGATGGGCCTCACGTTTTCGGAACACCCGGCAATCAAGTCTACATTCGGGGGCAGAACGATGGCGTATACAAGGTGCCAGGAGTAGGTGGTCAGTTCCATCACGCATCGTCGCCAGCAGATCACGTCTATACGGATGAGCAGGGGATCACGTATGTGCACAAAAAAGACGCTGGAGGACCAGGAACACATACGCTTAGAGGACCAGGTCTAGCTGCTGTACAGCCCGCCTACTCCACTGTGCAGCCTGCGGGACTTCGCAATCCCCAGTTTCATGTGGAGCGAAGTGGTCGCACTGTGGATGTTGGTTCTGGAGGATTTATCGTCCAAAGGAGCAGACGTAGTCCTCAGTTCCATGTGGAGCGTCCTGGTCGCACTGTGGATGTGGGTTCCGGAGGATTTTTTGTCCAAAGGGGCAGGCGTAGTCCCCAGTTTCCTGTGGAGCGACCTTGGCAAAGGGTCGACGTCGGCTTTGAAGGAC>line_492ATGGTCTCGCCCAAACGGAGACTAATCGACGATTCCATTGCTCCCTCAGCTGCCGATCTCCAGCGGACATACGACGGACATGATGGGCCTCACGTTTTCGGAACACCCGGCAATCAAGTCTACATTCGGGGGCAGAACGATGGCGTATACAAGGTGCCAGGAGTAGGTGGTCAGTTCCATCACGCATCGTCGCCAGCAGATCACGTCTATACGGATGAGCAGGGGATCACGTATGTGCACAAAAAAGACGCTGGAGGACCAGGAACACATACGCTTAGAGGACCAGGTCTAGCTGCTGTACAGCCCGCCTACTCCACTGTGCAGCCTGCGGGACTTCGCAATCCCCAGTTTCATGTGGAGCGAAGTGGTCGCACTGTGGATGTTGGTTCTGGAGGATTTATCGTCCAAAGGAGCAGACGTAGTCCTCAGTTCCATGTGGAGCGTCCTGGTCGCACTGTGGATGTGGGTTCCGGAGGATTTTTTGTCCAAAGGGGCAGGCGTAGTCCCCAGTTTCCTGTGGAGCGACCTTGGCAAAGGGTCGACGTCGGCTTTGAAGGAC>line_409ATGGTCTCGCCCAAACGGAGACTAATCGACGATTCCATTGCTCCCTCAGCTGCCGATCTCCAGCAGACATACGACGGACATGATGGGCCTCACGTTTTCGGAACACCCGGCAATCAAGTCTACATTCGGGGGCAGAACGATGGCGTATACAAGGTGCCAGGAGTAGGTGGTCAGTTCCATCACGCATCGTCGCCAGCAGATCACGTCTATACGGATGAGCAGGGGATCACGTATGTGCACAAAAAAGACGCTGGAGGACCAGGAACACATACGCTTAGAGGACCAGGTCTAGCTGCTGTACAGCCCGCCTACTCCACTGTGCAGCCTGCGGGACTTCGCAATCCCCAGTTTCATGTGGAGCGAAGTGGTCGCACTGTGGATGTTGGTTCTGGAGGATTTGTCGTCCAAAGGAGCAGACGTAGTCCTCAGTTCCATGTGGAGCGTCCTGGTCGCACTGTGGATGTGGGTTCCGGAGGATTTTTTGTCCAAAGGGGCAGGCGTAGTCCCCAGTTTCCTGTGGAGCGACCTTGGCAAAGGGTCGACGTCGGCTTTGAAGGAC>line_555ATGGTCTCGCCCAAACGGAGACTAATCGACGATTCCATTGCTCCCTCAGCTGCCGATCTCCAGCAGACATACGACGGACATGATGGGCCTCACGTTTTCGGAACACCCGGCAATCAAGTCTACATTCGGGGGCAGAACGATGGCGTATACAAGGTGCCAGGAGTAGGTGGTCAGTTCCATCACGCATCGTCGCCAGCAGATCACGTCTATACGGATGAGCAGGGGATCACGTATGTGCACAAAAAAGACGCTGGAGGACCAGGAACACATACGCTTAGAGGACCAGGTCTAGCTGCTGTACAGCCCGCCTACTCCACTGTGCAGCCTGCGGGACTTCGCAATCCCCAGTTTCATGTGGAGCGAAGTGGTCGCACTGTGGATGTTGGTTCTGGAGGATTTGTCGTCCAAAGGAGCAGACGTAGTCCTCAGTTCCATGTGGAGCGTCCTGGTCGCACTGTGGATGTGGGTTCCGGAGGATTTTTTGTCCAAAGGGGCAGGCGTAGTCCCCAGTTTCCTGTGGAGCGACCTTGGCAAAGGGTCGACGTCGGCTTTGAAGGAC>line_730ATGGTCTCGCCCAAACGGAGACTAATCGACGATTCCATTGCTCCCTCAGCTGCCGATCTCCAGCAGACATACGACGGACATGATGGGCCTCACGTTTTCGGAACACCCGGCAATCAAGTCTACATTCGGGGGCAGAACGATGGCGTATACAAGGTGCCAGGAGTAGGTGGTCAGTTCCATCACGCATCGTCGCCAGCAGATCACGTCTATACGGATGAGCAGGGGATCACGTATGTGCACAAAAAAGACGCTGGAGGACCAGGAACACATACGCTTAGAGGACCAGGTCTAGCTGCTGTACAGCCCGCCTACTCCACTGTGCAGCCTGCGGGACTTCGCAATCCCCAGTTTCATGTGGAGCGAAGTGGTCGCACTGTGGATGTTGGTTCTGGAGGATTTGTCGTCCAAAGGAGCAGACGTAGTCCTCAGTTCCATGTGGAGCGTCCTGGTCGCACTGTGGATGTGGGTTCCGGAGGATTTTTTGTCCAAAGGGGCAGGCGTAGTCCCCAGTTTCCTGTGGAGCGACCTTGGCAAAGGGTCGACGTCGGCTTTGAAGGAC>line_861ATGGTCTCGCCCAAACGGAGACTAATCGACGATTCCATTGCTCCCTCAGCTGCCGATCTCCAGCAGACATACGACGGACATGATGGGCCTCACGTTTTCGGAACACCCGGCAATCAAGTCTACATTCGGGGGCAGAACGATGGCGTATACAAGGTGCCAGGAGTAGGTGGTCAGTTCCATCACGCATCGTCGCCAGCAGATCACGTCTATACGGATGAGCAGGGGATCACGTATGTGCACAAAAAAGACGCTGGAGGACCAGGAACACATACGCTTAGAGGACCAGGTCTAGCTGCTGTACAGCCCGCCTACTCCACTGTGCAGCCTGCGGGACTTCGCAATCCCCAGTTTCATGTGGAGCGAAGTGGTCGCACTGTGGATGTTGGTTCTGGAGGATTTGTCGTCCAAAGGAGCAGACGTAGTCCTCAGTTCCATGTGGAGCGTCCTGGTCGCACTGTGGATGTGGGTTCCGGAGGATTTTTTGTCCAAAGGGGCAGGCGTAGTCCCCAGTTTCCTGTGGAGCGACCTTGGCAAAGGGTCGACGTCGGCTTTGAAGGAC>line_517ATGGTCTCGCCCAAACGGAGACTAATCGACGATTCCATTGCTCCCTCAGCTGCCGATCTCCAGCGGACATACGACGGATATGATGGGCCTCACGTTTTCGGAACACCCGGCAATCAAGTCTACATTCGGGGGCAGAACGATGGCGTATACAAGGTGCCAGGAGTAGGTGGTCAGTTCCATCACGCATCGTCGCCAGCAGATCACGTCTATACGGATGAGCAGGGGATCACGTATGTGCACAAAAAAGACGCTGGAGGACCAGGAACACATACGCTTAGAGGACCAGGTCTAGCTGCTGTACAGCCCGCCTACTCCACTGTGCAGCCTGCGGGACTTCGCAATCCCCAGTTTCATGTGGAGCGAAGTGGTCGCACTGTGGATGTTGGTTCTGGAGGATTTGTCGTCCAAAGGAGCAGACGTAGTCCTCAGTTCCATGTGGAGCGTCCTGGTCGCACTGTGGATGTGGGTTCCGGAGGATTTTTTGTCCAAAGGGGCAGGCGTAGTCCCCAGTTTCCTGTGGAGCGACCTTGGCAAAGGGTCGACGTCGGCTTTGAAGGAC>line_757ATGGTCTCGCCCAAACGGAGACTAATCGACGATTCCATTGCTCCCTCAGCTGCCGATCTCCAGCGGACATACGACGGATATGATGGGCCTCACGTTTTCGGAACACCCGGCAATCAAGTCTACATTCGGGGGCAGAACGATGGCGTATACAAGGTGCCAGGAGTAGGTGGTCAGTTCCATCACGCATCGTCGCCAGCAGATCACGTCTATACGGATGAGCAGGGGATCACGTATGTGCACAAAAAAGACGCTGGAGGACCAGGAACACATACGCTTAGAGGACCAGGTCTAGCTGCTGTACAGCCCGCCTACTCCACTGTGCAGCCTGCGGGACTTCGCAATCCCCAGTTTCATGTGGAGCGAAGTGGTCGCACTGTGGATGTTGGTTCTGGAGGATTTGTCGTCCAAAGGAGCAGACGTAGTCCTCAGTTCCATGTGGAGCGTCCTGGTCGCACTGTGGATGTGGGTTCCGGAGGATTTTTTGTCCAAAGGGGCAGGCGTAGTCCCCAGTTTCCTGTGGAGCGACCTTGGCAAAGGGTCGACGTCGGCTTTGAAGGAC>line_855ATGGTCTCGCCCAAACGGAGACTAATCGACGATTCCATTGCTCCCTCAGCTGCCGATCTCCAGCGGACATACGACGGATATGATGGGCCTCACGTTTTCGGAACACCCGGCAATCAAGTCTACATTCGGGGGCAGAACGATGGCGTATACAAGGTGCCAGGAGTAGGTGGTCAGTTCCATCACGCATCGTCGCCAGCAGATCACGTCTATACGGATGAGCAGGGGATCACGTATGTGCACAAAAAAGACGCTGGAGGACCAGGAACACATACGCTTAGAGGACCAGGTCTAGCTGCTGTACAGCCCGCCTACTCCACTGTGCAGCCTGCGGGACTTCGCAATCCCCAGTTTCATGTGGAGCGAAGTGGTCGCACTGTGGATGTTGGTTCTGGAGGATTTGTCGTCCAAAGGAGCAGACGTAGTCCTCAGTTCCATGTGGAGCGTCCTGGTCGCACTGTGGATGTGGGTTCCGGAGGATTTTTTGTCCAAAGGGGCAGGCGTAGTCCCCAGTTTCCTGTGGAGCGACCTTGGCAAAGGGTCGACGTCGGCTTTGAAGGAC>line_129ATGGTCTCGCCCAAACGGAGACTAATCGACGATTCCATTGCTCCCTCAGCTGCCGATCTCCAGCGGACATACGACGGACATGATGGGCCTCACGTTTTCGGAACACCCGGCAATCAAGTCTACATTCGGGGGCAGAACGATGGCGTATACAAGGTGCCAGGAGTAGGTGGTCAGTTCCATCACGCATCGTCGCCAGCAGATCACGTCTATACGGATGAGCAGGGGATCACGTATGTGCACAAAAAAGACGCTGGAGGACCAGGAACACATACGCTTAGAGGACCAGGTCTAGCTGCTGTACAGCCCGCCTACTCCACTGTGCAGCCTGCGGGACTTCGCAATCCCCAGTTTCATGTGGAGCGAAGTGGTCGCACTGTGGATGTTGGTTCTGGAGGATTTGTCGTCCAAAGGAGCAGACGTAGTCCTCAGTTCCATGTGGAGCGTCCTGGTCGCACTGTGGTTGTGGGTTCCGGAGGATTTTTTGTCCAAAGGGGCAGGCGTAGTCCCCAGTTTCCTGTGGAGCGACCTTGGCAAAGGGTCGACGTCGGCTTTGAAGGAC>line_228ATGGTCTCGCCCAAACGGAGACTAATCGACGATTCCATTGCTCCCTCAGCTGCCGATCTCCAGCGGACATACGACGGACATGATGGGCCTCACGTTTTCGGAACACCCGGCAATCAAGTCTACATTCGGGGGCAGAACGATGGCGTATACAAGGTGCCAGGAGTAGGTGGTCAGTTCCATCACGCATCGTCGCCAGCAGATCACGTCTATACGGATGAGCAGGGGATCACGTATGTGCACAAAAAAGACGCTGGAGGACCAGGAACACATACGCTTAGAGGACCAGGTCTAGCTGCTGTACAGCCCGCCTACTCCACTGTGCAGCCTGCGGGACTTCGCAATCCCCAGTTTCATGTGGAGCGAAGTGGTCGCACTGTGGATGTTGGTTCTGGAGGATTTGTCGTCCAAAGGAGCAGACGTAGTCCTCAGTTCCATGTGGAGCGTCCTGGTCGCACTGTGGTTGTGGGTTCCGGAGGATTTTTTGTCCAAAGGGGCAGGCGTAGTCCCCAGTTTCCTGTGGAGCGACCTTGGCAAAGGGTCGACGTCGGCTTTGAAGGAC>line_367ATGGTCTCGCCCAAACGGAGACTAATCGACGATTCCATTGCTCCCTCAGCTGCCGATCTCCAGCGGACATACGACGGACATGATGGGCCTCACGTTTTCGGAACACCCGGCAATCAAGTCTACATTCGGGGGCAGAACGATGGCGTATACAAGGTGCCAGGAGTAGGTGGTCAGTTCCATCACGCATCGTCGCCAGCAGATCACGTCTATACGGATGAGCAGGGGATCACGTATGTGCACAAAAAAGACGCTGGAGGACCAGGAACACATACGCTTAGAGGACCAGGTCTAGCTGCTGTACAGCCCGCCTACTCCACTGTGCAGCCTGCGGGACTTCGCAATCCCCAGTTTCATGTGGAGCGAAGTGGTCGCACTGTGGATGTTGGTTCTGGAGGATTTGTCGTCCAAAGGAGCAGACGTAGTCCTCAGTTCCATGTGGAGCGTCCTGGTCGCACTGTGGTTGTGGGTTCCGGAGGATTTTTTGTCCAAAGGGGCAGGCGTAGTCCCCAGTTTCCTGTGGAGCGACCTTGGCAAAGGGTCGACGTCGGCTTTGAAGGAC>line_799ATGGTCTCGCCCAAACGGAGACTAATCGACGATTCCATTGCTCCCTCAGCTGCCGATCTCCAGCGGACATACGACGGACATGATGGGCCTCACGTTTTCGGAACACCCGGCAATCAAGTCTACATTCGGGGGCAGAACGATGGCGTATACAAGGTGCCAGGAGTAGGTGGTCAGTTCCATCACGCATCGTCGCCAGCAGATCACGTCTATACGGATGAGCAGGGGATCACGTATGTGCACAAAAAAGACGCTGGAGGACCAGGAACACATACGCTTAGAGGACCAGGTCTAGCTGCTGTACAGCCCGCCTACTCCACTGTGCAGCCTGCGGGACTTCGCAATCCCCAGTTTCATGTGGAGCGAAGTGGTCGCACTGTGGATGTTGGTTCTGGAGGATTTGTCGTCCAAAGGAGCAGACGTAGTCCTCAGTTCCATGTGGAGCGTCCTGGTCGCACTGTGGTTGTGGGTTCCGGAGGATTTTTTGTCCAAAGGGGCAGGCGTAGTCCCCAGTTTCCTGTGGAGCGACCTTGGCAAAGGGTCGACGTCGGCTTTGAAGGAC>line_42ATGGTCTCGCCCAAACGGAGACTAATCGACGATTCCATTGCTCCCTCAGCTGCCGATCTCCAGCGGACATACGACGGACATGATGGGCCTCACGTTTTCGGAACACCCGGCAATCAAGTCTACATTCGGGGGCAGAACGATGGCGTATACAAGGTGCCAGGAGTAGGTGGTCAGTTCTATCACGCATCGTCGCCAGCAGAACACGTCTATACGGATGAGCAGGGGATCACGTATGTGCACAAAAAAGACGCTGGAGGACCAGGAACACATACGCTTAGAGGACCAGGTCTAGCTGCTGTACAGCCCGCCTACTCCACTGTGCAGCCTGCGGGACTTCGCAATCCCCAGTTTCATGTGGAGCGAAGTGGTCGCACTGTGGATGTTGGTTCTGGAGGATTTGTCGTCCAAAGGAGCAGACGTAGTCCTCAGTTCCATGTGGAGCGTCCTGGTCGCACTGTGGATGTGGGTTCCGGAGGATTTTTTGTCCAAAGGGGCAGGCGTAGTCCCCAGTTTCCTGTGGAGCGACCTTGGCAAAGGGTCGACGTCGGCTTTGAAGGAC>line_59ATGGTCTCGCCCAAACGGAGACTAATCGACGATTCCATTGCTCCCTCAGCTGCCGATCTCCAGCGGACATACGACGGACATGATGGGCCTCACGTTTTCGGAACACCCGGCAATCAAGTCTACATTCGGGGGCAGAACGATGGCGTATACAAGGTGCCAGGAGTAGGTGGTCAGTTCTATCACGCATCGTCGCCAGCAGAACACGTCTATACGGATGAGCAGGGGATCACGTATGTGCACAAAAAAGACGCTGGAGGACCAGGAACACATACGCTTAGAGGACCAGGTCTAGCTGCTGTACAGCCCGCCTACTCCACTGTGCAGCCTGCGGGACTTCGCAATCCCCAGTTTCATGTGGAGCGAAGTGGTCGCACTGTGGATGTTGGTTCTGGAGGATTTGTCGTCCAAAGGAGCAGACGTAGTCCTCAGTTCCATGTGGAGCGTCCTGGTCGCACTGTGGATGTGGGTTCCGGAGGATTTTTTGTCCAAAGGGGCAGGCGTAGTCCCCAGTTTCCTGTGGAGCGACCTTGGCAAAGGGTCGACGTCGGCTTTGAAGGAC>line_69ATGGTCTCGCCCAAACGGAGACTAATCGACGATTCCATTGCTCCCTCAGCTGCCGATCTCCAGCGGACATACGACGGACATGATGGGCCTCACGTTTTCGGAACACCCGGCAATCAAGTCTACATTCGGGGGCAGAACGATGGCGTATACAAGGTGCCAGGAGTAGGTGGTCAGTTCTATCACGCATCGTCGCCAGCAGAACACGTCTATACGGATGAGCAGGGGATCACGTATGTGCACAAAAAAGACGCTGGAGGACCAGGAACACATACGCTTAGAGGACCAGGTCTAGCTGCTGTACAGCCCGCCTACTCCACTGTGCAGCCTGCGGGACTTCGCAATCCCCAGTTTCATGTGGAGCGAAGTGGTCGCACTGTGGATGTTGGTTCTGGAGGATTTGTCGTCCAAAGGAGCAGACGTAGTCCTCAGTTCCATGTGGAGCGTCCTGGTCGCACTGTGGATGTGGGTTCCGGAGGATTTTTTGTCCAAAGGGGCAGGCGTAGTCCCCAGTTTCCTGTGGAGCGACCTTGGCAAAGGGTCGACGTCGGCTTTGAAGGAC>line_93ATGGTCTCGCCCAAACGGAGACTAATCGACGATTCCATTGCTCCCTCAGCTGCCGATCTCCAGCGGACATACGACGGACATGATGGGCCTCACGTTTTCGGAACACCCGGCAATCAAGTCTACATTCGGGGGCAGAACGATGGCGTATACAAGGTGCCAGGAGTAGGTGGTCAGTTCTATCACGCATCGTCGCCAGCAGAACACGTCTATACGGATGAGCAGGGGATCACGTATGTGCACAAAAAAGACGCTGGAGGACCAGGAACACATACGCTTAGAGGACCAGGTCTAGCTGCTGTACAGCCCGCCTACTCCACTGTGCAGCCTGCGGGACTTCGCAATCCCCAGTTTCATGTGGAGCGAAGTGGTCGCACTGTGGATGTTGGTTCTGGAGGATTTGTCGTCCAAAGGAGCAGACGTAGTCCTCAGTTCCATGTGGAGCGTCCTGGTCGCACTGTGGATGTGGGTTCCGGAGGATTTTTTGTCCAAAGGGGCAGGCGTAGTCCCCAGTTTCCTGTGGAGCGACCTTGGCAAAGGGTCGACGTCGGCTTTGAAGGAC>line_235ATGGTCTCGCCCAAACGGAGACTAATCGACGATTCCATTGCTCCCTCAGCTGCCGATCTCCAGCGGACATACGACGGACATGATGGGCCTCACGTTTTCGGAACACCCGGCAATCAAGTCTACATTCGGGGGCAGAACGATGGCGTATACAAGGTGCCAGGAGTAGGTGGTCAGTTCTATCACGCATCGTCGCCAGCAGAACACGTCTATACGGATGAGCAGGGGATCACGTATGTGCACAAAAAAGACGCTGGAGGACCAGGAACACATACGCTTAGAGGACCAGGTCTAGCTGCTGTACAGCCCGCCTACTCCACTGTGCAGCCTGCGGGACTTCGCAATCCCCAGTTTCATGTGGAGCGAAGTGGTCGCACTGTGGATGTTGGTTCTGGAGGATTTGTCGTCCAAAGGAGCAGACGTAGTCCTCAGTTCCATGTGGAGCGTCCTGGTCGCACTGTGGATGTGGGTTCCGGAGGATTTTTTGTCCAAAGGGGCAGGCGTAGTCCCCAGTTTCCTGTGGAGCGACCTTGGCAAAGGGTCGACGTCGGCTTTGAAGGAC>line_309ATGGTCTCGCCCAAACGGAGACTAATCGACGATTCCATTGCTCCCTCAGCTGCCGATCTCCAGCGGACATACGACGGACATGATGGGCCTCACGTTTTCGGAACACCCGGCAATCAAGTCTACATTCGGGGGCAGAACGATGGCGTATACAAGGTGCCAGGAGTAGGTGGTCAGTTCTATCACGCATCGTCGCCAGCAGAACACGTCTATACGGATGAGCAGGGGATCACGTATGTGCACAAAAAAGACGCTGGAGGACCAGGAACACATACGCTTAGAGGACCAGGTCTAGCTGCTGTACAGCCCGCCTACTCCACTGTGCAGCCTGCGGGACTTCGCAATCCCCAGTTTCATGTGGAGCGAAGTGGTCGCACTGTGGATGTTGGTTCTGGAGGATTTGTCGTCCAAAGGAGCAGACGTAGTCCTCAGTTCCATGTGGAGCGTCCTGGTCGCACTGTGGATGTGGGTTCCGGAGGATTTTTTGTCCAAAGGGGCAGGCGTAGTCCCCAGTTTCCTGTGGAGCGACCTTGGCAAAGGGTCGACGTCGGCTTTGAAGGAC>line_332ATGGTCTCGCCCAAACGGAGACTAATCGACGATTCCATTGCTCCCTCAGCTGCCGATCTCCAGCGGACATACGACGGACATGATGGGCCTCACGTTTTCGGAACACCCGGCAATCAAGTCTACATTCGGGGGCAGAACGATGGCGTATACAAGGTGCCAGGAGTAGGTGGTCAGTTCTATCACGCATCGTCGCCAGCAGAACACGTCTATACGGATGAGCAGGGGATCACGTATGTGCACAAAAAAGACGCTGGAGGACCAGGAACACATACGCTTAGAGGACCAGGTCTAGCTGCTGTACAGCCCGCCTACTCCACTGTGCAGCCTGCGGGACTTCGCAATCCCCAGTTTCATGTGGAGCGAAGTGGTCGCACTGTGGATGTTGGTTCTGGAGGATTTGTCGTCCAAAGGAGCAGACGTAGTCCTCAGTTCCATGTGGAGCGTCCTGGTCGCACTGTGGATGTGGGTTCCGGAGGATTTTTTGTCCAAAGGGGCAGGCGTAGTCCCCAGTTTCCTGTGGAGCGACCTTGGCAAAGGGTCGACGTCGGCTTTGAAGGAC>line_360ATGGTCTCGCCCAAACGGAGACTAATCGACGATTCCATTGCTCCCTCAGCTGCCGATCTCCAGCGGACATACGACGGACATGATGGGCCTCACGTTTTCGGAACACCCGGCAATCAAGTCTACATTCGGGGGCAGAACGATGGCGTATACAAGGTGCCAGGAGTAGGTGGTCAGTTCTATCACGCATCGTCGCCAGCAGAACACGTCTATACGGATGAGCAGGGGATCACGTATGTGCACAAAAAAGACGCTGGAGGACCAGGAACACATACGCTTAGAGGACCAGGTCTAGCTGCTGTACAGCCCGCCTACTCCACTGTGCAGCCTGCGGGACTTCGCAATCCCCAGTTTCATGTGGAGCGAAGTGGTCGCACTGTGGATGTTGGTTCTGGAGGATTTGTCGTCCAAAGGAGCAGACGTAGTCCTCAGTTCCATGTGGAGCGTCCTGGTCGCACTGTGGATGTGGGTTCCGGAGGATTTTTTGTCCAAAGGGGCAGGCGTAGTCCCCAGTTTCCTGTGGAGCGACCTTGGCAAAGGGTCGACGTCGGCTTTGAAGGAC>line_386ATGGTCTCGCCCAAACGGAGACTAATCGACGATTCCATTGCTCCCTCAGCTGCCGATCTCCAGCGGACATACGACGGACATGATGGGCCTCACGTTTTCGGAACACCCGGCAATCAAGTCTACATTCGGGGGCAGAACGATGGCGTATACAAGGTGCCAGGAGTAGGTGGTCAGTTCTATCACGCATCGTCGCCAGCAGAACACGTCTATACGGATGAGCAGGGGATCACGTATGTGCACAAAAAAGACGCTGGAGGACCAGGAACACATACGCTTAGAGGACCAGGTCTAGCTGCTGTACAGCCCGCCTACTCCACTGTGCAGCCTGCGGGACTTCGCAATCCCCAGTTTCATGTGGAGCGAAGTGGTCGCACTGTGGATGTTGGTTCTGGAGGATTTGTCGTCCAAAGGAGCAGACGTAGTCCTCAGTTCCATGTGGAGCGTCCTGGTCGCACTGTGGATGTGGGTTCCGGAGGATTTTTTGTCCAAAGGGGCAGGCGTAGTCCCCAGTTTCCTGTGGAGCGACCTTGGCAAAGGGTCGACGTCGGCTTTGAAGGAC>line_390ATGGTCTCGCCCAAACGGAGACTAATCGACGATTCCATTGCTCCCTCAGCTGCCGATCTCCAGCGGACATACGACGGACATGATGGGCCTCACGTTTTCGGAACACCCGGCAATCAAGTCTACATTCGGGGGCAGAACGATGGCGTATACAAGGTGCCAGGAGTAGGTGGTCAGTTCTATCACGCATCGTCGCCAGCAGAACACGTCTATACGGATGAGCAGGGGATCACGTATGTGCACAAAAAAGACGCTGGAGGACCAGGAACACATACGCTTAGAGGACCAGGTCTAGCTGCTGTACAGCCCGCCTACTCCACTGTGCAGCCTGCGGGACTTCGCAATCCCCAGTTTCATGTGGAGCGAAGTGGTCGCACTGTGGATGTTGGTTCTGGAGGATTTGTCGTCCAAAGGAGCAGACGTAGTCCTCAGTTCCATGTGGAGCGTCCTGGTCGCACTGTGGATGTGGGTTCCGGAGGATTTTTTGTCCAAAGGGGCAGGCGTAGTCCCCAGTTTCCTGTGGAGCGACCTTGGCAAAGGGTCGACGTCGGCTTTGAAGGAC>line_440ATGGTCTCGCCCAAACGGAGACTAATCGACGATTCCATTGCTCCCTCAGCTGCCGATCTCCAGCGGACATACGACGGACATGATGGGCCTCACGTTTTCGGAACACCCGGCAATCAAGTCTACATTCGGGGGCAGAACGATGGCGTATACAAGGTGCCAGGAGTAGGTGGTCAGTTCTATCACGCATCGTCGCCAGCAGAACACGTCTATACGGATGAGCAGGGGATCACGTATGTGCACAAAAAAGACGCTGGAGGACCAGGAACACATACGCTTAGAGGACCAGGTCTAGCTGCTGTACAGCCCGCCTACTCCACTGTGCAGCCTGCGGGACTTCGCAATCCCCAGTTTCATGTGGAGCGAAGTGGTCGCACTGTGGATGTTGGTTCTGGAGGATTTGTCGTCCAAAGGAGCAGACGTAGTCCTCAGTTCCATGTGGAGCGTCCTGGTCGCACTGTGGATGTGGGTTCCGGAGGATTTTTTGTCCAAAGGGGCAGGCGTAGTCCCCAGTTTCCTGTGGAGCGACCTTGGCAAAGGGTCGACGTCGGCTTTGAAGGAC>line_505ATGGTCTCGCCCAAACGGAGACTAATCGACGATTCCATTGCTCCCTCAGCTGCCGATCTCCAGCGGACATACGACGGACATGATGGGCCTCACGTTTTCGGAACACCCGGCAATCAAGTCTACATTCGGGGGCAGAACGATGGCGTATACAAGGTGCCAGGAGTAGGTGGTCAGTTCTATCACGCATCGTCGCCAGCAGAACACGTCTATACGGATGAGCAGGGGATCACGTATGTGCACAAAAAAGACGCTGGAGGACCAGGAACACATACGCTTAGAGGACCAGGTCTAGCTGCTGTACAGCCCGCCTACTCCACTGTGCAGCCTGCGGGACTTCGCAATCCCCAGTTTCATGTGGAGCGAAGTGGTCGCACTGTGGATGTTGGTTCTGGAGGATTTGTCGTCCAAAGGAGCAGACGTAGTCCTCAGTTCCATGTGGAGCGTCCTGGTCGCACTGTGGATGTGGGTTCCGGAGGATTTTTTGTCCAAAGGGGCAGGCGTAGTCCCCAGTTTCCTGTGGAGCGACCTTGGCAAAGGGTCGACGTCGGCTTTGAAGGAC>line_531ATGGTCTCGCCCAAACGGAGACTAATCGACGATTCCATTGCTCCCTCAGCTGCCGATCTCCAGCGGACATACGACGGACATGATGGGCCTCACGTTTTCGGAACACCCGGCAATCAAGTCTACATTCGGGGGCAGAACGATGGCGTATACAAGGTGCCAGGAGTAGGTGGTCAGTTCTATCACGCATCGTCGCCAGCAGAACACGTCTATACGGATGAGCAGGGGATCACGTATGTGCACAAAAAAGACGCTGGAGGACCAGGAACACATACGCTTAGAGGACCAGGTCTAGCTGCTGTACAGCCCGCCTACTCCACTGTGCAGCCTGCGGGACTTCGCAATCCCCAGTTTCATGTGGAGCGAAGTGGTCGCACTGTGGATGTTGGTTCTGGAGGATTTGTCGTCCAAAGGAGCAGACGTAGTCCTCAGTTCCATGTGGAGCGTCCTGGTCGCACTGTGGATGTGGGTTCCGGAGGATTTTTTGTCCAAAGGGGCAGGCGTAGTCCCCAGTTTCCTGTGGAGCGACCTTGGCAAAGGGTCGACGTCGGCTTTGAAGGAC>line_627ATGGTCTCGCCCAAACGGAGACTAATCGACGATTCCATTGCTCCCTCAGCTGCCGATCTCCAGCGGACATACGACGGACATGATGGGCCTCACGTTTTCGGAACACCCGGCAATCAAGTCTACATTCGGGGGCAGAACGATGGCGTATACAAGGTGCCAGGAGTAGGTGGTCAGTTCTATCACGCATCGTCGCCAGCAGAACACGTCTATACGGATGAGCAGGGGATCACGTATGTGCACAAAAAAGACGCTGGAGGACCAGGAACACATACGCTTAGAGGACCAGGTCTAGCTGCTGTACAGCCCGCCTACTCCACTGTGCAGCCTGCGGGACTTCGCAATCCCCAGTTTCATGTGGAGCGAAGTGGTCGCACTGTGGATGTTGGTTCTGGAGGATTTGTCGTCCAAAGGAGCAGACGTAGTCCTCAGTTCCATGTGGAGCGTCCTGGTCGCACTGTGGATGTGGGTTCCGGAGGATTTTTTGTCCAAAGGGGCAGGCGTAGTCCCCAGTTTCCTGTGGAGCGACCTTGGCAAAGGGTCGACGTCGGCTTTGAAGGAC>line_630ATGGTCTCGCCCAAACGGAGACTAATCGACGATTCCATTGCTCCCTCAGCTGCCGATCTCCAGCGGACATACGACGGACATGATGGGCCTCACGTTTTCGGAACACCCGGCAATCAAGTCTACATTCGGGGGCAGAACGATGGCGTATACAAGGTGCCAGGAGTAGGTGGTCAGTTCTATCACGCATCGTCGCCAGCAGAACACGTCTATACGGATGAGCAGGGGATCACGTATGTGCACAAAAAAGACGCTGGAGGACCAGGAACACATACGCTTAGAGGACCAGGTCTAGCTGCTGTACAGCCCGCCTACTCCACTGTGCAGCCTGCGGGACTTCGCAATCCCCAGTTTCATGTGGAGCGAAGTGGTCGCACTGTGGATGTTGGTTCTGGAGGATTTGTCGTCCAAAGGAGCAGACGTAGTCCTCAGTTCCATGTGGAGCGTCCTGGTCGCACTGTGGATGTGGGTTCCGGAGGATTTTTTGTCCAAAGGGGCAGGCGTAGTCCCCAGTTTCCTGTGGAGCGACCTTGGCAAAGGGTCGACGTCGGCTTTGAAGGAC>line_732ATGGTCTCGCCCAAACGGAGACTAATCGACGATTCCATTGCTCCCTCAGCTGCCGATCTCCAGCGGACATACGACGGACATGATGGGCCTCACGTTTTCGGAACACCCGGCAATCAAGTCTACATTCGGGGGCAGAACGATGGCGTATACAAGGTGCCAGGAGTAGGTGGTCAGTTCTATCACGCATCGTCGCCAGCAGAACACGTCTATACGGATGAGCAGGGGATCACGTATGTGCACAAAAAAGACGCTGGAGGACCAGGAACACATACGCTTAGAGGACCAGGTCTAGCTGCTGTACAGCCCGCCTACTCCACTGTGCAGCCTGCGGGACTTCGCAATCCCCAGTTTCATGTGGAGCGAAGTGGTCGCACTGTGGATGTTGGTTCTGGAGGATTTGTCGTCCAAAGGAGCAGACGTAGTCCTCAGTTCCATGTGGAGCGTCCTGGTCGCACTGTGGATGTGGGTTCCGGAGGATTTTTTGTCCAAAGGGGCAGGCGTAGTCCCCAGTTTCCTGTGGAGCGACCTTGGCAAAGGGTCGACGTCGGCTTTGAAGGAC>line_796ATGGTCTCGCCCAAACGGAGACTAATCGACGATTCCATTGCTCCCTCAGCTGCCGATCTCCAGCGGACATACGACGGACATGATGGGCCTCACGTTTTCGGAACACCCGGCAATCAAGTCTACATTCGGGGGCAGAACGATGGCGTATACAAGGTGCCAGGAGTAGGTGGTCAGTTCTATCACGCATCGTCGCCAGCAGAACACGTCTATACGGATGAGCAGGGGATCACGTATGTGCACAAAAAAGACGCTGGAGGACCAGGAACACATACGCTTAGAGGACCAGGTCTAGCTGCTGTACAGCCCGCCTACTCCACTGTGCAGCCTGCGGGACTTCGCAATCCCCAGTTTCATGTGGAGCGAAGTGGTCGCACTGTGGATGTTGGTTCTGGAGGATTTGTCGTCCAAAGGAGCAGACGTAGTCCTCAGTTCCATGTGGAGCGTCCTGGTCGCACTGTGGATGTGGGTTCCGGAGGATTTTTTGTCCAAAGGGGCAGGCGTAGTCCCCAGTTTCCTGTGGAGCGACCTTGGCAAAGGGTCGACGTCGGCTTTGAAGGAC>line_894ATGGTCTCGCCCAAACGGAGACTAATCGACGATTCCATTGCTCCCTCAGCTGCCGATCTCCAGCGGACATACGACGGACATGATGGGCCTCACGTTTTCGGAACACCCGGCAATCAAGTCTACATTCGGGGGCAGAACGATGGCGTATACAAGGTGCCAGGAGTAGGTGGTCAGTTCTATCACGCATCGTCGCCAGCAGAACACGTCTATACGGATGAGCAGGGGATCACGTATGTGCACAAAAAAGACGCTGGAGGACCAGGAACACATACGCTTAGAGGACCAGGTCTAGCTGCTGTACAGCCCGCCTACTCCACTGTGCAGCCTGCGGGACTTCGCAATCCCCAGTTTCATGTGGAGCGAAGTGGTCGCACTGTGGATGTTGGTTCTGGAGGATTTGTCGTCCAAAGGAGCAGACGTAGTCCTCAGTTCCATGTGGAGCGTCCTGGTCGCACTGTGGATGTGGGTTCCGGAGGATTTTTTGTCCAAAGGGGCAGGCGTAGTCCCCAGTTTCCTGTGGAGCGACCTTGGCAAAGGGTCGACGTCGGCTTTGAAGGAC>line_907ATGGTCTCGCCCAAACGGAGACTAATCGACGATTCCATTGCTCCCTCAGCTGCCGATCTCCAGCGGACATACGACGGACATGATGGGCCTCACGTTTTCGGAACACCCGGCAATCAAGTCTACATTCGGGGGCAGAACGATGGCGTATACAAGGTGCCAGGAGTAGGTGGTCAGTTCTATCACGCATCGTCGCCAGCAGAACACGTCTATACGGATGAGCAGGGGATCACGTATGTGCACAAAAAAGACGCTGGAGGACCAGGAACACATACGCTTAGAGGACCAGGTCTAGCTGCTGTACAGCCCGCCTACTCCACTGTGCAGCCTGCGGGACTTCGCAATCCCCAGTTTCATGTGGAGCGAAGTGGTCGCACTGTGGATGTTGGTTCTGGAGGATTTGTCGTCCAAAGGAGCAGACGTAGTCCTCAGTTCCATGTGGAGCGTCCTGGTCGCACTGTGGATGTGGGTTCCGGAGGATTTTTTGTCCAAAGGGGCAGGCGTAGTCCCCAGTTTCCTGTGGAGCGACCTTGGCAAAGGGTCGACGTCGGCTTTGAAGGAC>line_317ATGGTCTCGCCCAAACGGAGACTAATCGACGATTCCATTGCTCCCTCAGCTGCCGATCTCCAGCGGACATACGACGGACATGATGGGCCTCACGTTTTCGGAACACCCGGCAATCAAGTCTACATTCGGGGGCAGAACGATGGCGTATACAAGGTGCCAGGAGTAGGTGGTCAGTTCCATCACGCATCGTCGCCAGCAGAACACGTCTATACGGATGAGCAGGGGATCACGTATGTGCACAAAAAAGACGCTGGAGGACCAGGAACACATACGCTTAGAGGACCAGGTCTAGCTGCTGTACAGCCCGCCTACTCCACTGTGCAGCCTGCGGGACTTCGCAATCCCCAGTTTCATGTGGAGCGAAGTGGTCGCACTGTGGATGTTGGTTCTGGAGGATTTGTCGTCCAAAGGAGCAGACGTAGTCCTCAGTTCCATGTGGAGCGTCCTGGTCGCACTGTGGATGTGGGTTCCGGAGGATTTTTTGTCCAAAGGGGCAGGCGTAGTCCCCAGTTTCCTGTGGAGCGACCTTGGCAAAGGGTCGACGTCGGCTTTGAAGGAC>line_352ATGGTCTCGCCCAAACGGAGACTAATCGACGATTCCATTGCTCCCTCAGCTGCCGATCTCCAGCGGACATACGACGGACATGATGGGCCTCACGTTTTCGGAACACCCGGCAATCAAGTCTACATTCGGGGGCAGAACGATGGCGTATACAAGGTGCCAGGAGTAGGTGGTCAGTTCCATCACGCATCGTCGCCAGCAGAACACGTCTATACGGATGAGCAGGGGATCACGTATGTGCACAAAAAAGACGCTGGAGGACCAGGAACACATACGCTTAGAGGACCAGGTCTAGCTGCTGTACAGCCCGCCTACTCCACTGTGCAGCCTGCGGGACTTCGCAATCCCCAGTTTCATGTGGAGCGAAGTGGTCGCACTGTGGATGTTGGTTCTGGAGGATTTGTCGTCCAAAGGAGCAGACGTAGTCCTCAGTTCCATGTGGAGCGTCCTGGTCGCACTGTGGATGTGGGTTCCGGAGGATTTTTTGTCCAAAGGGGCAGGCGTAGTCCCCAGTTTCCTGTGGAGCGACCTTGGCAAAGGGTCGACGTCGGCTTTGAAGGAC>line_101ATGGTCTCGCCCAAACGGAGACTAATCGACGATTCCATTGCTCCCTCAGCTGCCGATCTCCAGCGGACATACGACGGATATGATGGGCCTCACGTTTTCGGAACACCCGGCAATCAAGTCTACATTCGGGGGCAGAACGATGGCGTATACAAGGTGCCAGGAGTAGGTGGTCAGTTCCATCACGCATCGTCGCCAGCAGAACACGTCTATACGGATGAGCAGGGGATCACGTATGTGCACAAAAAAGACGCTGGAGGACCAGGAACACATACGCTTAGAGGACCAGGTCTAGCTGCTGTACAGCCCGCCTACTCCACTGTGCAGCCTGCGGGACTTCGCAATCCCCAGTTTCATGTGGAGCGAAGTGGTCGCACTGTGGATGTTGGTTCTGGAGGATTTGTCGTCCAAAGGAGCAGACGTAGTCCTCAGTTCCATGTGGAGCGTCCTGGTCGCACTGTGGATGTGGGTTCCGGAGGATTTTTTGTCCAAAGGGGCAGGCGTAGTCCCCAGTTTCCTGTGGAGCGACCTTGGCAAAGGGTCGACGTCGGCTTTGAAGGAC>line_319ATGGTCTCGCCCAAACGGAGACTAATCGACGATTCCATTGCTCCCTCAGCTGCCGATCTCCAGCGGACATACGACGGACATGATGGGCCTCACGTTTTCGGAACACCCGGCAATCAAGTCTACATTCGGGGGCAGAACGATGGCGTATACAAGGTGCCAGGAGTAGGTGGTCAGTTCCATCACGCATCGTCGCCAGCAGAACACGTCTATACGGATGAGCAGGGGATCACGTATGTGCACAAAAAAGACGCTGGAGGACCAGGAACACATACGCTTAGAGGACCAGGTCTAGCTGCTGTACAGCCCGCCTACTCCACTGTGCAGCCTGCGGGACTTCGCAGTCCCCAGTTTCATGTGGAGCGAAGTGGTCGCACTGTGGATGTTGGTTCTGGAGGATTTGTCGTCCAAAGGAGCAGACGTAGTCCTCAGTTCCATGTGGAGCGTCCTGGTCGCACTGTGGATGTGGGTTCCGGAGGATTTTTTGTCCAAAGGGGCAGGCGTAGTCCCCAGTTTCCTGTGGAGCGACCTTGGCAAAGGGTCGACGTCGGCTTTGAAGGAC>line_377ATGGTCTCGCCCAAACGGAGACTAATCGACGATTCCATTGCTCCCTCAGCTGCCGATCTCCAGCGGACATACGACGGACATGATGGGCCTCACGTTTTCGGAACACCCGGCAATCAAGTCTACATTCGGGGGCAGAACGATGGCGTATACAAGGTGCCAGGAGTAGGTGGTCAGTTCCATCACGCATCGTCGCCAGCAGAACACGTCTATACGGATGAGCAGGGGATCACGTATGTGCACAAAAAAGACGCTGGAGGACCAGGAACACATACGCTTAGAGGACCAGGTCTAGCTGCTGTACAGCCCGCCTACTCCACTGTGCAGCCTGCGGGACTTCGCAGTCCCCAGTTTCATGTGGAGCGAAGTGGTCGCACTGTGGATGTTGGTTCTGGAGGATTTGTCGTCCAAAGGAGCAGACGTAGTCCTCAGTTCCATGTGGAGCGTCCTGGTCGCACTGTGGATGTGGGTTCCGGAGGATTTTTTGTCCAAAGGGGCAGGCGTAGTCCCCAGTTTCCTGTGGAGCGACCTTGGCAAAGGGTCGACGTCGGCTTTGAAGGAC>line_325ATGGTCTCGCCCAAACGGAGACTAATCGACGATTCCATTGCTCCCTCAGCTGCCGATCTCCAGCGGACATACGACGGACATGATGGGCCTCACGTTTTCGGAACACCCGGCAATCAAGTCTACATTCGGGGGCAGAACGATGGCGTATACAAGGTGCCAGGAGTAGGTGGTCAGTTCTATCACGCATCGTCGCCAGCAGAACACGTCTATACGGATGAGCAGGGGATCACGTATGTGCACAAAAAAGACGCTGGAGGACCAGGAACACATACGCTTAGAGGACCAGGTCTAGCTGCTGTACAGCCCGCCTACTCCACTGTGCAGCCTGCGGGACTTCGCAGTCCCCAGTTTCATGTGGAGCGAAGTGGTCGCACTGTGGATGTTGGTTCTGGAGGATTTGTCGTCCAAAGGAGCAGACGTAGTCCTCAGTTCCATGTGGAGCGTCCTGGTCGCACTGTGGATGTGGGTTCCGGAGGATTTTTTGTCCAAAGGGGCAGGCGTAGTCCCCAGTTTCCTGTGGAGCGACCTTGGCAAAGGGTCGACGTCGGCTTTGAAGGAC>line_336ATGGTCTCGCCCAAACGGAGACTAATCGACGATTCCATTGCTCCCTCAGCTGCCGATCTCCAGCGGACATACGACGGACATGATGGGCCTCACGTTTTCGGAACACCCGGCAATCAAGTCTACATTCGGGGGCAGAACGATGGCGTATACAAGGTGCCAGGAGTAGGTGGTCAGTTCTATCACGCATCGTCGCCAGCAGAACACGTCTATACGGATGAGCAGGGGATCACGTATGTGCACAAAAAAGACGCTGGAGGACCAGGAACACATACGCTTAGAGGACCAGGTCTAGCTGCTGTACAGCCCGCCTACTCCACTGTGCAGCCTGCGGGACTTCGCAATCCCCAGTTTCATGTGGAGCGAAGTGGTCGCACTGTGGATGTTGGTTCTGGAGGATTTGTCGTCCAAAGGAGCAGACGTAGTCCTCAGTTCCATGTGGAGCGTCCTGGTCGCACTGTGGATGTGGGTTCCGGAGGATTTTTTGTCCAAAGGGGCAGGCGTAGTCCCCAGTTTCCTGTGGAGCGACCTTGGCAAAGGGTCGACGTCGGCTTTAAGGGAC>line_338ATGGTCTCGCCCAAACGGAGACTAATCGACGATTCCATTGCTCCCTCAGCTGCCGATCTCCAGCGGACATACGACGGACATGATGGGCCTCACGTTTTCGGAACACCCGGCAATCAAGTCTACATTCGGGGGCAGAACGATGGCGTATACAAGGTGCCAGGAGTAGGTGGTCAGTTCCATCACGCATCGTCGCCAGCAGAACACGTCTATACGGATGAGCAGGGGATCACGTATGTGCACAAAAAAGACGCTGGAGGACCAGGAACACATACGCTTAGAGGACCAGGTCTAGCTGCTGTACAGCCCGCCTACTCCACTGTGCAGCCTGCGGGACTTCGCAATCCCCAGTTTCATGTGGAGCGAAGTGGTCGCACTGTGGATGTTGGTTCTGGAGGATTTGTCGTCCAAAGGAGCAGACGTAGTCCTCAGTTCCATGTGGAGCGTCCTGGTCGCACTGTGGATGTGGGTTCCGGAGGATTTTTTGTCCAAAGGGGCAGGCGTAGTCCCCAGTTTCCTGTGGAGCGACCTTGGCAAAGGGTCGACGTCGGCTTTAAGGGAC>line_528ATGGTCTCGCCCAAACGGAGACTAATCGACGATTCCATTGCTCCCTCAGCTGCCGATCTCCAGCGGACATACGACGGACATGATGGGCCTCACGTTTTCGGAACACCCGGCAATCAAGTCTACATTCGGGGGCAGAACGATGGCGTATACAAGGTGCCAGGAGTAGGTGGTCAGTTCCATCACGCATCGTCGCCAGCAGAACACGTCTATACGGATGAGCAGGGGATCACGTATGTGCACAAAAAAGACGCTGGAGGACCAGGAACACATACGCTTAGAGGACCAGGTCTAGCTGCTGTACAGCCCGCCTACTCCACTGTGCAGCCTGCGGGACTTCGCAATCCCCAGTTTCATGTGGAGCGAAGTGGTCGCACTGTGGATGTTGGTTCTGGAGGATTTGTCGTCCAAAGGAGCAGACGTAGTCCTCAGTTCCATGTGGAGCGTCCTGGTCGCACTGTGGATGTGGGTTCCGGAGGATTTTTTGTCCAAAGGGGCAGGCGTAGTCCCCAGTTTCCTGTGGAGCGACCTTGGCAAAGGGTCGACGTCGGCTTTAAGGGAC>line_563ATGGTCTCGCCCAAACGGAGACTAATCGACGATTCCATTGCTCCCTCAGCTGCCGATCTCCAGCGGACATACGACGGACATGATGGGCCTCACGTTTTCGGAACACCCGGCAATCAAGTCTACATTCGGGGGCAGAACGATGGCGTATACAAGGTGCCAGGAGTAGGTGGTCAGTTCCATCACGCATCGTCGCCAGCAGAACACGTCTATACGGATGAGCAGGGGATCACGTATGTGCACAAAAAAGACGCTGGAGGACCAGGAACACATACGCTTAGAGGACCAGGTCTAGCTGCTGTACAGCCCGCCTACTCCACTGTGCAGCCTGCGGGACTTCGCAATCCCCAGTTTCATGTGGAGCGAAGTGGTCGCACTGTGGATGTTGGTTCTGGAGGATTTGTCGTCCAAAGGAGCAGACGTAGTCCTCAGTTCCATGTGGAGCGTCCTGGTCGCACTGTGGATGTGGGTTCCGGAGGATTTTTTGTCCAAAGGGGCAGGCGTAGTCCCCAGTTTCCTGTGGAGCGACCTTGGCAAAGGGTCGACGTCGGCTTTAAGGGAC>line_821ATGGTCTCGCCCAAACGGAGACTAATCGACGATTCCATTGCTCCCTCAGCTGCCGATCTCCAGCGGACATACGACGGACATGATGGGCCTCACGTTTTCGGAACACCCGGCAATCAAGTCTACATTCGGGGGCAGAACGATGGCGTATACAAGGTGCCAGGAGTAGGTGGTCAGTTCCATCACGCATCGTCGCCAGCAGAACACGTCTATACGGATGAGCAGGGGATCACGTATGTGCACAAAAAAGACGCTGGAGGACCAGGAACACATACGCTTAGAGGACCAGGTCTAGCTGCTGTACAGCCCGCCTACTCCACTGTGCAGCCTGCGGGACTTCGCAATCCCCAGTTTCATGTGGAGCGAAGTGGTCGCACTGTGGATGTTGGTTCTGGAGGATTTGTCGTCCAAAGGAGCAGACGTAGTCCTCAGTTCCATGTGGAGCGTCCTGGTCGCACTGTGGATGTGGGTTCCGGAGGATTTTTTGTCCAAAGGGGCAGGCGTAGTCCCCAGTTTCCTGTGGAGCGACCTTGGCAAAGGGTCGACGTCGGCTTTAAGGGAC>line_348ATGGTCTCGCCCAAACGGAGACTAATCGACGATTCCATTGCTCCCTCAGCTGCCGATCTCCAGCGGACATACGACGGACATGATGGGCCTCACGTTTTCGGAACACCCGGCAATCAAGTCTACATTCGGGGGCAGAACGATGGCGTATACAAGGTGCCAGGAGTAGGTGGTCAGTTCCATCACGCATCGTCGCCAGCAGAACACGTCTATACGGATGAGCAGGGGATCACGTATGTGCACAAAAAAGACGCTGGAGGACCAGGAACACATACGCTTAGAGGACCAGGTCTAGCTGCTGTACAGCCCGCCTACTCCACTGTGCAGCCTGCGGGACTTCGCAGTCCCCAGTTTCATGTGGAGCGAAGTGGTCGCACTGTGGATGTTGGTTCTGGAGGATTTGTCGTCCAAAGGAGCAGACGTAGTCCTCAGTTCCATGTGGAGCGTCCTGGTCGCACTGTGGATGTGGGTTCCGGAGGATTTTTTGTCCAAAGGGGCAGGCGTAGTCCCCAGTTTCCTGTGGAGCGACCTTGGCAAAGGGTCGACGTCGGCTTTAAGGGAC>line_350ATGGTCTCGCCCAAACGGAGACTAATCGACGATTCCATTGCTCCCTCAGCTGCCGATCTCCAGCGGACATACGACGGACATGATGGGCCTCACGTTTTCGGAACACCCGGCAATCAAGTCTACATTCGGGGGCAGAACGATGGCGTATACAAGGTGCCAGGAGTAGGTGGTCAGTTCCATCACGCATCGTCGCCAGCAGAACACGTCTATACGGATGAGCAGGGGATCACGTATGTGCACAAAAAAGACGCTGGAGGACCAGGAACACATACGCTTAGAGGACCAGGTCTAGCTGCTGTACAGCCCGCCTACTCCACTGTGCAGCCTGCGGGACTTCGCAGTCCCCAGTTTCATGTGGAGCGAAGTGGTCGCACTGTGGATGTTGGTTCTGGAGGATTTGTCGTCCAAAGGAGCAGACGTAGTCCTCAGTTCCATGTGGAGCGTCCTGGTCGCACTGTGGATGTGGGTTCCGGAGGATTTTTTGTCCAAAGGGGCAGGCGTAGTCCCCAGTTTCCTGTGGAGCGACCTTGGCAAAGGGTCGACGTCGGCTTTAAGGGAC>line_358ATGGTCTCGCCCAAACGGAGACTAATCGACGATTCCATTGCTCCCTCAGCTGCCGATCTCCAGCGGACATACGACGGACATGATGGGCCTCACGTTTTCGGAACACCCGGCAATCAAGTCTACATTCGGGGGCAGAACGATGGCGTATACAAGGTGCCAGGAGTAGGTGGTCAGTTCCATCACGCATCGTCGCCAGCAGAACACGTCTATACGGATGAGCAGGGGATCACGTATGTGCACAAAAAAGACGCTGGAGGACCAGGAACACATACGCTTAGAGGACCAGGTCTAGCTGCTGTACAGCCCGCCTACTCCACTGTGCAGCCTGCGGGACTTCGCAGTCCCCAGTTTCATGTGGAGCGAAGTGGTCGCACTGTGGATGTTGGTTCTGGAGGATTTGTCGTCCAAAGGAGCAGACGTAGTCCTCAGTTCCATGTGGAGCGTCCTGGTCGCACTGTGGATGTGGGTTCCGGAGGATTTTTTGTCCAAAGGGGCAGGCGTAGTCCCCAGTTTCCTGTGGAGCGACCTTGGCAAAGGGTCGACGTCGGCTTTAAGGGAC
