# Supplementary material for: Repeated truncation of a modular antimicrobial peptide gene for neural context
Source: PLoS Genet. 2022 Jun 17;18(6):e1010259. doi: 10.1371/journal.pgen.1010259 (PMC9246212; doi:10.1371/journal.pgen.1010259)
Supplement: S3 Data — (ZIP) [file pgen.1010259.s010.zip › Supp data file 2/ATP8A locus/aBSREL/Datamonkey Adaptive Evolution Server_files/widget_iframe.06c6ee58c3810956b7509218508c7b56.html]

 
Twitter Widget Iframe
