# Supplementary material for: Repeated truncation of a modular antimicrobial peptide gene for neural context
Source: PLoS Genet. 2022 Jun 17;18(6):e1010259. doi: 10.1371/journal.pgen.1010259 (PMC9246212; doi:10.1371/journal.pgen.1010259)
Supplement: S3 Data — (ZIP) [file pgen.1010259.s010.zip › Supp data file 2/README.rtf]

HyPhy analysis outputs (Datamonkey webserver outputs) are stored for each gene locus in a separate folder. A summary of key results is also provided in each folder, copied from the Datamonkey webserver output (and these results can be found within excel files of these folders).
