# Supplementary material for: Repeated truncation of a modular antimicrobial peptide gene for neural context
Source: PLoS Genet. 2022 Jun 17;18(6):e1010259. doi: 10.1371/journal.pgen.1010259 (PMC9246212; doi:10.1371/journal.pgen.1010259)
Supplement: Attachment — Submitted filename: 124487_1_rebuttal_2319129_r8ysyw.docx [file pgen.1010259.s013.docx]

**Manuscript number:** RC-xx-xx

**Corresponding author(s):** First name, Last name

*[Please use this template only if the submitted manuscript should be considered by the affiliate journal as a full revision in response to the points raised by the reviewers.*

*If you wish to submit a preliminary revision with a revision plan, please use our "*[*Revision Plan*](https://docs.google.com/document/u/0/d/1HHp62Tfeb44nd3Gu_3ChB0b8gmMdEog7GjZcZFwHSiQ/edit)*" template.* ***It is important to use the appropriate template to clearly inform the editors of your intentions.****]*

## 1. General Statements [optional]

Overall we were elated to have received such positive comments on the manuscript, with requests for only minor changes. We have made all suggested changes to clarify or tone down the language as suggested.

We would like to thank each of the three reviewers for their assessment of our work. We note that all three reviewers agreed the phylogenetic analysis was interesting and convincing. Two of the three reviewers felt the study sufficiently demonstrated roles for Baramicin in the nervous system. We have responded to comments from Reviewer 2 to draw attention to some aspects of the data that they may have overlooked, which we hope reassures them that our proposal of BaraB and BaraC involvement in the nervous system is robust, coming from different approaches that show consistent results.

Reviewer 1 and Reviewer 3 compliment the study as being very worthwhile, and for suggesting concrete routes for how an AMP evolved non-immune functions. Both compliment its comprehensiveness, and describe the study as having ‘striking findings’ that ‘should have broad appeal to audiences interested in the crosstalk between the nervous system and the innate immune system.’

## 2. Point-by-point description of the revisions

In the revised manuscript file, we have highlighted all text where changes were made.

------------------------------------------------------------------------------
Reviewer #1 (Evidence, reproducibility and clarity (Required)):

The authors provide convincing evidence for an evolutionary scenario in which duplications of an AMP gene with ancestral immune function led to paralogs specialist for neural functions. They focus on the Baramicin genes, coding for major Toll signalling targets in the context of antifungal defence. Their study uses infection experiments in several Drosophila species, a careful annotation of the Baramicin genes of D. melanogaster, the demonstration of neural expression of BaraB and BaraC, the KD analysis of Bara B revealing lethality and neurological phenotypes, a reconstruction of the evolutionary history of Baramicn genes in Drosophilids and an analysis of the sequence evolution of the IM24 domain providing the neural functions.
In general the paper is well written. There are a few places in the manuscript where the language can be improved and one point, which needs clarification:
- ine 297: ...,which did not present with...
- line 314/315: ...to just 14% that of...to 63% that of
- line 459: ..., we this motif...
- line 518: What does "... genomic relatedness (by speciation and locus)..." mean?
- line 527/528: ...drive behaviour or disease through interactions...
- line 532: ... ancestrally encodes distinct peptides involved with either the nervous system or the immune response... line 535: ...with either the nervous system (IM24) or....
Do the data provide enough evidence suggesting that IM24 had a neural function in the ancestor? Ideally the authors should look at neural expression of the Baramicin gene in the ourgroup, S. lebanonensis. The authors later (line571) admit, that they cannot rule out that IM24 is also antimicrobial.

We thank reviewer #1 for drawing attention to these points. We have made changes to each line to be more concise, clarify our meaning, or fix typos.

Reviewer #1 (Significance (Required)):

This is a very comprehensive study, which, to my knowledge for the first time, suggests concrete routes of how an AMP evolved non-immune functions.
One of the striking findings of this paper is that duplications and subsequent truncations of the ancestral Baramicin locus linked to specialisation for neural functions occurred independently in different Drosophila lineages.

We thank reviewer #1 for their very positive comments. We also agree with all suggested changes, including more careful phrasing to emphasize that we have not described a mechanism, just an involvement in the nervous system. For instance, see lines 556-568 are reworked to soften language and explicitly state the ancestral function of IM24 is unknown, and our suggestion that IM24 could underlie Dmel\BaraA interactions with the nervous system is speculation that should be tested.

Reviewer #2 (Evidence, reproducibility and clarity (Required)):

Hanson and Lemaitre present a genomic and phylogenetic characterization of the Baramicin family of antimicrobial peptide genes in different species. They discover new Baramicin paralogs, united by the presence of an IM24 domain at the N-terminus. They show that among Baramicins, those that are not inducible by infection (which they improperly call non-immune since a protein can be non-inducible by infection and have very important immune functions), are truncated. They propose that an ancestor peptide with immune functions evolved into a neuronal regulator/effector via truncation.

Although the hypothesis is interesting, the data do not really support it. This manuscript is rather descriptive at this point. The demonstration that IM24 is necessary for neural function is very tenuous. For example, in the paragraphs titled Dmel\BaraB is required in the nervous system during development and Baramicin B plays an important role in the nervous system, I did not find convincing data demonstrating that BaraB is required in the nervous system. The only data that links BaraB to the nervous system is a weak locomotion defect observed in the BaraB mutant. But how many genes, when inactivated, give a locomotion defect? This remains totally unexplained at the molecular level. The authors also mentioned that BaraB is expressed in a subset of mechanosensory neuron cells in the wing. What is the link between this expression and the nubbin phenotype?
The authors also mention that data in the literature indicate that BaraC is expressed in glial cells but also in other tissues.
Finally, we have no idea what role, if any, these peptides have in the nervous system.

While the characterization of the Baramicin gene family and its evolution across species is convincing, the link between these AMPs and the nervous system is really too preliminary to be convincing.
The manuscript would greatly benefit from being more concise.

Reviewer #2 (Significance (Required)):

see above

We thank reviewer #2 for their fair assessment. We have made edits to soften our phrasing, and to emphasize that we have not described a mechanism, just an involvement, in the nervous system.

Examples:

line 270: “integral development role” -> “important for development”

line 277: “*Baramicin B plays an important role in the nervous system*“ -> “*Baramicin B suppression in the nervous system mimics mutant phenotypes”*

line 532: “Here we demonstrate that the *Baramicin* antimicrobial peptide gene of *Drosophila* ancestrally encodes distinct peptides involved with either the nervous system or the immune response.“ -> “Here we demonstrate that the *Baramicin* antimicrobial peptide gene of *Drosophila* ancestrally encodes distinct peptides that may interact with either the nervous system (IM24) or invading pathogens (IM10-like, IM22).”

line 562 new text: “Thus while our results suggest that IM24 of different *Baramicin* genes might underlie *Baramicin* interactions with the nervous system, we cannot exclude the possibility that IM24 is also antimicrobial, or even that antimicrobial activity is IM24’s ancestral purpose. Future studies could use tagged IM24 transgenes or synthetic peptides to determine the host binding partner(s) of secreted IM24 from the immune-induced *Dmel\BaraA*, and/or to see if IM24 binds to microbial membranes.”

We have also changed all instances of “non-immune Baramicins” to “Baramicins lacking immune induction” or something to that effect (e.g. new Lines 25,464, 469,478-82).

We also made some small changes to be more concise (e.g. line 387, 447, cut lines 492-495 from previous version, cut lines 506-507 from previous version).

We have responded below in the reviewer-to-reviewer comments for a few of the specific points raised there, which we hope further assuage some of Reviewer 2’s concerns.

Reviewer #3 (Evidence, reproducibility and clarity (Required)):

Antimicrobial peptides are main effectors in (insect) immune defenses. It is becoming more and more clear, that AMPs can have pleiotropic effects or even acquire new functions.
In the present paper, the authors investigate Baramicin, an antifungal AMP that they described first in publication last year. Here they show that in Drosophila melanogaster Baramicin A, which they described before, has paralogs, that are not immune-inducible. They then show that these paralogs, named BarB and BarC, which are truncated versions of BarA, are expressed in the head and neural tissues. That they have neural functions is supported by targeted gene-silencing experiments.
They go on to show, using a comparative approach across Drosophila, that Baramicin A with its antimicrobial function constitutes the ancestral state. Moreover, Baramicin is also enriched in head samples of some of the other Drosophila species they study. This manuscript, which according to the acknowledgements has already been seen by reviewers, is in a very good shape.

I have only a number of minor points, that might help to clarify the presentation.

Lines 34-36: I would delete this sentence and replace it with a statement based on the main findings of the manuscript

We now conclude the abstract with “As many AMP genes encode polypeptides, a full understanding of how immune effectors interact with the nervous system will require consideration of all their peptide products.”

Lines 56-60. May be tone down a bit. Anti-inflammatory activities of AMPs have been known for a long time. I think the next paragraph makes a very good case what is already known and is hence a nice motivation for the current study.

Toned down. This part now reads: “However AMPs and AMP-like genes in many species have recently been implicated in non-immune roles in flies, nematodes, and humans, suggesting non-immune functions might help explain AMP evolutionary patterns.”

Line 125: classical instead of classically

done

Line 200: what is a 'novel' time course? I would just describe what has been done.

Now reads: “We next measured *Baramicin* expression over development from egg to adult.”

Line 268: hypomorph, I guess in the literature usually hypomorphic is used.

done

Line 279: I would suggest to tone this headline down. This is not a criticism of the paper, but the actual mechanisms of the roles in the nervous system are not studied here.

Done. Now reads: “*Baramicin B suppression in the nervous system mimics mutant phenotypes”*

Line 505: what does not really become clear is whether IM24 plays an important role in the nervous system of fly species that only have BarA.

Edits from lines 556-568 now help highlight this question.

Line 540-549. This comparison I find a bit far-fetched, or maybe it needs clarification how doublesex expression is related to Baramicins.

Being completely honest: the doublesex discussion was requested during previous review at another journal. We agree that it is a bit of a tangent, and so we have removed these sentences.

Line 584-585. I think that this has been known for much longer from studies in frogs and beetles.

Our use of “in vivo” might have been a bit squishy here. We have edited this to reflect endogenous loss-of-function study, rather than simply “in vivo,” to clarify our intended sentiment.

Reviewer #3 (Significance (Required)):

Overall, I think that this is a very worthwhile and convincing story about the evolution AMPs and how they can acquire new functions. All the main statements are supported by careful experiments and data analysis. The paper does not go into any detail, of how the neurological role of BarB and BarC is achieved, but I think this is beyond the scope of the current manuscript.
In short, this is a very worthwhile contribution to the growing literature of the role of AMPs in the nervous system. The authors provide the context of the main published papers in the area in the introduction. As opposed to most papers on this so far, the current manuscript also provides very interesting data on the evolutionary history of the Baramicin genes, both within the main study species, and within other Drosophila species.
This paper should appeal to a rather broad audience of researchers interested in innate defenses, AMPs and the crosstalk between the nervous system and the innate immune system.


My background is insect immunology with a focus on AMPs and evolutionary approach.

We thank reviewer #3 for their very positive comments. We agree with all suggested changes.

**Referees cross-commenting**

This session contains the comments of all reviewers

Reviewer 3

Reviewer 2 and I share the view, that the evidence for the effects of BarB and C on the nervous system is rather limited. But I still think, that the paper provides enough new and interesting data that make it a very useful contribution. Though not a neurobiologist, I would assume that providing functional evidence for the role of BarA and B in the nervous system would justify a paper on its own. I agree though, that the relevant sections should be toned down.

Reviewer 2

As I mentioned in my review, I found the genomic and phylogenetic analysis interesting and convincing. I therefore totally agréé with reviewers 2 and 3 on that. Whether BarA and B are playing a role in the nervous system and how it does remain speculative. BaraB mutants show locomotion defects. But mutants in mitochondrial genes have locomotion defects. Can we conclude that mitochondria play a role in the nervous system? If I understand correctly, downregulating Bara in neurons only (With Elav-Gal4 driver) does not show the locomotion phenotype. it induces early lethality. How many genes when inactivated in neurons will give rise to such a phenotype? A lot. I really think that the implication of Bara in the nervous system should be seriously toned done and more presented as an hypothesis than a validated fact.

We would like to note for Reviewer 2 here that it is specifically elav> BaraB-IR that results in lethality, and in weaker gene silencing experiments, adult elav>BaraB-IR flies emerge, and they do suffer locomotor defects. Often, they got stuck in the food shortly after emerging, or would move haphazardly (which was common in flies with nubbin-like wings). We have added explicit mention that elav>BaraB-IR also results in locomotor defects (Line 288-289).

*Our private speculation is that the reason flies fail to emerge from their pupae is because they are so uncoordinated that they sometimes cannot wriggle out of the pupal case before their cuticle hardens. In some instances, both using mutants and RNAi, we observed fully developed adults with mature abdominal pigmentation that died trapped inside their pupal cases.*

We’d also like to emphasize here that despite testing many other Gal4 drivers, including mef2-Gal4 (muscle/myocytes), nubbin-like wings and lethality were only found using elav-Gal4. A role interacting with mitochondria would likely have been revealed using mef2-Gal4, given the importance of mitochondrial function in muscle.

For BaraC: expression in other tissues (like the rectal pad) could nevertheless be from e.g. nerves innervating the muscles controlling the sphincter. Or it could indeed be entirely unrelated to the nervous system. However we feel the nearly perfect overlap with Repo-expressing cells is a strong argument for a neural role. We also made an effort using RNAi to validate this pattern suggested by scRNAseq, which confirmed a strong knockdown of BaraC-IR with Repo-Gal4 (Fig. 3, Fig. S4).

We hope these comments clarify for Reviewer 2 why we feel confident in proposing a role for Baramicins in the nervous system, even if we do not investigate a mechanism in this study.

Reviewer 1

I agree with reviewer 3 that the main message of the paper providing a concrete scenario of how non-immune functions of AMPs may evolve is an important contribution. A deep investigation of the neural function is definitely going beyond the scope of the paper. Indeed this might be quite tricky. But it would help if the authors could clarify their idea about the ancestral condition. Is there the possibility that IM24 had ancestrally already non-immune function? They are not really clear about this point.

Reviewer 2

I agree with the other reviewers that determining the exact role of Bara peptides could be complicated. I just ask that the authors limit themselves to proposing that the peptides have lost their immune function. I stress that this argument is not very strong. It relies solely on the lack of inducibility of these peptides following infection. I still think that the demonstration of the role of Bara in the nervous system is not provided.
