# Supplementary material for: Repeated truncation of a modular antimicrobial peptide gene for neural context
Source: PLoS Genet. 2022 Jun 17;18(6):e1010259. doi: 10.1371/journal.pgen.1010259 (PMC9246212; doi:10.1371/journal.pgen.1010259)
Supplement: Attachment — Submitted filename: ResponseBaraNeuralEvol_May6-2022.docx [file pgen.1010259.s014.docx]

*We have made all changes requested.*

*-Mark Hanson and Bruno Lemaitre*

1) Please ensure that all author names are spelled correctly in the manuscript file, including the use of a middle name/initials, and any special characters (accents, umlauts, etc.). Please place first/given names before last/family names.  All authors must have a first and last name. We noticed that "Mark A Hanson, Bruno Lemaitre" are spelled differently between the manuscript file and submission form. Please be aware that correct spelling and formatting of author names in the article file is vital for accurate indexing in PubMed.

*Done*

2) Please provide an Author Summary. This should appear in your manuscript between the Abstract (if applicable) and the Introduction, and should be 150–200 words long. The aim should be to make your findings accessible to a wide audience that includes both scientists and non-scientists. Sample summaries can be found on our website under Submission Guidelines:
<https://journals.plos.org/plosgenetics/s/submission-guidelines#loc-parts-of-a-submission>

*Done*

3) We do not publish any copyright or trademark symbols that usually accompany proprietary names, eg (R), (C), or TM  (e.g. next to drug or reagent names). Please remove all instances of trademark/copyright symbols throughout the text, including TRIzol™ on line no 603.

*Done*

4) We have noticed that you have uploaded " SupplementaryDataFiles.zip" files as supporting information but you have not included them in the list of legends.  Please add a citation of this SI data files into the list of legends of your supporting information files.

*Done*

5) Please amend your detailed Financial Disclosure statement. This is published with the article, therefore should be completed in full sentences and contain the exact wording you wish to be published.
i). State the initials, alongside each funding source, of each author to receive each grant.
ii). State what role the funders took in the study. If the funders had no role in your study, please state: “The funders had no role in study design, data collection and analysis, decision to publish, or preparation of the manuscript.”
iii). If any authors received a salary from any of your funders, please state which authors and which funders.

*Done*
