# Supplementary material for: Repeated truncation of a modular antimicrobial peptide gene for neural context
Source: PLoS Genet. 2022 Jun 17;18(6):e1010259. doi: 10.1371/journal.pgen.1010259 (PMC9246212; doi:10.1371/journal.pgen.1010259)
Supplement: Peer review history

# Author comment: Author response to previous submission

**DOI:** 10.1371/journal.pgen.1010259.r001

1 Apr 2022

Attachment Submitted filename: 124487_1_rebuttal_2319129_r8ysyw.docx Click here for additional data file.

# Editor report: Decision Letter 0

**DOI:** 10.1371/journal.pgen.1010259.r002

**Authors:** Lynn M Riddiford, Kirsten Bomblies

24 Apr 2022

Dear Dr Hanson,

Thank you very much for submitting your Research Article entitled 'Repeated truncation of a modular antimicrobial peptide gene for neural context' to PLOS Genetics.

The revised manuscript and the associated peer reviews were fully evaluated at the editorial level. The editor  felt that the revisions you had made according to the reviewers' criticisms were adequate but that there were a few minor revisions yet necessary before publication. 

We therefore ask you to modify the manuscript according to the editor's comments below. Your revisions should address the specific points.

In addition we ask that you:

1) Provide a detailed list of your responses to the review comments and a description of the changes you have made in the manuscript.

2) Upload a Striking Image with a corresponding caption to accompany your manuscript if one is available (either a new image or an existing one from within your manuscript). If this image is judged to be suitable, it may be featured on our website. Images should ideally be high resolution, eye-catching, single panel square images. For examples, please browse our archive. If your image is from someone other than yourself, please ensure that the artist has read and agreed to the terms and conditions of the Creative Commons Attribution License. Note: we cannot publish copyrighted images.

We hope to receive your revised manuscript within the next 30 days. If you anticipate any delay in its return, we would ask you to let us know the expected resubmission date by email to plosgenetics@plos.org.

If present, accompanying reviewer attachments should be included with this email; please notify the journal office if any appear to be missing. They will also be available for download from the link below. You can use this link to log into the system when you are ready to submit a revised version, having first consulted our Submission Checklist.

While revising your submission, please upload your figure files to the Preflight Analysis and Conversion Engine (PACE) digital diagnostic tool. PACE helps ensure that figures meet PLOS requirements. To use PACE, you must first register as a user. Then, login and navigate to the UPLOAD tab, where you will find detailed instructions on how to use the tool. If you encounter any issues or have any questions when using PACE, please email us at figures@plos.org.

Please be aware that our data availability policy requires that all numerical data underlying graphs or summary statistics are included with the submission, and you will need to provide this upon resubmission if not already present. In addition, we do not permit the inclusion of phrases such as "data not shown" or "unpublished results" in manuscripts. All points should be backed up by data provided with the submission.

To enhance the reproducibility of your results, we recommend that you deposit your laboratory protocols in protocols.io, where a protocol can be assigned its own identifier (DOI) such that it can be cited independently in the future. Additionally, PLOS ONE offers an option to publish peer-reviewed clinical study protocols. Read more information on sharing protocols at https://plos.org/protocols?utm_medium=editorial-email&utm_source=authorletters&utm_campaign=protocols

Please review your reference list to ensure that it is complete and correct. If you have cited papers that have been retracted, please include the rationale for doing so in the manuscript text, or remove these references and replace them with relevant current references. Any changes to the reference list should be mentioned in the rebuttal letter that accompanies your revised manuscript. If you need to cite a retracted article, indicate the article’s retracted status in the References list and also include a citation and full reference for the retraction notice.

PLOS has incorporated Similarity Check, powered by iThenticate, into its journal-wide submission system in order to screen submitted content for originality before publication. Each PLOS journal undertakes screening on a proportion of submitted articles. You will be contacted if needed following the screening process.

To resubmit, you will need to go to the link below and 'Revise Submission' in the 'Submissions Needing Revision' folder.

[LINK]

Please let us know if you have any questions while making these revisions.

Yours sincerely,

Lynn M Riddiford

Associate Editor

PLOS Genetics

Kirsten Bomblies

Section Editor: Evolution

PLOS Genetics

This revised manuscript has answered the reviewers’ concerns. It is an interesting paper with new information about this family of genes, both its evolution and the potential neofunctionalization of derivative peptides during evolution. It clearly is appropriate for publication in PloS Genetics.

As the Editor, I have the following minor concerns that should be fixed before publication:

1) “Mendelian” should be capitalized wherever used in the text.

2) Figure 3E: Are the wings really like the nubbin mutant phenotype with clear developmental defects? Or are they simply only partially expanded after eclosion? To me this picture shows a partially expanded wing rather than the nubbin phenotype that is pictured in the paper describing this mutant by Ng et al. (1995) (Development 121, 589-599; see Figure 2). Nubbin mutant wings normally expand but they are deformed due to developmental defects. If the wings of your BaraB RNAi flies do not have any of the nubbin hinge and patterning defects, you should not call them “nubbin-like”. Rather just refer to them as partially expanded wings. Such expansion defects are often seen when flies have trouble emerging. Similarly, the locomotion defects could be due to problems in eclosion preventing the legs from expanding completely before they sclerotize and thus their inability to move normally.

3) line 296: should be “myo”, not “my0”. The myo-Gal4 driver expresses in embryonic and larval glia according to Awasaki et al. (2011). The glia secrete myoglianin to instruct neuron remodelling at metamorphosis. Please cite the reference or your data that indicates that this driver also can drive expression in the gut. You should also add the myo data to your negative evidence for the necessity of Bara-B expression neural tissues.

# Author comment: Author response to Decision Letter 0

**DOI:** 10.1371/journal.pgen.1010259.r003

6 May 2022

Attachment Submitted filename: ResponseBaraNeuralEvol_May6-2022.docx Click here for additional data file.

# Editor report: Decision Letter 1

**DOI:** 10.1371/journal.pgen.1010259.r004

**Authors:** Lynn M Riddiford, Kirsten Bomblies

17 May 2022

Dear Dr Hanson,

We are pleased to inform you that your manuscript entitled "Repeated truncation of a modular antimicrobial peptide gene for neural context" has been editorially accepted for publication in PLOS Genetics. Congratulations!

Before your submission can be formally accepted and sent to production you will need to complete the corrections noted below and our formatting changes, which you will receive in a follow up email. Please be aware that it may take several days for you to receive this email; during this time no action is required by you. Please note: the accept date on your published article will reflect the date of this provisional acceptance, but your manuscript will not be scheduled for publication until the required changes have been made.

Once your paper is formally accepted, an uncorrected proof of your manuscript will be published online ahead of the final version, unless you’ve already opted out via the online submission form. If, for any reason, you do not want an earlier version of your manuscript published online or are unsure if you have already indicated as such, please let the journal staff know immediately at plosgenetics@plos.org.

In the meantime, please log into Editorial Manager at https://www.editorialmanager.com/pgenetics/, click the "Update My Information" link at the top of the page, and update your user information to ensure an efficient production and billing process. Note that PLOS requires an ORCID iD for all corresponding authors. Therefore, please ensure that you have an ORCID iD and that it is validated in Editorial Manager. To do this, go to ‘Update my Information’ (in the upper left-hand corner of the main menu), and click on the Fetch/Validate link next to the ORCID field.  This will take you to the ORCID site and allow you to create a new iD or authenticate a pre-existing iD in Editorial Manager.

If you have a press-related query, or would like to know about making your underlying data available (as you will be aware, this is required for publication), please see the end of this email. If your institution or institutions have a press office, please notify them about your upcoming article at this point, to enable them to help maximise its impact. Inform journal staff as soon as possible if you are preparing a press release for your article and need a publication date.

Thank you again for supporting open-access publishing; we are looking forward to publishing your work in PLOS Genetics!

Yours sincerely,

Lynn M Riddiford

Associate Editor

PLOS Genetics

Kirsten Bomblies

Section Editor: Evolution

PLOS Genetics

www.plosgenetics.org

Twitter: @PLOSGenetics

----------------------------------------------------

Comments from the reviewers (if applicable):

This revised paper is now acceptable for publication. There still are some minor corrections needed as listed below:

1) line 79: nemuri should be italicized

2) check the following lines for the correct formatting of boldface for the figure numbers: lines 189,200,236,270,391,410,579,608.

3) Usually in the text, names of amino acids are not capitalized: see lines 394, 396, 397, 398, 405, 406, 411, 440, 441, 443.

4) References:

a) Be sure to italicize genus and species names in the titles.

b) Titles should be in sentence case format, not with capital letters except the first word and genus names: see Clemmons, Kobler, Lezi, Swanson, Unckless,

c) some references are missing volume and/or page numbers: see line 794, line 725, Sackler, Sunganoyagan, Suvorov, Tennessen, Wang,J., Wang, Y.

----------------------------------------------------

**Data Deposition**

If you have submitted a Research Article or Front Matter that has associated data that are not suitable for deposition in a subject-specific public repository (such as GenBank or ArrayExpress), one way to make that data available is to deposit it in the Dryad Digital Repository. As you may recall, we ask all authors to agree to make data available; this is one way to achieve that. A full list of recommended repositories can be found on our website.

The following link will take you to the Dryad record for your article, so you won't have to re‐enter its bibliographic information, and can upload your files directly: 

http://datadryad.org/submit?journalID=pgenetics&manu=PGENETICS-D-22-00410R1

More information about depositing data in Dryad is available at http://www.datadryad.org/depositing. If you experience any difficulties in submitting your data, please contact help@datadryad.org for support.

Additionally, please be aware that our data availability policy requires that all numerical data underlying display items are included with the submission, and you will need to provide this before we can formally accept your manuscript, if not already present.

----------------------------------------------------

**Press Queries**

If you or your institution will be preparing press materials for this manuscript, or if you need to know your paper's publication date for media purposes, please inform the journal staff as soon as possible so that your submission can be scheduled accordingly. Your manuscript will remain under a strict press embargo until the publication date and time. This means an early version of your manuscript will not be published ahead of your final version. PLOS Genetics may also choose to issue a press release for your article. If there's anything the journal should know or you'd like more information, please get in touch via plosgenetics@plos.org.

# Editor report: Acceptance letter

**DOI:** 10.1371/journal.pgen.1010259.r005

**Authors:** Lynn M Riddiford, Kirsten Bomblies

8 Jun 2022

PGENETICS-D-22-00410R1 

Repeated truncation of a modular antimicrobial peptide gene for neural context 

Dear Dr Hanson, 

We are pleased to inform you that your manuscript entitled "Repeated truncation of a modular antimicrobial peptide gene for neural context" has been formally accepted for publication in PLOS Genetics! Your manuscript is now with our production department and you will be notified of the publication date in due course.

The corresponding author will soon be receiving a typeset proof for review, to ensure errors have not been introduced during production. Please review the PDF proof of your manuscript carefully, as this is the last chance to correct any errors. Please note that major changes, or those which affect the scientific understanding of the work, will likely cause delays to the publication date of your manuscript. 

Soon after your final files are uploaded, unless you have opted out or your manuscript is a front-matter piece, the early version of your manuscript will be published online. The date of the early version will be your article's publication date. The final article will be published to the same URL, and all versions of the paper will be accessible to readers.

Thank you again for supporting PLOS Genetics and open-access publishing. We are looking forward to publishing your work! 

With kind regards,

Agnes Pap

PLOS Genetics

On behalf of:

The PLOS Genetics Team

Carlyle House, Carlyle Road, Cambridge CB4 3DN | United Kingdom

plosgenetics@plos.org | +44 (0) 1223-442823

plosgenetics.org | Twitter: @PLOSGenetics
